# Use of the Digital Assistant Vigo in the Home Environment for Stroke Recovery: Focus Group Discussion With Specialists Working in Neurorehabilitation

**DOI:** 10.2196/44285

**Published:** 2023-04-14

**Authors:** Klinta Epalte, Aleksandrs Grjadovojs, Guna Bērziņa

**Affiliations:** 1 Department of Rehabilitation Riga Stradiņš University Riga Latvia

**Keywords:** stroke, rehabilitation, digital therapeutic, focus group, home-based rehabilitation, recovery, efficacy, application, rehabilitation program, functionality, usability, development

## Abstract

**Background:**

There is a lack of resources for the provision of adequate rehabilitation after a stroke, thus creating a challenge to provide the necessary high-quality, patient-centered, and cost-efficient rehabilitation services at a time when they are needed the most. Tablet-based therapeutic programs present an alternative way to access rehabilitation services and show a new paradigm for providing therapeutic interventions following a stroke anytime and anywhere. The digital assistant Vigo is an artificial intelligence–based app that provides an opportunity for a new, more integrative way of carrying out a home-based rehabilitation program. Considering the complexity of the stroke recovery process, factors such as a suitable population, appropriate timing, setting, and the necessary patient-specialist support structure need to be thoroughly researched. There is a lack of qualitative research exploring the perspectives of professionals working in neurorehabilitation of the content and usability of the digital tool for the recovery of patients after a stroke.

**Objective:**

The aim of this study is to identify the requirements for a tablet-based home rehabilitation program for stroke recovery from the perspective of a specialist working in stroke rehabilitation.

**Methods:**

The focus group study method was chosen to explore specialists’ attitudes, experience, and expectations related to the use of the digital assistant Vigo as a home-based rehabilitation program for stroke recovery in domains of the app’s functionality, compliance, usability, and content.

**Results:**

In total, 3 focus groups were conducted with a participant count of 5-6 per group and the duration of the discussion ranging from 70 to 80 minutes. In total, 17 health care professionals participated in the focus group discussions. The participants represented physiotherapists (n=7, 41.2%), occupational therapists (n=7, 41.2%), speech and language therapists (n=2, 11.8%), and physical medicine and rehabilitation physicians (n=1, 5.9%). Audio and video recordings of each discussion were created for further transcription and analysis. In total, 4 themes were identified: (1) the clinician’s views on using Vigo as a home-based rehabilitation system, (2) patient-related circumstances facilitating and limiting the use of Vigo; (3) Vigo’s functionality and use process (program creation, individual use, remote support); and (4) complementary and alternative Vigo use perspectives. The last 3 themes were divided further into 10 subthemes, and 2 subthemes had 2 sub-subthemes each.

**Conclusions:**

Health care professionals expressed a positive attitude toward the usability of the Vigo app. It is important that the content and use of the app be coherent with the aim to avoid (1) misunderstanding its practical use and the need for integration in practice and (2) misusing the app. In all focus groups, the importance of close involvement of rehabilitation specialists in the process of app development and research was highlighted.

## Introduction

During 2019, in Latvia, 5838 people were hospitalized because of a stroke [1]. More than 80% of these admissions were in stroke units, creating a heavy burden on specialists working in acute stroke care [1,2]. Unfortunately, there is a shortage of rehabilitation providers and a lack of resources for the provision of adequate rehabilitation after a stroke [2-4]. Thus, professionals and organizations are challenged to provide the necessary high-quality, patient-centered, and cost-efficient rehabilitation services at a time when they are needed the most. Increased pressure on hospitals and inpatient centers shows that new rehabilitation approaches need to be considered outside the hospital setting [5,6].

Home-based rehabilitation has the benefit of treating clients in a familiar environment, which stimulates mental and physical activity and prevents problems with transferring learned skills to their daily lives [4]. Research shows that home-based interventions can provide more cost-efficient services [7,8]. In recent years, the use of information and communication technologies (ICT) has been a research area of interest due to its potential to improve the efficiency, quality, and availability of rehabilitation care [9,10]. Tablet-based therapeutic programs present an alternative way to access rehabilitation services anytime and everywhere through the internet and technology under remote guidance of the therapist [11-13]. Tablet-based rehabilitation programs show a new paradigm for providing therapeutic interventions following a stroke [14]. Considering the complexity of the stroke recovery process, factors such as a suitable population, appropriate timing, setting, and the necessary patient-specialist support structure need to be thoroughly researched [15,16]. Research shows that personalized apps could give health professionals a better overview of patients’ rehabilitation process and provide follow-up after the patients are discharged from the inpatient rehabilitation center or stroke unit, with the condition that the apps would contain information about patients’ health status and functional impairment and the content would support the person-centered rehabilitation process [17,18].

At the time of discharge from the hospital or inpatient rehabilitation center, approximately 74% of physical and occupational therapists hand out a written home program. Even though written recommendations are a widely used approach for continuing rehabilitation at home, they lack 2 key components, adherence and feedback [14,19]. The digital assistant Vigo provides an opportunity for a new, more integrative way of carrying out a home-based rehabilitation program compared to traditional written recommendations. Previous research shows that patients have a positive attitude toward the use of the digital assistant Vigo as a tool for therapeutic home-based programs [20]. To integrate the digital assistant into practice, it is of upmost importance to research the use of the program, not only from a patient perspective, but also from the perspective of health care professionals working in neurorehabilitation. The aim of this study is to identify the requirements for a tablet-based home rehabilitation program for stroke recovery from the perspective of a specialist working in stroke rehabilitation.

## Methods

### Study Design

A qualitative exploratory study was conducted to identify the eligibility requirements of the tablet-based home rehabilitation program Vigo for stroke recovery from a specialist perspective. The focus group study method was chosen since this method is useful to gather information about the beliefs of a specific subgroup [21]. The main interest of concern is a better understanding of specialists’ attitudes, experience, and expectations related to the use of the digital assistant Vigo as a home-based rehabilitation program for stroke recovery in domains of the app’s functionality, compliance, usability, and content.

### Digital Assistant Vigo

The digital assistant Vigo is an artificial intelligence–based app suitable for installation on Apple iPad. The main goal of the app is to be a digital assistant to patients recovering from a stroke. It is intended to educate and give practical advice and exercises on stroke-related issues, rehabilitation, and care useful for both the patient and their family. The app is designed using chatbot and gamification elements to encourage participation in the patients’ individual daily plan that is adapted according to their functional status. Vigo comprises 3 modules: knowledge, skills, and motivation. Additionally, standardized cognitive behavioral therapy (CBT) methods and exercises are used to overcome anxiety, lack of motivation, and depressed mood [22].

Developers of the app describe Vigo as a tool that gives the patient an opportunity to immediately receive interventions intended to be part of stroke rehabilitation medical services (physiotherapy, speech and language therapy, occupational therapy, and psychological support) that are adjusted to be received through a digital device. Exercises provided in the program are in prerecorded video format. It is also stated that to use the app, most of the time the patient does not require help from another person and specialist consultations are needed to solve specific problems [23].

### Participants

Participants in this study were selected using a purposeful sampling strategy [24]. Health care specialists were invited to participate in the study if they met the following inclusion criteria: representing 1 of the rehabilitation professions (physiotherapist, occupational therapist, speech and language therapist, physical medicine, and rehabilitation physician) and being employed at a health care facility providing rehabilitation services after a stroke in the acute or subacute phase. Prospective participants were contacted directly or through a contact person at their workplace to provide an introduction to the study and an invitation to participate. Those who accepted the invitation and were eligible to participate received further information regarding the procedures of focus group discussions and confidentiality concerns of the study. Each participant was asked to get acquainted with publicly available information, as well as a manufacturer-provided description and demonstration video about Vigo. Participants were provided an opportunity to try Vigo on an Apple iPad mini with a manufacturer-provided demo patient profile.

### Data Collection

The data in this study were obtained by conducting remote focus group discussions via Zoom videoconferencing software during September and October 2022. The focus groups were moderated by the coauthor (AG), who has previous experience in conducting focus groups for health care research according to a previously designed focus group plan. The moderator of the discussions had no previous experience with the app and had no personal assumptions about the main questions of interest to ensure clarity of gathered data. Discussions were observed by the lead author (KE) assisting, where necessary, and taking notes about the process of the discussion. The lead author played the role of an observer due to their previous qualitative research experience in the usability of the app. A total of 3 focus groups were conducted, with a participant count of 5-6 per group and the duration of the discussion ranging from 70 to 80 minutes. Audio and video recordings of each discussion were created for further transcription and analysis.

Discussions were conducted based on a premade focus group guide containing open-ended questions regarding the Vigo app content, functionality, and user experience. The first focus group was considered as a pilot, and the selected focus group guide questions were modified based on observed participants’ responses in order to facilitate data collection.

### Ethical Considerations

The study complied with the General Data Protection Regulation and the Declaration of Helsinki and was approved by the Ethics Committee of Riga Stradins University, Latvia (no. 2-PĒK-4/487/2022).

Participants were informed about confidentiality concerns regarding their participation in the study before and at the start of each focus group and provided informed consent by expressing their intention to continue with the discussions.

### Data Analyses

Discussion recordings were transcribed verbatim according to predefined transcription rules. All sensitive information, such as participants’ names, workplace names, job positions, and locations, that could potentially reveal participants’ identity was edited out or replaced by more generic information. Transcripts and original recordings were imported in MaxQDA software, with each speech contribution coded by the participant’s ID, recording timestamp, and consecutive number within the focus group.

Data coding was performed in several iterations using an inductive approach to identify and systematically organize themes of the discussion according to the needs of the study. Data coding was performed by 1 of the coauthors.

Thematic data were extracted from all coded segments by rephrasing and summarizing a theme within the speech contribution according to designated codes. Such thematic extracts from all focus groups were organized within the structure of the main themes. Similarly, themed extracts were summarized within the structure of subthemes of the main themes. The resulting summary of themes and subthemes, with references to the original participants’ contributions, was used to describe the results of the study. The themes and summaries were discussed and reviewed between all authors of the study.

## Results

### Participant Characteristics

A total of 29 health care professionals working in neurorehabilitation were invited to participate in the study, 17 (58.6%) of whom accepted the invitation and participated in the focus group discussions. All participants were females. They represented physiotherapists (n=7, 41.2%), occupational therapists (n=7, 41.2%), speech and language therapists (n=2, 11.8%), and physical medicine and rehabilitation physicians (n=1, 5.9%). They were employed in inpatient rehabilitation centers (n=10, 58.8%) or hospital stroke units (n=7, 41.2%), with an additional job at an outpatient clinic in 2 (11.8%) cases. Most of the participants had a professional work experience of 2-5 years (n=5, 29.4%) or 5-10 years (n=5, 29.4%), fewer participants indicated 0-2 years (n=4, 23.5%) and more than 10 years (n=2, 11.8%) of work experience, and 1 (5.9%) participant did not specify any work experience. The majority of participants provided inpatient rehabilitation services (n=13, 76.5%), some provided day-hospital (n=8, 47.1%) and outpatient services (n=9, 52.9%), a few were involved in home-based rehabilitation services (n=4, 23.5%), and 2 (11.8%) of the participants did not specify any type of rehabilitation service they provide. Detailed information about the study participants is provided in Table 1. Most of the participants (n=15, 88.25%), except for 2 (11.8%) who did not provide feedback, felt that they were able to express their views and opinions during the discussion. Most of the participants who provided feedback (n=13, 86.7%) stated that they did not have any technical problems that disturbed their participation in the discussion. In addition, 1 (5.9%) of the participants had internet connection problems and 1 (5.9%) was disturbed by someone outside of the focus group, which hindered their ability to participate.

**Table 1:** Basic characteristics of study participants (N=17).

ID	Profession	Institution	Time working in stroke rehabilitation (years)	Type of rehabilitation service			
				Outpatient	Day hospital	Inpatient	Home based
1.1	Physiotherapist	Hospital	2-5	Yes	Yes	Yes	No
1.2	Occupational therapist	Hospital	<10	No	No	Yes	Yes
1.3	Speech and language therapist	Hospital	5-10	No	No	Yes	No
1.4	Physiotherapist	Hospital	5-10	No	No	Yes	No
1.5	Physiotherapist	Hospital	2 - 5	No	No	Yes	No
1.6	Physical medicine and rehabilitation physician	Hospital	0 - 2	No	No	No	No
2.1	Occupational therapist	Rehabilitation center	5-10	Yes	Yes	Yes	No
2.2	Occupational therapist	Rehabilitation center	0-2	Yes	Yes	Yes	Yes
2.3	Speech and language therapist	Rehabilitation center	0-2	No	Yes	Yes	No
2.4	Physiotherapist	Rehabilitation center	2-5	Yes	Yes	No	No
2.5	Occupational therapist	Rehabilitation center	5-10	Yes	Yes	Yes	No
3.1	Occupational therapist	Hospital	5-10	No	No	Yes	Yes
3.2	Occupational therapist	Outpatient clinic	2-5	Yes	No	Yes	No
3.3	Physiotherapist	Rehabilitation center	2-5	Yes	Yes	Yes	No
3.4	Occupational therapist	Rehabilitation center	<10	Yes	Yes	Yes	No
3.5	Physiotherapist	Rehabilitation center	0-2	Yes	Yes	Yes	No
3.6	Physiotherapist	Outpatient clinic	5-10	Yes	No	No	Yes

### Focus Groups

As outlined in Textbox 1 and Tables 2-4, 4 main groups of themes were identified: (1) the clinician’s views on using Vigo as a home-based rehabilitation system, (2) patient-related circumstances facilitating and limiting the use of Vigo, (3) Vigo’s functionality and use process (program creation, individual use, remote support), and (4) complementary and alternative Vigo use perspectives. The last 3 themes were further divided into 10 subthemes, and 2 of those subthemes had 2 sub-subthemes each.

**Coding framework matrix for theme 1 (“clinician’s views on using Vigo as a home-based rehabilitation system”).**

**Strengths**

- The use of Vigo potentially can partially compensate for the problems related to the availability of rehabilitation services and the lack of specialists.
- Reduce patient costs for rehabilitation, promote patient participation in rehabilitation activities at home, and reduce the involvement of specialists in the home environment.
- The goal of the rehabilitation process is to solve the patient’s problems through therapeutic activities rather than the patient’s involvement in activities.

**Limitation**

- Effective and targeted use of Vigo currently may only be possible for a small proportion of patients with stroke.

**Suggestions**

- In the development of Vigo, it could be important to determine the patients’ selection process and criteria so that the application of the method would be targeted and effective.
- A specialist could recommend the use of Vigo when they are sure that this method will be appropriate and effective for the target population.
- Involvement of a specialist with expertise in neurorehabilitation in further product development and research is required to prove Vigo’s effectiveness, appropriateness, and usefulness for rehabilitation purposes.

**Table 2:** Coding framework matrix for theme 2 (“patient-related circumstances facilitating and limiting the use of Vigo”).

Subtheme	Description
Personal factors	Strength: Previous rehabilitation experience can add to motivation and knowledge. Limitations: Age. Skills and habits of using smart devices. Lack of motivation for active involvement in therapy. Language skills. Health literacy.
Level of functioning	Limitations: The level of independence in carrying out activities is a potential barrier to being able to use Vigo. Impairment of structures and movement functions. Patients with cognitive, sensory, and mood disorders would not be able to use the app independently. Suggestions: Unsupervised use could be dangerous for patients with the risk of falls. A modified level of independence of functioning is most appropriate for the target audience. The use of the app is limited due to the need to read and understand the text.
Environmental factors	Limitations: Home environment and availability of amenities. Access to the internet and quality of the connection significantly affect user experience. Suggestions: Involvement of support persons for patients with greater functional impairment broadens the range of potential users. Involvement of specialists to assess the patient’s home and adjust the home environment. Possibility to download the content and use it without an internet connection.

**Table 3:** Coding framework matrix for theme 3 (“Vigo’s functionality and use process [program creation, individual use, remote support]”).

Subtheme: sub-subtheme	Description
The process of creating an individual therapy program: assessment of the patient’s situation	Suggestions: To create a rehabilitation program that meets the patient’s needs and abilities, specialist evaluation of the patient is required. Carrying out intermediate evaluations, assessing the dynamics of the patient’s condition, checking the compliance of tasks, and adapting the individual program to the current needs of the patient are equally important. An interim assessment could be conducted with the video call function built into the app.
The process of creating an individual therapy program: options of creating an individual therapy program	Limitations: Specialists are not clear on how the assessment of a patient’s functioning impacts the process of creating an individual program and its results. After getting acquainted with the demo version of the app, specialists have concluded that the selection of available exercises and tasks limits the realization of possible rehabilitation goals and is suitable for patients with mild impairment. Bilateral movement exercises, supine position, stable posture, and exercises for voice and dysphagia management are not included. Combining exercises into thematic modules, which are not modifiable, limits the possibility of adapting the program to the patient’s individual abilities and needs. Suggestion: A practical way to test the patient’s ability and motivation to use the device is to give it a trial run.
The process of executing the individual therapy program: information and communication in text format	Limitations: The communication of the digital assistant with the patient in the form of short message correspondence and the large volume of textual information place additional demands on the patient’s abilities and motivation to use Vigo. Patients with left hemisphere damage, confusion, and visual or perceptual impairment may have difficulties reading and keeping up with the information. There is no option to review the conversation on previous topics of conversation. Suggestion: Specialists suggest replacing text message correspondence with voice communication using multimedia content instead of text to inform the patient. Simplification of information and instructions, as well of adaptation of the app interface, would provide the possibility to adapt Vigo for use for patients with different abilities and needs.
The process of executing the individual therapy program: instructions and exercises in video format	Limitations: Specialists believe that the patient will not be able to perform the activity correctly without the supervision of a specialist. The demonstrations of exercises do not illustrate how they would be performed by a person with mobility limitations. Therefore, the perception of the exercises can be difficult for patients. There is no option to adjust the speed of the exercise demonstration, which is not appropriate for all patients. If a patient cannot perform or keep up with the exercise video, it might have a negative effect on the patient’s confidence and motivation. The patient must perform the exercises within several minutes without motivational stimuli and warning of the remaining time. The patient must be given the option of being able to choose to skip, modify, or stop the planned activity if they get bored or there is a change in feeling.
Remote support and related considerations	Strength: Remote patient counseling could be suitable for solving technical issues related to the app, but adjustment of exercises could require on-site consultation. Limitation: Specialists believe that remote assessment cannot be as high quality as meeting with a specialist in person. Suggestions: Remote support could be suitable for patients without cognitive impairment and with a milder course of stroke. The specialist should be in regular contact with the patient to perform an assessment and monitor changes in the patient’s condition.

**Table 4:** Coding framework matrix for theme 4 (“complementary and alternative Vigo use perspectives”).

Subtheme: sub-subtheme	Description
Application in the context of existing rehabilitation services	Strength: The use of Vigo as part of home rehabilitation services would allow the specialist to use the device in a targeted way to ensure the patient’s therapy needs, reducing the number of contact hours or increasing the intensity of therapy due to the patient’s independent involvement in the process. Suggestions: The use of Vigo in the home environment should be connected to home rehabilitation services. The specialists’ role would be to adjust the program, train the patient and caregiver to use the app, and monitor the therapy process.
Ensuring continuity of care	Strengths: Vigo could help ensure the continuity of rehabilitation specialists by giving recommendations on continuing the therapy process at home. Compared to printed recommendation materials, multimedia content can more transparently explain the activities to be performed, promote the patient’s motivation to engage in therapy, and cannot be damaged. Suggestions: By supplementing Vigo with information about the prevention of stroke complications and the availability of various support services, it would be possible to provide timely information to the patient when there is no immediate possibility to receive rehabilitation services. Consolidating the recommendations into 1 resource could make them easier to access and less likely to be lost. It is necessary to supplement Vigo’s content and functions so that it becomes a tool that professionals can use at work
Monitoring of therapy	Suggestions: Collecting data on the patient’s compliance with therapy could help assess the patient’s performance and progress, determine the presence of problems, and determine the need for support. Vigo has the potential to provide the patient with feedback after they have received rehabilitation services, which could imply new opportunities for research, service improvement, and coordination. It would be beneficial to add a medication schedule, provide an opportunity to film the patient performing tasks for use in the app, and broaden the daily schedule plan.
Support persons as primary users	Suggestions: Depending on the required level of assistance, relatives could be the ones who would assist with daily activities or provide the necessary care to prevent the risks of complications with the help of Vigo. Creating a section of the app for support persons as primary users would allow Vigo to be also usefully for patients with major functional impairment. It is necessary to inform the patient’s relatives about the possibility of receiving the necessary psychoemotional or social support services for themselves.

#### Theme 1: Clinician’s Views on Using Vigo as a Home-Based Rehabilitation System

When researching the opinions of specialists about Vigo, it was assumed that the main way to use the program was for the patient to use it independently (not including technical support, if needed) and the involvement of professionals during the development of the individualized program and interim assessment. Results from the discussions conducted showed that the relevance and potential benefits of using Vigo are as follows:

- Availability of services: The use of Vigo as an alternative to conventional rehabilitation services could be relevant for a patient whose functional impairment is mild enough to not interfere with the use of the device and would be more convenient than receiving rehabilitation services at home or in an outpatient setting. It is assumed that the use of a digital assistant could potentially help partially solve the lack of human resources in the rehabilitation sector under the condition that the specialists will not be excluded from the process of using Vigo, and it would also be a way to increase the intensity of therapy.
- Cost reduction: The possibilities to reduce the involvement of specialists in rehabilitation at home, saving the time of the patient and their caregivers’ travel expenses to receive the necessary services, could reduce the total costs of ensuring the rehabilitation process.
- Promotion of the patient’s independent involvement in therapy: Specialists positively evaluate the idea of the possibility for the patient to be involved in the rehabilitation process at home. Participants in the discussions expressed concerns that the technology without the involvement of a specialist could provide an opportunity to fully perform tasks. The daily individual therapy plan and the possibility to perform exercises along with the video demonstration could promote the patient’s compliance with the therapy. Better activity of the patient at home could lead to greater improvements in their functioning, which would reduce the amount of necessary care or assistance and the burden of support persons.

Participants of the focus group discussions identified the appropriateness of using Vigo for the rehabilitation process, indicating that it is necessary to pay attention to the purposefulness and usefulness of the product:

Rehabilitation activities do not take place to provide the patient with the opportunity to “do something.”

The adaption of the new technology must be aimed at improving the patient’s functional status at some point of using the program, and the effectiveness must be proven. Specialists believe that there should be a clearly defined target population and selection criteria and that only a small proportion of patients with stroke will be able to use Vigo at home for rehabilitation interventions. Patients with the potential for independent and purposeful use of a digital assistant must be motivated, cooperative, and critical of their condition. They cannot have severe cognitive impairment, neglect, aphasia, and sensory and motor impairments that would significantly limit their ability to use a smart device. In addition, selection criteria should be sensitive to all aspects of functioning, and therapy should be targeted and effective. Therefore, specialists from the field of neurorehabilitation should be involved in the definition and application of the criteria. One participant noted that they would not risk their professional reputation by recommending a therapy method that they were unsure of. It is suggested that further development of Vigo could include expert discussions on needs, iterative development of new features, and content with feedback from experts on the results of the practical application of the program.

#### Theme 2: Patient-Related Circumstances Facilitating and Limiting the Use of Vigo

According to the results of the study, the following factors were identified as facilitators of or barriers to the use of Vigo for patients: personal factors, level of functioning, and environmental factors.

##### Personal Factors

Personal factors, such as age, skills in using smart devices, motivation for active involvement in therapy, language skills, previous rehabilitation experience, and health literacy, were mentioned. One physiotherapist shared the experience of using Vigo with two patients, one of whom was 90 years old and the other was middle-aged. The elderly patient could not keep up with the text message correspondence with the digital assistant and had difficulty understanding how the communication was happening with a chatbot, not a real person. However, for the younger patient, “Everything was too slow.”

Another factor mentioned multiple times was motivation for active involvement in therapy. Many perceived rehabilitation as a passive process, gladly receiving a massage or other passive procedures.

A large part of those who request recommendations for doing exercises at home do not follow them.

Vigo could not be used for patients whose language of communication is not Latvian. One of the specialists mentioned that in their institution, “99% are Russian-speaking patients.” The use of Vigo might be easier for a patient who has already received rehabilitation and has the knowledge of how to perform the exercises. Performing tasks independently at home could then be done correctly if there is a limited opportunity to receive rehabilitation services. The health literacy of the patient and their support persons may affect their ability to participate in the therapy process in an informed manner. The way of providing information should correspond to the patient’s ability to perceive information, and it can also affect participation in the therapy process. Educational information should be presented in a simple way.

##### Level of Functioning

The functional status of the patient affects the use of Vigo, depending on the stage of rehabilitation, the level of independence of the patient, disorders of body structure and functions, the presence of risk of falls, and cognitive, sensory, and mood disorders. Specialists recognized that a patient’s functional status and not stage of rehabilitation of the disease will determine the patient’s abilities. The modified independence level of functioning refers to the patient’s ability to perform daily activities with necessary adjustments independently, which would also be the most appropriate target audience for Vigo use. Unsupervised exercise could be dangerous for patients with increased risk of falls. The limitations in patient functioning that were most frequently cited as an absolute or potential barrier to a patient’s ability to use Vigo were related to patients’ sensory and cognitive abilities. A patient who is uncritical of their condition or has neglected their paretic side will not be able to perform exercises without a specialist or caregiver. One specialist noted that none of the patients with stroke they worked with during the week would be able to use Vigo independently due to cognitive impairment. The use of Vigo is limited by the need to read and understand text to interact with the digital assistant. This could be difficult for patients with vision problems or cognitive impairment associated with damage to the left hemisphere of the brain.

##### Environmental Factors

The home environment and the involvement of caregivers were mentioned as important environmental factors. Participants mentioned that the involvement of specialists would be necessary to assess and adjust the home environment, when necessary:

Sometimes the patient doesn't even have a chair at home to sit down to do the exercises.

The availability of the internet and the quality of the connection can significantly affect the user experience or make the patient stop using the digital assistant if they must wait “for some spinning circle” while doing exercises. It was recommended to offer the possibility to download Vigo content to the device so that its user experience does not depend on the quality of the internet connection. By involving relatives in assisting in the use of the device for patients with greater functional impairment, the device could be used for a larger number of patients.

#### Theme 3: Vigo’s Functionality and Use Process (Program Creation, Individual Use, Remote Support)

Based on our results, the aspects of using Vigo can be divided into 3 subtopics: the process of creating individual therapy, the process of its execution, and remote support and related considerations.

##### Process of Creating an Individual Therapy Program

The process of creating an individual therapy program and related considerations include the assessment of the patient and adapting an individual therapy program, which must be carried out by a specialist in the field of neurorehabilitation to create a rehabilitation program that matches the patient’s needs and abilities. Without understanding the medical perspective of the problem, loved ones may think that the stroke made the person lazy:

Yeah, they think it's the willpower that's missing. He just doesn't want to get out of those diapers, or he doesn't want to talk.

For the successful application of Vigo, it would be equally important to meet with the patient again to make an interim assessment. The specialist should evaluate the dynamics of the patient’s condition, check that the patient performs the tasks correctly, and check that the created program is appropriate and adjust it, if needed. Specialists assumed that the partial assessment of the situation could be implemented using the video call function built into the app.

Specialists noted that the best way to check the patient’s ability to use the device is to use it for a few days under the supervision of a specialist. This would also allow the testing of the patient’s motivation and ability to use a digital device. Several specialists have concluded that they found exercises only for patients with mild functional impairment. Exercises with bilateral movements and a supine position and exercises for voice development and dysphagia management are not included. One occupational therapist noted that attention should be paid to assuming a safe and stable posture with which activities should be started. The specialist’s ability to customize the exercise program is limited by the fact that the exercises in the app are grouped into thematic modules, which cannot be changed by excluding an exercise from them if the patient cannot perform it. It is necessary to be able to adjust the speed of exercise demonstration, determine the number of repetitions, and modify the intensity according to the needs of the patient.

##### Process of Executing the Individual Therapy Program

Considerations of the individual rehabilitation program implementation process are mainly related to the text format of the content, as well as instructions and exercises in video format. One of the specialists noted that without cognitive impairment, they had difficulty reading the many explanatory text messages. A patient with confusion and visual or perceptual impairment, especially with left hemisphere damage, may have difficulty reading and understanding the educational information or instructions. Another disadvantage of the chat correspondence format is that previously provided information disappears and the patient cannot review the topics of previous informational conversations.

The educational information about the home environment is very long, so long that when you read it you forget what was written before.

However, “The information given before disappears.” By selecting specific topics that would be relevant individually for the patient, for example, basing their selection on screening questions, the amount of information provided could be reduced. Possible solutions for overcoming obstacles related to the perception of information would be to play the text in voice, to replace the educational text with a short video, to assist loved ones in reading the text, to simplify the instructions and content, and to adapt the visual interface.

Regarding the exercise process, specialists were concerned that without specialist supervision, the patient may not be able to perform the tasks correctly, even if they are suitable for them.

The exercises are demonstrated at the same speed for both sides of the body, which makes it difficult to understand which limb is paretic.

It was also noted that the initial familiarization information for the patient is “horribly long” and overwhelming and that during an exercise block that might last 10 minutes, the patient has no option to stop the activity if they are tired or bored; during the exercise process, the patient is not encouraged or warned about the remaining exercise time. One of the speech and language therapists noted that the exercises to improve the function of the structures involved in articulation and mimicry were demonstrated at a faster rate than the patient would normally be able to follow.

The application is difficult at the moment.

If the patient is motivated but cannot perform the tasks, then this could make the patient feel bad about themselves and stop using the device. Participants indicated that the patient could be bothered by not being able to skip exercises, follow video instructions for exercises they already know, change the order of exercises, and adjust the speed of the exercise demonstration and would need the ability to modify the exercise program, depending on how they are feeling.

##### Remote Support and Related Considerations

The participants believed that remote assessment of a patient cannot be as high quality as in-person assessment. However, the condition of patients is prone to change, so it is necessary to communicate with specialists at least remotely. Providing remote support requires additional resources and targeted work organization, as specialists working in inpatient or outpatient institutions are not able to respond to patients’ video call requests because they do not have time. For technical support, when a patient needs an explanation about the functions or use of the app, a phone conversation could also be sufficient.

#### Theme 4: Complementary and Alternative Vigo Use Perspectives

##### Application in the Context of Existing Rehabilitation Services

Specialists believed that Vigo could be used not as an independent rehabilitation method that tries to replace a functional specialist but as an aid for relatives or an additional tool for rehabilitation specialists. The use of Vigo should be connected with rehabilitation services at home, in the framework of which the specialist’s task would be to adjust the device, educate the patient and their relatives about its use, and regularly monitor the therapy process. It could also help compensate for the lack of human resources by reducing the number of specialist contact hours required.

##### Ensuring Continuity of Care

The use of smart devices could be a great tool and the next step in the use of technology in rehabilitation. Vigo could help ensure continuity of service after discharge from the hospital and prior to and after discharge from inpatient rehabilitation. By adding information about bedsores, other possible complications, and where to find medical assistance, the doctor could use this app to ensure timely and high-quality patient information. In addition, instructions in video format have an advantage over printed visuals, as they can provide a better idea of the required movements, as well as promote patient motivation.

##### Monitoring of Therapy

Specialists concluded that Vigo’s content should be supplemented and suggested creating an opportunity for the specialist to film the patient performing tasks so that the patient can use these materials in the app. In addition, the app should provide an opportunity to enter the necessary medication schedule so that the patient can receive reminders when the medication needs to be taken and an opportunity to create a wider daily plan for daily activities with their performance times. Vigo has the potential to provide therapy monitoring both by following the patient’s response during therapy and by evaluating the results (follow-up).

##### Support Persons as Primary Users

It is difficult for specialists to imagine that a patient in the acute phase of the disease could practice dressing, washing, and other activities without the support of caregivers. It is possible that a person who can use a smart device will also be able to perform the mentioned daily activities. Therefore, the participants suggested that the patients’ relatives could be the target users. This would be a suitable use for patients with severe stroke who require moderate assistance or patients with cognitive impairment. Timely involvement of relatives could help ensure that a patient with a more severe course of stroke would not end up in a rehabilitation service with additional complications, because they would have simply slept at home without receiving the necessary care. The loved ones themselves may need psychoemotional support to overcome the effects of their relative’s illness and caregiving experience.

## Discussion

### Principal Findings

This study explored health care professionals’ opinions on the use of the digital assistant Vigo for patients recovering from stroke. Qualitative research on the adaption of stroke rehabilitation technologies shows that stakeholders have identified that key points, such as access to technologies, ease of use, supported self-management, evidence of effectiveness, value for money, knowledgeable staff, and feedback, are important with regard to successful adaption of the use of technologies in stroke rehabilitation [10]. Results of this study show that health care professionals have similar opinions about aspects important for meaningful use of the digital assistant Vigo. Findings of this study are also consistent with our previous research on patient perspectives on the use of the digital assistant Vigo, where the main results showed that patients have a positive attitude toward the use of technologies at home, and highlighted the importance of the simplicity of app design, flexibility of content, and benefits on the individual level. Some common points from patients and professionals were about the amount of text in the chatbot, complexity of the information, variety and difficulty of exercises, and practical use in the home environment [20]. Specialists suggested that it would be less demanding for the patient if there was a voiceover option and if the information was illustrated and simplified. It would also be beneficial if the person demonstrating exercises would be someone who has had a stroke and if the user would have the ability to adjust the speed and repetition of the exercises.

The literature shows that the user-centered approach is required to meet the requirements of intended users (health care professionals, patients, and their caregivers) [25-27]. Although there are mixed findings about the opportunities and benefits of home-based technologies, some studies show that home-based rehabilitation technologies offer interventions that are equivalent to conventional interventions [11]. Specialists agree that the digital assistant could partly compensate the shortage of specialists and availability of rehabilitation services, reduce costs, and promote patient participation. Both patients and professionals have shown acceptance of and satisfaction with telerehabilitation interventions, but there are still many barriers [28]. One of the themes that emerged in all the discussions was assessment. Rehabilitation specialists expressed that an important step for development of the app is defining the inclusion process and criteria for patients to use the program purposefully and effectively, with the main concern being that, currently, there is a small group of patients with stroke that would benefit from the use of the tool in the home environment.

Considering the aspects mentioned about defining inclusion criteria and assessment, the process of developing an individual program requires direct involvement of the therapist. The only way the health care professional can be certain that the program is appropriate is to test the program together with the patient. There needs to be an assessment of the patient’s functional status, not only at the beginning, but also in the interim, to check whether any adjustments are required. Telerehabilitation services through video calls could be applicable for patients without cognitive impairment and mild functional impairment, as well as addressing technical issues. Specialists believe that remote functional assessment of the patient will not be as accurate as in-person assessment.

Our results also add to the research on the barriers to and opportunities of assistive technology transitions into stroke rehabilitation where the key barriers are knowledge, education, awareness, and access [29,30]. Specialists mentioned that the following patient-related conditions are important for the use of the app: patients’ personal factors, functional status, and environmental factors. All the mentioned factors can be barriers, but if addressed correctly, they can become facilitators. Training and good knowledge of professionals, patients, and their caregivers can potentially eliminate some of the barriers regarding uncertainty about the use of the app in the home environment. Additionally, a secure and strong internet connection plays the most important role, because issues with a poor internet connection can lead to poor quality of videos and a longer waiting time for loading content, thus negatively affecting motivation for regular use of the program [31].

ICT has the potential to effectively provide home-based telerehabilitation services, improve patient education, and provide a means of interaction [32]. Reduction in the availability of poststroke rehabilitation caused by SARS-CoV-2 has serious consequences, indicating that telerehabilitation could be 1 of the solutions as an alternative for therapeutic interventions [28,33]. Health care professionals proposed that the app could also be not only a digital assistant but also a tool to reduce the number of contact hours or increase the intensity of home-based rehabilitation, provide an alternative format to traditional written recommendations for continuing rehabilitation at home, provide feedback, and serve as a guide for the caregivers of patients with stroke.

In summary, health care professionals highlighted the important aspects related to the process of using Vigo in relation to the functionality of the app and patient conditions. The possible barriers and facilitators described indicate that the perspectives of all end users (patients, caregivers, health care professionals) need to be considered in the process of developing a home-based stroke rehabilitation tool. Results of this study shows the complexity of ICT use in the context of stroke rehabilitation. These results outline important key points that developers need to consider in the process of designing home-based e-rehabilitation tools for patients with stroke.

Limitations

There is a lot of quantitative research on the efficacy of different digital tools and technologies used in stroke rehabilitation and qualitative data about patient experience, but there is a lack of information about health care specialists’ opinions about specific program relevance to the target population [11,16,34]. Most of the specialists working in rehabilitation in Latvia are females; thus, there were no male participants in the study. The digital assistant Vigo is a relatively new application, and specialists have had limited opportunities to test the program with patients with stroke. Although each participant in the study was provided with a description of the program and an opportunity to test it, only a few had experience with adjusting the content of the app according to patients’ individual needs and functional status. Therefore, specialists had a lot of suggestions and questions about the process of creating an individual program and patients’ ability to use it independently at home. Developers need to consider providing more possibilities for specialists to learn more detailed information about the app, its content, and practical use. Some of the participants had a lot of practical considerations about implementing the app in practice that indicates the need for specific training.

### Conclusion

Overall, health care professionals expressed a positive attitude toward the usability of the Vigo app, but the app is still a work in progress to show any improvements in patients’ functional outcomes. The digital assistant has the potential to partly compensate for the problems of the availability of rehabilitation services and lack of specialists if the program is adjusted appropriately and the patients’ functional status allows the use of the app independently. Developers’ biggest challenge is to create an app that is adjustable to each patient’s individual factors and abilities. It is important that the content and use of the app be coherent with the aim and description defined by the developers. Otherwise, there is a risk of misunderstanding its practical use, not understanding the need for integration in practice, and misuse of the app. To use the app not only to engage the patient in some sort of activities but also to have a therapeutic effect, close involvement of rehabilitation specialists is needed in the process of app development and research.

## Appendix

Multimedia Appendix 1 Focus group transcript 1.

Multimedia Appendix 2 Focus group transcript 2.

Multimedia Appendix 3 Focus group transcript 3.

Multimedia Appendix 4 Interview guide for the focus groups.

## Abbreviations

ICT: information and communication technologies

## Data Availability

Anonymized focus group transcripts in the original language are available as Multimedia Appendices 1-3, and the interview guide for the focus groups is available as Multimedia Appendix 4.
