# Supplementary material for: Use of the Digital Assistant Vigo in the Home Environment for Stroke Recovery: Focus Group Discussion With Specialists Working in Neurorehabilitation
Source: JMIR Rehabil Assist Technol. 2023 Apr 14;10:e44285. doi: 10.2196/44285 (PMC10148207; doi:10.2196/44285)
Supplement: Multimedia Appendix 1 [file rehab_v10i1e44285_app1.pdf]

1 [0:00:00.0] Fokusgrupa Nr.1 transkripts.

2 [0:00:29.0] Mod: Es piedāvāju sākt ar nelielu iepazīšanos. Ļoti īsi, netērējot laiku. Lūdzu, pasakiet kādu rehabilitācijas profesiju Jūs pārstāvat un kāda ir Jūsu darba pieredze saistībā ar insulta pacientu rehabilitāciju? Nu, es nezinu, varam sākt no vienas puses uz nākamo pusi, pēc kārtas.

3 [0:00:48.0] Dal1: Labdien! Kolēģi mani jau zin, tāpēc iepazīstināšu Jūs ar sevi. Esmu Anna, esmu sertificēta fizioterapeite, strādāju insulta vienībā nu jau 7 gadus. Strādāju akūtajā rehabilitācijā, strādāju insulta vienībā un ir arī pieredze mājas rehabilitācijā. Mājas rehabilitācijā esmu strādājusi 3 gadus.

4 [0:01:15.0] Mod: Paldies!

5 [0:01:17.0] Dal2: Es esmu Elīna, strādāju insultu nodaļā, insulta vienībā jau 10 gadus. Pirms tam man ir 5 gadu pieredze subakūtajā posmā un arī tā pat kā Dal1 esmu strādājusi mājas aprūpē, mājas rehabilitācijā, arī līdzīgi, kādi 3 gadi. Nu tas laikam arī viss.

6 [0:01:42.0] Mod: Paldies!

7 Dal3: Labdien, mani sauc Ilze, esmu fizioterapeite. Ar insulta vienību, ar akūtiem insulta pacientiem strādāju pēdējos 3 gadus. Man ir arī pieredze strādājot ar insultiem paralēli ar citām saslimšanām, ņemot vērā, ka slimnīca ir plaša profila slimnīca. Tā kā, un man bija īsa, īsu periodu pieredze strādāt arī mājas aprūpē, nu ar insulta pacientiem.

8 [0:02:15.0] Mod: Paldies!

9 [0:02:17.0] Dal4: Es esmu Inese. Esmu rehabilitācijas ārsts - rezidents, 3.gads, no kuriem gandrīz 2 pavadu insulta vienībā. Nu labi, akūtajā, akūtā insulta nodaļā, pēc tam rotējot, respektīvi tie ir jau subakūtie fāzes insulta pacientiem.

10 [0:02:38.0] Mod: Paldies!

11 [0:02:40.0] Dal5: Es esmu audiologopēde Alise un insulta nodaļā, insulta vienībā strādāju 5 gadus, tā laikam sanāk. Un, paralēli esmu strādājusi arī ar citām pacientu grupām, pārsvarā tā ir ķirurģiskā vai (nesaprotams) terapijas bloka pacienti ar pneimonijam un tāda veida saslimšanām. Un, es nepateikšu cik gadus, bet man ir pieredze arī ar pacientiem mājas rehabilitācijā, kas ir pamata insulta pacienti

12 [0:03:09.0] Mod: Paldies!

13 [0:03:10.0] Dal6: Diga, es esmu fizioterapeits un neiroloģijas joma laikam, nu tai skaitā insulti, varbūt ne tik izteikti cik kolēģiem, bet 5 gadi, kopumā es teiktu. 6, bišķīt vēl bija pirms tam mājas aprūpe, tur pamatā bija insulta pacienti. Jā, kaut kā tā.

14 [0:03:33.0] Mod: Paldies! Nu labi, tad pirmais jautājums būtu - Kā jūs komentētu plašsaziņas līdzekļos pieejamo informāciju par digitālo asistenu "Vigo"?

15 [0:03:53.0] Dal2: (nesaprotams) Jo, es izteikšos (nesaprotams). Es biju, nu tā kā, es, teiksim, es zinu, ka viņš tāds ir, bet es no profesionālā viedokļa zinu tagad, jo kad sāka viņu veidot un tā, un tas Vigo asociējās daudziem insulta pacientiem ar to biedrību kas viņiem ir. Ar ko man arī bija saskare, bet teiksim tā, ka man viņš pēkšņi kaut kur man parādās, kaut kur uzpled vai vienkārši no pacienta, tad neesmu tādu manījusi.

16 [0:04:22.0] Dal1: Man arī teiksim, es esmu dzirdējusi tikai garām ejot par šādu programmu insulta pacientiem, bet sastāvu redzēju, reāli varēju iepazīties pirmo reizi izmēģinot.

17 [0:04:41.0] Dal4: Nu, es to Vigo arī zinu tikai tāpēc, ka es visu laiku esmu kaut kur darbā un viņš tāds eksistē. Man viņu facebooks ieteica, pēc tam, kad es kolēģiem par to runāju, esmu ar telefonu rokās, bet ārpus darba man nekādas informācijas.

18 [0:04:57.0] Dal5: Man laikam ir līdzīgi, ir lasīti raksti kaut kur mēdijos par to, ka vispār tāds ir izstrādāts, tāda programmatūra, ka viņa būs arī pieejama pacientiem mājas vidē, nu arī kā valsts apmaksāts pakalpojums, bet sīkāka, laikam tāda detalizētāka iespēja iepazīties, līdzīgi kā kolēģiem bijusi, tāpēc, ka mēs šajā jomā strādajam. Zinu arī to, ka ir facebook grupa, kas saucas man šķiet laikam "Dzīve pēc insulta" vai kaut kā tamlīdzīgi un tur ļoti, tā aktīvi tiek šī programma tiek pieminēta, ka viņa ir, ieteikums ir raisošs, jo var arī pacienti lietot. Tikai, dažkārt tā informācija arī ir tāda neprecīza, nu tā laikam to varētu teikt.

19 [0:05:40.0] Dal6: Es esmu ievērojusi linkedin, tas saistīts droši vien ar Kristapu Krafti un Jāni Slēžiņu, viņi piedalījušies kaut kādāds konferencēs, semināros vai kaut kādās sacensībās, kur viņi piedalās ar Vigo. Tad tādā veidā ir vēl informācija. Bet, tas arī tādā profesionālā laukā vairāk.

20 [0:05:58.0] Dal5: Nu īstenībā, arī tā publikācija viņiem bija. (Dal6 krata galvu, piekrīt) Tas arī tad vairāk profesionālos aspektos.

21 [0:06:09.0] Mod: Pirms diskusijas bija iespēja arī aplūkot viņu mājas lapu un facebook lapu, vai ne? Vai tur ir kaut kas komentējams?

22 [0:06:19.0] Dal1: Nav nekas komentējams.

23 [0:06:24.0] Mod: Labi, un kāds ir jūsu viedoklis par izglītojošās informācijas un vingrinājumu atbilstību insulta pacientu rehabilitācijas nolūkam Vigo asistentam?

24 [0:06:38.0] Dal1: Nu, šeit mēs tā līdz galam kārtīgi nesapratām šo te atlases kritērijus šiem pacientiem. Kādai tad mērķa grupai šis te Vigo ir takā, ka, kam viņu ieteiku. Vai ir kaut kādi atlases kritēriji?

25 [0:07:00.0] Mod: Tas arī ir jautājums par ko mēs diskutēsim, bet tieši runājot par pašu saturu, kādi tur vingrojumi norāditi, tajā lietotnes datu bāzē. Kā jūs vērtējat to kvalitāti, raksturu?

26 [0:07:17.0] Dal5: Tieši izglītojošie vai visi?

27 [0:07:19.0] Mod: Gan izglītojošā informācija, gan arī vingrojumi, kas ir tajā lietotnē. Vai tie atbilst ta kā insulta pacienta vajadzībām, vai kā jūs raksturotu šo saturu?

28 [0:07:32.0] Dal5: Es laikam varu pakomentēt no savas perspektīvas, kā audiologopēdam. Man radās tas jautājums vai tajā izmēģinājuma versijā, kas man bija iedota planšetē, tur bija skāriena lokalizācija, takā bojājuma lokalizācija, klīniskā diagnoze. līdz ar to mēs pieņēmām, ka tie vingrinājumi ir sagatavoti pacientiem pie labās puslodes bojājuma. Man tiktāl takā viss ir skaidrs un tur bija tie, piemēram, (nesaprotams) lūpu vingrinājumi, nu kas man nekādus īpašus jautājumus varbūt neradītu, viņi ir adekvāti, arī tas materiāls pats ir pietiekami kvalitatīvs. Tikai jautājums ir kā viņi tiek piemeklēti, tas ir viens. Vai tur ir iekļauta tāda kā atlases kārtība, vai tas cilvēks trenē mēli un lūpas, piemeram, ēšanas aktivitātē, vai arī tad, kad viņš trenē runāšanas aktivitāti, jo tad tās kustības varētu atšķirties. Tas ir viens. Tas lielākais jautājums, kas man principā rādās, vai vispār Vigo ir iestrādāts pacientam ar kreisās puslodes bojājumu, tā kā valodas rehabilitācijai? Jo tajā aprakstā es to arī neredzēju. Man šķiet, kad ja tā nav, tad tas varētu būt diezgan paliels ierobežojums, tapēc, ka viņu takā, kāds ir tas Vigo lietošanas mērķis? Es viņu vispār, vispār nevarētu rekomendēt pacientiem pie kreisās puslodes smadzeņu bojājuma. Tas bijas tas lielākais jautājums.

29 [0:08:51.0] Dal2: Nu, es arī esmu papētījusi un paspaidīju, tādā ziņā, ka, ja es būtu pacients, atklāti sakot, ņemot vērā, ka tas tomēr ir insulta pacients un jautājums vai viņš jau ir mājas rehabilitācijā, ļoti liela atšķirība vai viņš to planšeti saņem uzreiz pēc stacionāra, vai viņš to saņems pēc stacionāra rehabilitācijas. Jo, ja šis pacients nokļūst mājās uzreiz pēc stacionāra, viņš ir diezgan gan apjucis, viņš vēl nevar izprast īsti savu funkcionālo traucējumu un līdz ar to, teiksim, sekot līdzi un lasīt šo te izglītojošo sadaļu, varētu būt apgrūtinoši, manuprāt (Dal5 māj ar galvu) . Jo, viņi nespēj ilgi koncentrēties, viņiem vajag sakārtot brilles, viņiem iespējams no audiologopēda viedokļa nespēj to lasīto tekstu nemaz saprast, jā, tās instrukcijas izprast . It īpaši, ja tas ir kreisās puses traucējums,

kreisās puslodes, tad šī te instrukciju izpratne pazūd. Un līdz ar to, tad līdz ar to arī visu laiku kādam jābūt blakus, kas viņam to skaļi nolasa. Iespējams, ka varētu viens no ieteikumiem, lai, ja tas vispār ir iespējams, ka šajā planšetē ierunā arī, ierunāto virtuālo asistentu, kas runā balsī. Jo sekot līdzi tam pierakstam watsapp čatiņā, cilvēkiem jau teiksim, vecāka gada gājuma, iespējams būtu apgrūtinoši. Un, ja viņiem skaidri pasaka, un viņi bieži paliek viens pret viens ar sevi un savu traucējumu, viņi justos varbūt ne tik vientuļi, ja ar viņiem kāds runātu un tādā veidā dotu komandas. Jo, tomēr, valodas komandas tomēr uztver labāk nekā rakstītajā veidā. Tas ir par to. Par, šo te, saturu kā tādu. Saturs varētu būt, kā pareizi minēja Dal5, varētu skatīties atkarībā, kāds ir pusložu bojājums vai atkal, ja arī tas ir smadzenīšu bojājums ,tad šiem cilvēkiem atkal pievienot šo pašu runāšanas, ierunāšanas veidu. Viņiem mēdz būt dubultošanās, redzes traucējumi, izlasīt viņi vispār nevarēs, kas tajā planšetē viņiem tiek dots. Un līdz ar to vajadzētu biškīt pamodificēt šo sadaļu. Par, vēlreiz atgriežoties par saturu, atkal - atkarībā kāds pacients, kāda atlase, vai būs kas stāv viņam blakus vai nebūs, kas stāv viņam blakus. Tā patās, kā piemēru, mazgāšanās aktivitāte, rokas mazgā, tajā sadaļā. Es nesadzirdēju, bet, manuprāt, būtu ļoti svarīgi, kas ir primārais insulta pacientiem, viņi ieņem stabilu un drošu pozu. Jūsu piedāvatajā versijā pacients stāv kājās un mazgā rokas pie ļoti platas, skaistas izlietnes, kur ir vieta nolikt parētisko roku pēc tam. Tas bišķiņ ir atrauts no realitātes.Un, tur kur ir roku vingrinājumi, ir okay, vingrinājumi var būt dažādi modificējami un tādā gārā, kā vajag katram pacientam, bet es varbūt, lai būtu šiem pacientiem tuvāk, lai viņi izprastu vairāk, varbūt liktu īsta pacienta rokas. Kaut vai ar īstu vieglu parēzi, jo tur tik acīmredzami cilvēks ir vesels, ka tur ir grūti izsekot, kura ir tā parētiskā roka un šis vingrinājumu ātrums abām rokām ir vienāds. Insulta pacientam ši te domāšana, izdomāšana, izpildīšana notiek daudz lēnāk, manuprāt. Vajadzētu gan lēnāk, gan arī kaut kādā veidā to parēzi, vai nu labāk notēlot vai ņemt kaut kādu pacientu, lai ir īstāka situācija. Par ieteikumiem ēšanas ziņā, es neesmu uztura speciālists, neesmu ne endokrinologs, ne ārsts, bet tur es esmu ievērojusi, ka pacienti šausmīgi baidās no atkārtota insulta, viņi baidās. Viņi vispār baidās no šīs situācijas, ka viņi ir kļuvuši kaut nedaudz aprūpējami un tur bija viens tāds teksts, kas, ja es būtu pacients, nedod Dievs, mani nobiedētu. Tur ir kaut kā pieminēts, ka, aptuveni doma ir tāda, ka nepareiza ēšana izraisa atkārtotu insultu. Es vēl no tik skaļiem tekstiem insulta pacientiem atturētos, ja ka, tur ir minēts, ka jāed pareizi, lai izvairītos no atkārtota insulta vai pasargātu. Manuprāt, viņu situācijā, primārais tā pat ir medikamenti un tad pēc tam. Takā drusiņ pielāgot realitātei. Nevajag baidīt to pacientu.

30 [0:13:32.0] Dal1: Bišķiņ papildināšu, to situāciju varētu glābt tas, ka pārsvarā insulta pacientiem, viņu (nesaprotams) vājdzirdība (citi dalībnieki smejas, izsaka piebildes, nav saprotams)

31 Dal2: (nesaprotams), Viņam viss, viņš tagad tiešām divas reizes nedēļā ēdīs zivi, nu kaut kā, bišķiņ advencētāk vajadzētu. Tas tā laikam viss īsumā.

32 [0:14:07.0] Mod: Vai fizioterapeitiem būtu komentārs par vingrojumiem, kas ir lietotē atrodami?

33 [0:14:14.0] Dal1: Varētu pateikt savu viedokli, jā, man pieņemsim tie vingrojumi, viss jau ir daudz maz, viss tur kārtībā tur ar viņiem. Bet tas, ko es varu pielikt no savas puses. Ejot uz šim mājas vizītēm, tad, pirms ko jau pirms tam minēja kolēģe, ka viss ir ļoti skaisti parādīts, bet dažkārt, mums, kā fizioterapeitiem, nākas pielāgot vingrinājumus pie šīs mājas vides. Dažkārt tur pat nav krēsls, kur pacientam apsēsties, lai šos vingrinājumus pildītu. Es uzskatu, ka šim te reālajam asistentam, fizioterapeitam, tomēr ir neatsverama loma šajā subakūtajā mājas rehabilitācijā. Bet baidos, ka programma, nu, tur, diezgan čābīgi būtu.

34 [0:15:18.0] Dal4: Ņemot vērā, ka es vēl strādāju ar ne medīcinas studentiem kā pasniedzējs. Takā, citas jomas speciālisti. Tas, ko tur var novērot, ir tas, ka vidējam cilvēkam, kurš nav piesaistīts medicīnai, vārds "insults" neko neizsaka, vārds "veselīgs uzturs" neko neizsaka un tā veselības pratība ir ļoti kritiskā līmenī. Tur varbūt jāapskata, jāapdomā, cik ļoti vienkāršotā un tiešām sagremojamā veidā tiek skaidrots, kas vispār ir noticis, jo atrauti no slimnīcas, tajās nodarbības, kur stāstu kas ir insults, teiksim, psihologam. Viņi man saka, ka insults ir tad, ja tu esi slinks, nu tur nāk ārā visādi brīnumi. Līdz ar to, ja mēs iedodam viņiem ļoti advencētu programmu, bet viņi tajā pašā būtībā vispār nesaprot kas ir noticis, tai sadaļai kas ir par izglītošanu, ir jābūt tādai (nesaprotams). Diemžēl, arī augstākā izglītība neliek saprast kas vispār ir noticis. Man pacientu radinieki ir zvaījuši un teikuši: "Mans tētis vairs nav mazais bērns. Viņš jau tagad beidzis slinkot, kāps ārā no pamperiem". Šis cilvēks vispār nesaprot ne par ko ir runa, ne kāpēc tāda aplikācija ir vajadzīga, ne kāpēc mēs te vispār kaut ko runājam. Un arī ir jāļauj šim te, kas ir pirmārā, sekundārā un terciālā

profilakse insultam. Jo, ja aplikācija runā par uzturu jau brīdī, kad ir bijis notikums, tā jau pārvēršas par sekundāru profilaksi, kas tiešām jau ietver medikamentus, korektu diagnožu menidžmentu

35 [0:17:00.0] Dal2: (piebilst) Ārsta apmeklējumu

36 [0:17:03.0] Dal4: (turpina) Ārsta apmeklējumu, vispār vēršanaš pie, ārsta vizītes. Ārsts, kurš vēl ar pacientu komunicē, jāspēj runāt kā ar mazu bērnu, jo tāds ir veselības pratības līmenis vidējam cilvēkam. Viņš ir zemāks par zāli. Un, ja aplikācija saka "veselīgs uzturs" un cilvēks neatšķir kas ir olbaltumviela un, ka tas ir tas pats proteīns un ogļhidrāts ir cukurs, tas pats, kas glikoze. Tad viņi vienkārši kaut kā tukšām acīm skatās un tas viss aiziet kaut kur. Un priekš tā radinieka, kurš to aplikāciju lieto, visa tā informācija ir tik pat tukša kā afātiskam pacientam stāstīt: "pacel rokas". Viņi visi kopā, nesaprot par ko ir stāsts. Es to saku tāpēc, ka caur mani iziet gan, ļoti daudz studentes, visas skatās tukšām acīm, bieži arī radinieki tiem pacientiem, kas atnāk pie mums. Viņi nesaprot par ko ir runa.

37 [0:17:54.0] Dal5: Viņi tiešām nesaprot.

38 [0:17:55.0] Dal4: Tiešām nesaprot.

39 [0:17:56.0] Dal5: Viņi tiešām nesaprot par ko ir runa. Kaut vai vienkāršie valodas uzdevumi, ko mēs pildam. Viņi skatās lielām acīm un saka, ka tas jau takā bērnudārzā. Principā, tur ir tas realais līmenis cilvēkiem.

40 [0:18:04.0] Dal4: Jā, viņi tiešām nesaprot par ko vispār ir runa un..

41 [0:18:09.0] Dal5: Jā, viņi domā, ka tas ir gribaspēks, kas apmēram pietrūkst. Viņš iekšā vienkārši negrib izkāpt no tiem pamperiem vai viņš negrib runāt.

42 [0:18:15.0] Dal4: Jā, arī visi šie depresīvie traucējumi.Viņiem jau jāsaņemās un vispār tai izglītojošanas sadaļai ir jābūt būtu tik jaudīgai, ka mes izglītotu 5 gadīgus bērnus un tā uz to attiecas, diemžēm, pēc vidusskolas.

43 [0:18:30.0] Dal2: Jā, un papildināšu par tiem, vēl depresīviem noskaņojumiem. Viņi jau arī mēdz veidoties organiski patiesībā, nevis tāpēc, ka cilvēks vienkārši ir nolaidis rokas, bet tāda ir viņu fizioloģija, kas bieži vien ietekmē. Un ar vārdu "saņemies" bieži vien pietrūkst, tad jāskatās varbūt vajag kādus medikamentus klāt. Un jā, es atkal klausoties Dal4 atkal dzirdu to, ka ir ļoti jāizvērtē kurā posmā, tāpec arī ir tas jautājums par kritērijiem. Pēc kādiem kritērijiem šie pacienti tiek atlasīti, kurā posmā šis pacients saņems šo planšeti. Kad viņš noteikti jau ir kaut ko izglītojies, kaut ko saņēmis, tad viņiem būs šī sapratne. Ja viņam iedod uzreiz pēc stacionāra, manuprāt, tas nebūtu. Vai arī, vēl viens variants, kas atkal lietojot planšeti, bet tas atkal ir tas pats cilvēku resurs. Vai šim pacientam ir iespēja caur to planšeti sazināties ar kādu no fizioterapeitiem, ar kādu kas veido šo planšeti. Jo, ja tur bišķiņ kaut ko ne tā, teiksim, mums gaiļezerā, kā RAKUS viesim, bija internets ļoti lēns un šī plašete visu laiku kārās un ļoti lēni gāja. Tas itkā ir interneta vaina, cik es sapratu, bet.. cilvēks vecāka gada gājuma, viņš neizprot. Un planšetei ir diezgan neērta turēšana, ja viņam ir parēze un ar vienu roku viņam viņa ir jātur, vai arī jaiegādajas viņam statīvs, lai viņš viņā lien. Tīri tādas praktiskas lietas. Šis produkts un tā doma ir ļoti jauka, bet vajadzētu vēl padomāt..

44 [0:20:04.0] Dal1: Nu jā, pacientam jābūt kaut cik funkcionāli neatkarīgam, lai viņš vispār šo te planšeti varētu lietot un es gribētu piemetināt to, ka ejot uz šīm mājas vizītēm. Manā nelielajā pieredzē, es varu pateikt to, ka 70-80% , kas pieejams, vispār mājās nav pieejams internets.

45 [0:20:24.0] Dal5: Jā, tas arī bija, par ko es domāju. Man liekas, ka tā tiešām bija interneta vaina. Man liekas, ka tie uzdevumi , viņi varbūt ir tajā (nesaprotams) brīdī, jā speciālistam varbūt ir vajadzīgs tas internets, bet man šķiet, kad tas cilvēks skatās viņus mājās, viņiem ir jābūt pieejamiem oflainā. (nesaprotams) Citādāk, ja viņam būtu jāgaida kāmēr kaut kāds tur rimbulīts griežās. Viņš jau tikmēr izslēgs ārā to planšeti. Labi, ja viņam vispār ir internets mājas.

46 [0:20:49.0] Dal1: Viņam noteikti, tam vecajam cilvēkam būs bail sabojāt šo viedierīci. Nedod Dievs,

vēl kāds viņam pēc tam pieprasīs kaut kādus naudas līdzekļus. Viņš viņu vienkārši noliks uz plauktiņa un viņu neaiztiks.

47 [0:21:04.0] Mod: Paldies. Nu, mēs jau sākām runāt par to kā to visu programmu pielāgot pacientiem. Kāds būtu, Jūsuprāt, veiksmīgākais veids kā pielāgot programmu atbilstoši pacienta vajadzībām?

48 [0:21:24.0] Dal2: Tur ir jābūt, ja tie ir, ja šim pacientam ir nepieciešami šie trīs speciālisti, tad arī ir jābūt visiem šiem trim speciālistiem pie šī pacienta. Viņiem ir jāredz, vienalga kurā posmā. Tad konkrēts speciālists pielāgos abus vingrinājumus vai kaut kādus savus ieteikumus, vai vēl kaut ko. Tas pats arī par to dozāciju, cik daudz un cik bieži. Vismaz zvana attālumā ir jābūt, jo pacienta stāvoklis ir mainīgs. Mēs nevaram iedot viņam uz nedēļu. Varam, gan jau ir kāda procentu grupa, kas varētu, kuriem varētu iedot uz nedēļu un pēc nedēļas viņus apmeklēt. Tad varbūt tur vajadzētu kaut kādus kritērijus atrunāt. Ja, jums tur, nezinu, spiediens ir tāds, tad tur ir tādi vingrinājumi. Ja jums ir tas, tad ir tas. Un tā pielāgošana notiek, lai, cik tas atvieglotu cilvēku resursu trūkumu šajā mājas rehabilitācijā, tiem speciālistiem tāpat ir jābūt piesaistītiem. Uz mazāku noslodzi, bet ir jābūt.

49 [0:22:23.0] Dal5: Es laikam varu papildināt no savas perspektīvas. Atkal, ja es kļūdos, tad droši varat labot. Bet es saku, ka tiešām tas mēles un lūpu modulis, kas ir šie artikulācijas vingrinājumi. Līdz ar to, no audiologopēda perspektīvas, man vispār grūti teikt, ka tur var kaut ko pielāgot. Ja mēs sākam sākotnēji par to runāt. Protams, tur ir jābūt speciālista pārraudzībai, tam es piekrītu. Lai noteikti kuri no tiem vingrinājumiem, vai tiešām visi ir jāpilda un cik reizes, kādu atkārtojumu skaitu, cik reizes dienā, jo šiem pacientiem ļoti skaidri jānosaka. Tiešām, kā Dal2 minēja. Ja jums ir spiediens, jūs nepildat. Ja jums sāp galva, jūs nepildat. Ja jums kāda cita veida problēmas, kāpēc viņš to vingrinājumus nedarīt. Bet tīri no tādas audiologopēda perspektīvas, es pat neredzu ko tur pielāgot, jo viņs ir ļoti tikai vienam ļoti konkrētam traucējumam. Tas ir pacientam, tiešām pie labās puslodes, pie vertebrobasilāra insulta, kur es varētu teikt, ka, jā jums tagad jātrenē mēle un lūpas. Bet praktiski, nekam citam vingrinājumi tur nav. Ne arī tādā aktivitāšu līmenī, lai trenētu runāšanu vai ēšanas aktivitāti kā tādu, tur nekā tāda nav. Un arī, tiešām, kaut kas, kas ir ļoti ekstensīvi, ir varbūt valodas rehabiltācijas virzienā. To noteikti būtu ļoti grūti attīstīt, bet faktiski šobrīd tādu funkciju nemaz nav. No audiologopēda perspektīvas, es nedomāju, ka tur varbūt tik daudz sākt ar vingrinājumu pielāgošanu dažādības ziņā, cik vienkārši izrunāt ar pacientu, cik bieži, ar kādu atkārtojumu skaitu un pie kādiem nosacījumiem viņš viņus nepilda.

50 [0:23:57.0] Dal1: Nu jā, bieži jau ir tā, ka šie insulta pacienti ir ļoti nekritiski pret savu saslimšanu. Mums jau bieži sanāk strādāt tieši ar pacientu tuviniekiem un tuviniekus mums ir jāizglīto tieši par šo te terapiju, pielāgoto terapiju konkrēti.

51 [0:24:16.0] Dal2: Es tā runājot iedomājos, ka tā pielāgošana varētu būt tāda kaut kāda, teiksim, standarta programma, kuru funkcionālais speciālists izvēlas konkrētus vingrinājumus. Bet, manuprāt, ka būtu, ja vispār uz to aizietu, šī te ieraksta funkcija, ka pacients pildot vai ko uz vietas nofilmē to, ko viņam liek un tad viņš arī skatās. Un tas ir reālā video un tādā veidā viņš varētu piedalīties.

52 [0:24:46.0] Dal5: Filmēšana ir laba atgriezeniskā saite arī speciālistam. Un arī speciālists var pārraudzīt kā viņš ir sanācis un labot nakamajā reizē [Dal2 un Dal5 atbalsta un piekrītoši māj ar galvu] (nesaprotams).

53 [0:24:55.0] Dal1: Man ir jautājums. Kurš to darīs? Kurš to filmēs? Kurš to sūtīs?

54 [0:24:58.0] Dal2: Tas jau ir cits jautājums.. (Dal5 komentē, nesaprotams. Dal1 pārtrauc).

55 [0:25:01.0] Dal1: Ņemo vērā visu to mājas vidi, to kontingentu kādi ir šie cilvēki, kādas ir šīs mājas vides. Es neticu nevienā mirklī, ka kāds to var izdarīt

56 [0:25:14.0] Dal5: Nē, es domāju, ka tas varētu būt ietverts jau programmā. Principā, viņš uzliek bildi, tā planšete tajā brīdī arī to filmē. Tas ir tā, jo, protams, ja mēs runājam par sekošanu līdzi un tiešam filmēšanu no kāda radinieku puses, tad nē. (nesaprotams) tiešām no mājas vides un kāda atbalsta sistēma viņam.

57 [0:25:31.0] Dal2: Jā, es visu laiku domāju kā pielāgot šos vingrinājumus, ka cilvēks atnāk pie

fizioterapeita, atnes to planšeti un viņi kopā vai nu izvēlās no tā kas ir piefilmē vēl klāt, kādu vingrinājumu, ko pacients pats veic, Tādā veidā. Piemēram, atnāk audiologopēds, viņš apsēdina viņu, piemēram, pie spoguļa. Liek viņam, lai vinš nofilmē un pacients pats skatās uz šiem vingrinājumiem un mēģina izpildīt. Es tādā veidā to domāju. Atgriezeniskā saite jau arī ir te, bet vēl ir iespēja, ka speciālists pats var papildināt atbilstoši vides apstākļiem, ko konkrēti viņš vēl varētu veikt.

58 [0:26:08.0] Dal5: Ja, kaut Individualizējot to, kas pašreiz tur ir iekļauts. Būtu ļoti grūti trāpīt kaut kādai pacientu populācijai - tā varētu būt par mazu.

59 [0:26:17.0] Mod : Tad es sadzirdēju, ka tieši speciālisti – fizioterapeits vai ergoterapeits, viņiem takā jānāk mājas vidē vai citā veidā jāizvēlās tos vingrinājums, un jāsatāda to programmu pacientam

60 [0:26:33.0] Dal1: Jā, individuāli tam pacientam (citi māj ar galvu)

61 [0:26:35.0] Mod: Vai ir kaut kāds cits veids, kā, piemeram, nepiesaisot speciālistu konsultācu, varētu veidot, pielāgot to programmu ?

62 [0:26:44.0] Dal1: Nē, es uzskatu šis nav iespējams

63 [0:26:47.0] Dal5: Piekritīšu, ka nē.. jo pirmkārt primāri pacients tiešām ir nekritisks, ar sapratnes traucējumiem, ar kognitīviem traucējumiem (nesaprotams). Vispār, respektīvi, viņš vienkārši nepareizi pilda to laiku kaut kādus vingrinājumus, par kuriem vispār jautājums vai tie ir viņam piemēroti.

64 [0:27:06.0] Dal1: Nu un arī tad tā pati mājas vides pielāgošana, lai vispār pacients spētu pildīt šos te vingrinājumus. Vai arī, bieži arī, ko ergoterapeits dara, viņš maksimāli vispār tam pacientam pielāgo to mājas vidi.

65 [0:27:22.0] Dal2: Es atceros tos pirmsākumus, kad veidojās šī te vigo programma. Ar ļoti foršu mērķi, tiešām, jo speciālisti pietrūkst. Un, ka šī planšete tiešām varētu būt kā atvieglojums, lai pacientiem tiktu šī, vismaz tā tika prezentēts. Kādreiz, kad pietrūkst speciālisti un tāpēc mēģināsim aizstāsim daļēji ar planšeti. Bet es arī piekritīšu saviem kolēģiem. Šiem te speciālistiem ir jāatnāk šajā mājas vidē vai nu atkal ir šis atlases kritērijs, kurā posmā šī planšete pacientam tiek iedota. Ja viņa ir iedota, piemēram, pēc kāda rehabilitācijas centra apmeklējuma, kad uz vietas šis speciālists pielāgo viņam šo programm un palaiž viņu mājas. Viņi viņu ir redzējuši, ko, kas, kā jādara pielāgo, palaiž mājās un tad tālāk cits jautājums, pēc kāda laika perioda viņš atkal pielāgo savādāk. Bet to, ka šis cilvēks, neredzot speciālistu, iedod viņam planšeti. Nu, tad mēs varam jebkurš būt gan dietalogs, gan zobārsts un tā, viss ir ļoti teorētiski.

66 [0:28:27.0] Mod: Piemēram, ja tā programma ir izveidota vai to izveidoja speciālists, vai tā tika vienkārši izvelēta no pieejamām programmām vai citā veida. Kas varētu būt veids, kā var pārliecināties par terapijas atbilstību šim pacientam, jau kad ir izveidota šī programma?

67 [0:28:47.0] Dal4: Tur jābūt *follow-up* no speciālistiem, tā ir cita viena lieta, kas mums tiešām nav. Būtu vajadzīgs arī ieguldīt pētniecisko laiku, kad, mēs varam piesaistīt tur Vigo, slimnīcu vai jebko, bet nav follow-up. Ir tādas jautājumu kopas, kuras citās valstīs izmantotas kā vairākas follow-up programmas pacientiem. Ja mēs tā to kopu pārceļam virs šīs planšetes, tad follow-up programma nozīmētu, ka ir iesaistīta gan slimnīcas vide, kur tas pacients arī ir izgājis cauri. Tad ir kāds speciālists, kurš vada pētniecisko darbu, piemēram ārsts uztaisa pētījuma protokolu un sazinās ar komunikāciju centru slimnīcā šiem pacientiem. Piemēram, Jūs, kā Vigo iesaistītie, tiem, kas ir iesaistīti, tiem, kuri ir ieinteresēti zināt, kas ar tiem pacientiem notiek, ir tiešām izveidots protokols ar sistēmu. Kas strādā neatkarīgi no tā kurš speciālists ir ielikts šajā darba vietā. Tas ir speciālists X, kuram programma ar follow-upu, ar pāris jautājumiem. Tad, kad šie jautājumi tiek uzdoti, viņi, es tagad neatceros to nosaukumu, tas bija par ikdienas aktivitātēm, par, nu takā, tur specifiska forma jautājumiem. Es ar tiem jautājumiem ilgtermiņā varu noskaidrot kāds ir pacienta stāvoklis funkcionālais. Vai viņš saglabājas tāds kāds viņš ir uz plato fāzes. Vai viņš vispār neievēro nosacījumus un viņš paliek arvien sliktāks, vēl kaut kas tur ir noticis. Tad, piemēram, ja tāda follow-up programma mūsu 50 gadu aprūpē ir izveidota, tad caur šo programmu noteikti var veikt atlasi

pacientiem, kuriem ir jāsniedz tālākas konsultācijas. Teiksim: O, šim pacientam ir slikti, takā rekur iesaistām komandas sapulci. Viss ir saistaistīts caur sistēmu, mēs varam, neatkarīgi no tā, kurš šodien ir darbā, kurš grib vai negrib to darīt, mēs varam veikt pacientu atlases, kuriem vajag tālākas konsultācijas. Caur kurām, izvērtēt to, kā viņam iet ar šo programmu. Jo šis ir vēl stāsts par to, ka ir jāiegulda vēl papildus cilvēki, kas gūst apmaksu un tā tālak, kurš to varētu darīt. Tā vienkārši zīmēt griestos, kuriem mēs tagad zvaīsim pa jaunu, mēs nevaram. Jo, tā kā šī ir joma, ko var darīt arī ar pētniecību. Es jau noskaidroju, ka, piemēram, tā vienkārši zvanīt pacientiem vai kādam prasīt caur sistēmu kā jums iet, es nedrīkstu. Un arī jūs tā nedrīkstat darīt, mēs tāpat tā nevaram darīt. Jābūt sistēmai, tā ievērojot pacientu konfidencialitātes nosacījumus, drīkst vispār jautāt par viņu veselības stāvokli. Un, ja šīs aplikācija vienkārši kā rotaļlieta viņiem ir mājās, tas nedod pievienoto medicīnisko feedbacku pēc tam atpakaļ mums uz akūto posmu. Un, ja viņi nav pieskatīti ilgtermiņā, tas nav īpaši noderīgi. Ir vēlviens veids kā uz šo visu skatīties. Tie cilvēki, kas šobrīd ir seniori, kaut kad būs viņsaulē. Varbūt šī programma vispār netiek veidota šī brīža senioriem, bet tiem, kas jau uzaug ar šo te viedierīču sistēmu un varbūt tiešām tas briesmīgi izklausās, bet varbūt tas ir paredzēts cilvēkiem, kas būs pieradušāki pie sistēmām un varbūt mēs to nemaz nevaram sagaidīt no šī brīža, 70 gadnieks visu laiku nobloķējot sistēmu, viņu izmanto. Varbūt šis ir stāsts par 10 gadiem uz priekšu. Nu lūk, un brīdī, kad ir izveidots follow-ups, kas iesaista, gan slimnīcas vidi, gan vēl mūsu slimnīcas komunikācijas sadaļu, gan teiksim, firmas, kas kaut ko vērtīgu dara kopā. Ja katrs no mums strādās atsevišķi pa savam, tad tas rezultāts īsti nebūs. Tā aplikācija ir laba, bet nu, viņā ir trūkumi. Un bez ārsta pieskatīšanas atkal sanāk, tur tā, es arī varu ieguglēt internetā ko darīt ar insulta vingrojumiem. Vienkārši te tas ir smuki apkopots vienā vietā, bet.. nu..

68 [0:32:51.0] Dal5: Nu sanāk tā, ka pirms papildināšanas vajadzīgs jēgpilns (nesaprotams).

69 [0:32:54.0] Dal4: Jā, ir vajadzīgs feedbacks ar tiem cilvēkiem, kas viņus akūtajā posmā atlasa un strādā. Varbūt tas prasās pēc vēlviena, kaut kādas.. tiešām pētniecības iesaistes ikdienā. Ar kaut kādu sistēmu.

70 [0:33:07.0] Dal5: Bet pēc sistēmas, pārdomātas.

71 [0:33:10.0] Dal4: Jā, bet tas bišķiņ iet jau ārpus tikai Vigo.

72 [0:33:15.0] Dal2: Un es vēl varbūt gribētu papildināt, ka, strādājot teiksim rehabilitācijā, nekas nav tik vērtīgs kā radinieki vai tuvinieki. Un šis darbs ar Vigo ir arī jāsāk ar tuviniekiem. Šaubos, kas šis insulta pacients būt tas, kurš visu sapratīs, pildīs, darīs. Šis te, atbalsta persona, kas viņu ikdienā atbalsta, viņi būs tie īstie. Tā mērķauditorija patiesībā, pēc lietošanas apmācībā, ir šie tuvinieki, kas to izpilda. Un vēl pienes laicīgi, un vēl nokoriģē, motivē kā šo te planšeti lietot. Palīdz lietot, palīdz un tā tālāk. Līdz ar to šis darbs, takā, ar to pacinetu atlasi, ir jāskatās no tuvinieku puses. Pacients var būt ļoti jauks, ar potenciālu, bet, kad viņš iegulstas mājās, gultā un mājās nav kas viņu ikdienā pieskata, pazūt jēga.

73 [0:34:12.0] Dal1: Pilnīgi piekrītu saviem kolēģiem.

74 [0:34:17.0] Mod: Jā, paldies! Šis jautājums jau vairākkārt izskanēja, par mērķa auditoriju. Tieši, pacientiem. Kā jūs uztverat, kāda funkcionāla līmeņa pacientiem būtu piemērota šī terapija un kāds būtu laika rāmis pēc pārciesta insulta, kad to varētu izmantot?

75 [0:34:42.0] Dal5: Viņiem jābūt "modificēti neatkarīgiem".

76 [0:34:43.0] Dal1: Tiem, kuri jau ir saņēmuši, jau pieņemsim, kaut kādu stacionāro rehabilitāciju un viņš jau ir atbraucis no vaivariem, viņš jau ir bijis fizioterapeita, ergoterapeita, logopēda rokās, viņš jau zin ko no viņa sagaida speciālisti un tad, lūk, šāda veida pacientam, es tikai varētu pagaidām.

77 [0:35:00.0] Dal5: Jo tas ir jautājums, jo piemēram, kā viņš skatās (nesaprotams) precīzāks. Jo arī, kā tu minēji, Vigo ir diezgan limitēti tie vingrinājumi (priekš audiologopēda) un arī fizioterapeits, ergoterapeits, audiologopēds izstāstītu daudz precīzāk stacionārā rehabilitācijā. Un kas vēl, viņš varbūt jau paspēj tos vingrinājumus iemācīties, ja viņš tur atrodas apmēram divas nedēļas. Takā, man šķiet, ka potenciāli varbūt pēc tāda subakūtā posma, bet es teiktu, ka tādam modificēti neatkarīgam, jo ja viņam ir ļoti lieli ierobežojumi atkarībā uz kustībām, viņš vienkārši (nesaprotams)

spējīgs izdarīt. Un, ja viņam nevar (nesaprotams) kaut ko ārā, viņš vienkārši nebūs spējīgs saprast. Un tad jau atkal mēs atkal atgriežamies pie atbalsta.

78 [0:35:42.0] Dal4: Varbūt šis mērķēts tuviniekiem pēc akūtā posmā. Tas ir vajadzīgs nevis pacientam, bet gan attiecīgi personai kas nodarbosies ar viņu aktīvi ikdienā. Vai nu tas ir fantāziju pasaulē, vai nu oficālā aprūpes centrā, kas šo teiksim izdara, bet tuvāk realitātei kāds ieinteresēts radinieks, kurš rūpējās par savu mammu, tēti. Varbūt tas ir viņam jārāda, jo pat, ja tas ir mamma vai tētis var lasīt, redzēt, izlasīt, tad, ņemot vērā, cik daudz demences, kognitīvi traucējumi, vaskulāri traucējumi, endoskopātijas, sensorie traucējumi, tas ir vēl kaut kas cits, kādēļ viņi ir nekritiski ikdienā. Varbūt tad tas drīzāk jārāda radiniekiem vai ieteikt radiniekiem ko darīt ar to mammu, tēti, brāli, māsu.

79 [0:36:30.0] Dal5: Jā, jo kas ir (nesaprotams), ka insulta pacienti bieži ir ar blakus slimībām, autoimūnām slimībām. Jā, tas nav tā, ka tu vari izolēt.

80 [0:36:37.0] Dal4: Sākot ar cukura diabētu, kur ir polineiropātijas, kad viņi īsti neizjūt to spiediena spēku, ko viņi tur kapā ar to planšeti. Takā, vēl ir tie "pulksteņstikla nagi", bieži, ka nevar pat uztrāpīt planšetes pogas. Tā aina noteikti ir pacientam, kas noteikti var uztrāpīt un saprot ko viņi dara. Tas tētis, mamma..

81 [0:37:01.0] Dal5: Viņi tā jau slikti saprot kā šo internetu lietot pārsvarā (nesaprotams).

82 [0:37:04.0] Dal4: Jā, jo, protams, mēs tā iedosim 35 gadīgam speciālistam, kuram ir (nesaprotams), smeķēšana vai idiopātisks nezinams iemesls. Protams, ka viņš iemācīsies Vigo planšeti, bet, es pilnīgi neredzu kā šo var izmantot seniors ar domāšanas traucējumiem pat bez insulta. Šobrīd tas ir domāts pacientiem bez domāšana traucējumiem un paredzēts ļoti, ļoti mazai insulta pacientu grupai. Un, ja dienā mums iestājas 10 pacienti. No tiem 10, varbūt 1 varētu attiecināt. Varbūt vienam var piešķirt un tad vēl ar radinieku iesaisti. Ja jūs to aplikāciju gribat palaist daudziem, tiešām mums visiem uzlabot dzīvi. Tad viņa ir jāpielāgo vieglāk, saprotamāk, ar runājošu asistentu, ar radinieku apmācību. (Dal5 komentē, nav saprotams). Lieliem, resniem logiem, tik lielām pogām.

83 [0:38:05.0] Dal2: Es varētu piebilsts, ja mēs runājam par pacienta funkcionālo stāvokli, šiem atlases kritērijiem. Atlases kritērijs varētu būt arī teiksim tāds, ka viņš var būt ar vidēju asistēšanu, varbūt, jā, varētu būt ar vidēju, bet, tad atkal šī te planšete ir tuvieniekam kā tāds uzskates līdzeklis. Un tad varbūt šī izglītojošā daļa noteikti ir jāpapildina ar pozicionēšanu. Ar tādām pamata lietām, ar izprati par tām. Tad, manuprāt, es pat teiktu, ka tad varbūt pat būtu lielāks pieprasījums šai planšetei. Jo sākuma posms ir ļoti smags. Jā, ļoti smags. Pacienti nesaprot ko, kā. Ar vides pielāgošanu, ar telpas pielāgošanu. Šie jautājumi ir primāri, ko šis radinieks uztrauc.

84 [0:39:01.0] Dal5: Jā, es teiktu, ka pašaprūpes aspektu, to vajag. Piemēram, varbūt ēšanu, tas ir viens. Un ģērbšanās, nu kaut vai, kā viņam vieglāk ir vieglāk (nesaprotams). Pat ēšana, kur ir ļoti izteikta ergoterapeita un audiologopēda sadarbība. Un tur tiešām tā aplikācija var palīdzēt, jo tas ir jautājums mājas rehabilitācijā ar ko speciālisti reāli strādā. Ja nav speciālists laikus, tad tur tā aplikācija varētu palīdzēt. Tas arī ir tāds reāls slieksnis, tikko kā viņš būs mājās un ir jāsāk domāt kā viņu pozicionēt, kā viņam staigāt.

85 [0:39:34.0] Dal1: Jā bieži, es atvainojos, ka pārtraucu, arī tuviniekus piemeklē izmisums no tās neziņas ko tagad iesākt, kā būt un ko man darīt. Un ko man tagad ar to mammu vai tēti darīt.

86 [0:39:47.0] Dal4: Par to vispār runājot, es atļaušos tagad pabeigt. Rehabilitācijas komandā pēc idejas ir arī psihologs un sociālais darbinieks. Ja jau tā aplikācija ir paredzēta praktiskai dzīvei, tur ir jābūt informatīvajai sadaļai. Informācijai par sociālo dienestu, kas tas vispār ir, kā tur var sazināties. Jābūt informācijai par krīzes tālruņiem, par to, kas vispār ir psihologa atbalsts, t.sk. radiniekiem. Kaut vai ielikts talrunis talrunī, ja viss ir slikti, kam viņi var piezvanīt tajā dienā. Jo arī vismaz ambulatorajās vizītēs, ja atnāk sieva līdzi vīram. Tā ir sieva, kurai vairāk vajadzīgs atbalsts, vairāk kā nekritiskajam vīram. Un, ja jūs to aplikāciju tiešām praktiski dosiet pacientiem, tur ir jabūt informācijai kas ir sociālais dienests vai psiholoģiskais atbalsts, jo tas arī iet kopā ar visu mūsu ikdienas darbu. Ar to vien, ka viņš vingrinājumus veiks, viņa diena nebeigsies. Ja mēs esam pavisam moderni. Un par kaut kādām pazīmēm - ja jūsu radinieks sāk atteikties no ēdiena, nu takā, šīs te depresijas un tādas pazīmes. Visi aizcietējumi, visas tās komplikācijas, kas vispār ir. Jābūt

tādam. Tā ir gudra aplikācija, tur jābūt algoritmam, ja jums ir šitāda problēma, piemēram, sociālā izolācija ar depresīviem traucējumiem, rekur jūs pārmet uz lapu, kurā ir informācija par psiholoģiskiem talruņiem. Vai arī psihologu reģistru, šeit var atrast speciālistu. Caur ģimenes ārstu var dabūt 10 nodarbības, tam tur jābūt vienā akcentētā vietā.

87 [0:41:22.0] Dal5: Jā, tas ir tas stāsts, kad tikko, sākot viņu katru dienu lietot, rodas tas jautājums kā šo (nesaprotams, Dal6 komentē, nav saprotams).

88 [0:41:29.0] Dal5: Tur ir jautājums. Jā,bet pēc tam (nesaprotams)

89 [0:41:35.0] Dal4: Piezvanīt, ja kaut kas notiek.

90 [0:41:38.0] Dal5: Jo tas labāk ne kā uzmundrinošie teksti. Ir jābūt kaut kādam resursam.

91 [0:41:43.0] Dal4: Vai arī, piemēram, jūs taisījāt aplikācijā visādus vingrojumus, palika slikta, tad viņš izmet tālruni par ģimenes ārstu dienas tālrunis pēc plkst. 17, lai viņš uzreiz nezvana ātrajai palīdzībai. No jums tas prasa tikai programmētāju darbu, bet mediķiem tas atvieglo visu darba dienu, jo tur ir tāds algoritms.

92 [0:42:00.0] Dal5: (nesaprotams)

93 [0:42:06.0] Dal2: Tam arī vajag medicīnisku uzraudzību.

94 [0:42:09.0] Dal5: Bet tas ir tas kā es to redzu pēc sarunām, varbūt arī nemaz nepietiek. Domāju, ka tam tur ir jābūt iekšā. Es tā domāju.

95 [0:42:16.0] Dal6: Tad jau tas ir ļoti, ļoti kritiskiem pacientiem. (Dal5 komentē, nav saprotams)

96 [0:42:20.0] Dal6: (nav saprotams) Stacionārā "Biķernieki" bija kritiskie pacienti, kas uzreiz no viņa atteicās, izmēģinot. Bija tas, ka viņš pārāk lika viņiem darīt. Viņi nevarēja izvēlēties savā secībā, kā viņi gribi. Ja viņš ir pietiekoši kritisks, lai pateiktu: "Šodien, man reibst galva, es negribu stāvēt kājās. Es gribu veikt vingrinājumus sēdus". Viņš to nevarēja darīt.

97 [0:42:40.0] Dal2: Viņš nevarēja pārlikt.

98 [0:42:42.0] Dal6: Jā, pārlikt. Tad viņš viņu aiztaisīja ciet un teica "labi, tā bija visa sadarbība, viss, atā." Cilvēks reāli viņu nav izmantojis, jo viņš nevar dabūt to, ko vajag un viss.

99 [0:42:51.0] Dal2: Viņš saka: "Tu neesi pabeidzis vingrinājumu" (citi dalīnieki komentē, nav saprotams)

100 [0:42:55.0] Dal6: Un viņš nepabeidz līdz galam tos kaut kādus vingrinājumus.

101 [0:42:58.0] Dal4: Bet tas jau ir programmēšānas jautājums. Mēs atgriežamies pie tā, ka tur būtu jābūt radiniekam. Varbūt viņu var pārgrommēt, tās sistēmas. Teiksim, video neuzkarās, ja tu negribi šito izdarīt līdz galam. Vai arī var programmēt tā, ka (nav saprotams). Tas jau ir IT jautājums, tas mums nav jārisina.

102 [0:43:19.0] Dal2: Atgriešoties pie šiem funkcionālajiem līmeņiem. Atbilstoši funkcionālajiem līmeņiem, atkarībā no līmeņiem ir jābūt galveniem vingrinājumiem. Par izglītošanas daļu - varbūt pacients arī guļošs, var iedot planšeti radiniekam kur būs paradīts elementārie vingrinājumi kā viņu var piecelt sēdus pozīcijā. Elementāri, kā var nopozicionēt uz ēšanas aktivitāti.

103 [0:43:42.0] Dal4: Kas ir izgulējumi.

104 [0:43:43.0] Dal2: Viss kā. Tāpēc tas līmenis ir atkarīgs kādu informāciju tajā planšetē viņiem dod. Kuram pacientam. Reāli tas..

105 [0:43:50.0] Mod: Paldies!

106 [0:43:51.0] Dal2: Es pabeigšu ātri. Šo planšeti es redzu kā elementu, bet kritiskiem, darbspējīgā vecumā, ja, tiem, kas varētu varbūt varētu iedot pārējiem, līdz ar to izkrīs.

107 [0:44:04.0] Dal4: Ar šo nevarētu nopelnīt, vismaz (nesaprotmas). Mums ir, cik tur tie tūkstoši pacienti gadā, tad tāda kā viņa (lietotne) ir uztaisīta, viņa nenestu ne pelņu, ne viņa veic medicīnisko biofeedbacku. Viņa ir jāuzlabo. Vai nu tad uz peļas celšanu (nesaprotams) pacientiem vai arī uz mediķu darba atvieglošanu ar apmācībām, jo nu, viņa ne pārāk laba priekš nekritiskajiem.

108 [0:44:30.0] Mod: mhm, Paldies. Es sadzirdēju, ka tieši tā informācija, kas mērķēta pašiem pacientiem, lielākoties jābūt ļoti vienkāršotai, ļoti uztveramai, varbūt tur palielināta teksta izmērs. Un, ko jūs teicāt saistībā ar pacienta kognitīvo stāvoki. Vai būtu nepieciešams veidot arī vairākus tekstus un vairākus interface veidus, atkarībā no pacienta kognitīvā stāvokļa? Vai jābūt kaut kādai testēšanai vai atlasei pašai lietotnei, izvēloties tur kāds ir kognitīvais stāvoklis un saprašanas līmenis tam pacientam?

109 [0:45:04.0] Dal4: Tur jau tā lieta, ka, ja viņam ir sapratnes traucējumi, arī radinieki bieži nesaprot, ka tur tādi ir. Un Latvijā, tā ir pārāk liela. Piemēram, demences nediagnosticēšana, kad arī radinieki, kas nav mediķi un ir super kritisks pret radiniekiem. Viņi nekad neuzspiedīs, ka manam tētim ir domāšanas traucējumi. Viņi pateiks, ka viņš vienkārši ir niķīgs, viņš tāds vienmēr ir bijis, jo tev jau aizmirstās kāds viņš bija pirms 10 gadiem.

110 [0:45:30.0] Dal5: Nu labi, mēs salīdzinām tikai (nav saprotams) visi nav (nav saprotams).

111 [0:45:33.0] Dal4: Atlak maza grupa.

112 [0:45:35.0] Dal5: Jā, tur es tev piekrītu. Teorētiski, ja viņi atzīst tāpat, padomāt kā var to visu interface pielāgot tādam pacientam, kuram ir izolēti uzmanības traucējumi, ne tikai atmiņas traucējumi. Kā to pielāgot. (Dal2 uzsāk komentēt, Dal4 pārtrauc)

113 [0:45:50.0] Dal4: Tā kā rotaļlietu. Izlec kaut kāds avatars kurš runājās. Kurš vizuāli kaut ko tur dod. Viņš kaut ko kustās, lai tev būtu kaut kur, kur fiksēt savu skatienu.

114 [0:46:03.0] Dal5: Nu jā, tas jau atkal uz kaut ko balstās, ka viņam jābūt attiecīgam izvērtējumam, kur tā problēma ir.

115 [0:46:08.0] Dal2: Atkal vajag tad speciālistu, kas to izvērtē. Vienalga, šim te funkcionālajam vai kādam no citiem, bet tāpat vajadzīga novērtēšana.

116 [0:46:12.0] Dal5: Jā, jo klasiski radiniekiem nav zināšanu kas ir vizuālās teplas traucējumi.

117 [0:46:19.0] Dal2: Tas ir viens un otrs - vajag pielāgot to planšeti visvienkāršakajiem, visjūtigākiem, ar tekstu pa vienam vārdam. Bet tā jau planšete. Ja. Tas nav kaut kāda grāmata, ko var pāršķirt, ko visi saprot. Tur ir jāieslēdz podziņa, tur jāiespiež arī podziņa. Kognitīvi sataisiet visvienkāršāko variantu, bet tā ir tehnoloģoja, kuru viņi nesaprot. (citi dalīnieki komentē, nav saprotams).

118 [0:46:44.0] Dal4: Tad mēs atkal iesim pie radiniekiem.

119 [0:46:46.0] Dal2: Atkal ejam tad pie radiniekiem. Radinieki jau kognitīvo testu netaisīs (citi dalībnieki komentē, nav saprotams).

120 [0:46:54.0] Mod: Labi, paldies. Un, runājot par pašiem speciālistiem, kāda veida apmācības būtu piemērotas, lai izglītotu speciālistus par programmas lietošanu un saturu, kā jūs uzskatāt?

121 [0:47:05.0] Dal2: Sākumā, ja es kā speciālists, noteikti sākumā gribētu redzēt šo programmu kaut cik tādu jau gatavu, teiksim tā. Tad es parakstītos viņu apmācīties un veltīt savu laiku, lai es redzētu jēgu, ko es apmācos. Takā, jautājums no kā ir jāsāk, vai no funkcionāļu apmācības, kas visu to apmācās vai atkal no tās programmas īsti izveides.

122 [0:47:30.0] Dal5: Es piekritīšu laikam. Man šķiet, ka tas nav tas ar ko būtu jāsāk, jo šeit kā audiologopēds, diemžēl, tā es neredzu ko daudz kur tur būt mācīties tīri no tādas, no lietojamības viedokļa, jo tur vienkārši nav daudz tādu vingrinājumu kam (nesaprotams), kā teikt izpausties tajā terapijas procesā. Visbiežāk jau dotajā stāstā tāds kā tas ir šobrīd funkcionālo speciālisti būs tie, kas papildinās viņu. Viņa ir kā tāds palīgrīks terapijai. Un man šķiet, ka viņi aizmirst tādu ekstenzīvo rehabilitāciju satura ziņā. Varbūt jautājums, kā izglītot funcionālās speciālistus rīkoties ar viņu, dotajā brīdī man būtu grūti pateikt kuram pacientam kā speciālists, es viņu varu pielietot. Nu tas ir tādā ziņā, manāmi šaurāks loks, šaurs pacientu loks.

123 [0:48:24.0] Dal4: Droši vien mans jautājums ir: cik jums ir laiks, finanses un jauda kā iesistīt speciālistus programmas veidošanā? Jo, ja jūs viņu veidosiet roku rokā, ik pa laikam ar šādām fokusgrupām un sarunām. Un konstruktīvu kritiku vai "paldies šis ir forši". Tad tomēr tā programma var aiziet vienā vai otrā labā virzienā, ja viņa tiek veidota atrauti un, ja jums ir ierobežots laiks un visas finanses beigušās. Viņa ir jānokoplektē, nu tad nebūs aršana. Bet, ja jūs esat gatavi ik pa laikam veidot komunikāciju ar speciālistiem procesā, kāmēr tas tiek veidots. Tad, teiksim, rekur ieteikums, no audiologopēdiem šis šobīd ir par maz. Jūs pieliekat klāt, satiekamies pēc mēneša un kolēģi saka: "Jā, tagad ir foršāk, bet vēl varētu šito ielikt iekšā ". Un tad varbūt arī ieņemtu tādu prakses vajadzīgumu, bet tad tam ir jābūt konstruktīvi, ik pēc laika atskaitēm.

124 [0:49:20.0] Dal5: Es piekrītu.

125 [0:49:21.0] Dal1: Es arī pievienojos kolēģiem, viennozīmīgi.

126 [0:49:26.0] Dal4: Tad mēs varētu redzēt ļoti foršu rezultātu, manuprāt.

127 [0:49:29.0] Dal2: Tas būs laikietilpīgs un finansiāls ietilpīgs jautājums.

128 [0:49:33.0] Dal5: Jā, tieši tā.

129 Dal2: Bet tad varēs viņu kaut kur tālāk bīdīt, caur šo te valsts finansējumu un vēl kaut ko. Bet, ja man tagad kāds prasītu, tad man liekas, ka vēl par jēlu bišķin tā.

130 [0:49:47.0] Dal5: (nesaprotams), jo citā projektā, ko mēs taisījām, mums visu laiku bija fokusgrupas, fokusgrupas, kamēr mēs beigās dabujām kaut ko jēdzīgu. Tas bija baigais process.

131 [0:49:59.0] Mod: mhm, paldies! Mēģināsim noformulēt kādus ieguvumus un trūkmumus jūs saskatād digitālā asistenta izmantošanai mājas vidē insulta pacientiem?

132 [0:50:13.0] Dal2: Es kā ieguvumu noteikti redzu to, laikam jūsu sākuma mērķi. Kad viņš teiksim, mājas rehabilitācijā, viņš var atvieglot. Tādā ziņā, ka viņš samazināt nodarbību skaitu reāli šim te reālajam klātienes nodarbību skaitam funkcionālā speciālistam, bet neizņemot viņu vispār ārā no procesa. Funkcionālajam speciālistam atnākt 2x nedēļā nevis 5x nedēļā, kā viņam vajadzētu nākt, aizstājot šīs darba dienas ar šo te planšeti. Kur ir adekvāts saturs un pielāgots saturs. Tas varētu būt pluss, ja. Un mīnusus man liekas, ka mēš šeit ļoti daudz iskaitījām, kas un kā, bet primāri kas ir. Viņš ir, bet funkcionālaie speciālisti jums būs tāpat vajadzīgi un jums būs jāstrādā, manuprāt. Es pat netiktu, es pat nedomāju, ka šo planšeti mēs varam panākt to, ka stacionāra funkcionālie speciālisti izvērtēs kuram to planšeti iedot mājās. Jo, reāli, ir jāatnāk mājās un tad mēs tikai varam saprast kuram mēs viņu dosim. Līdz ar to šī sadarbība, manuprāt ir neizbēgama. Primāri šiem te speciālistiem, kas strādā reāli mājas rehabilitācijās, jo šī te planšete ir paredzēta šim te posmam. Tas ir tas. To, ka viņš var atvieglot kaut kā daļēji. Es teiktu, ka noteikti jā, bet tikai pēc šīs funkcionālā speciālista ierašanās mājās. Vairāku, ja tie ir tie, kurus nozīmē.

133 [0:51:36.0] Dal1: Nu jā, tad ir, man liekas, ka jāiet roku rokā ar funkcionālajiem speciālistiem. Ar šo te planšeti. Tiešām tas funkcionālais speciālists pielāgo, izglīto, lai tas ir pats pacients, vai tas ir tuvinieks. Jā, tad tas varētu strādāt. Un vēl, regulāri nākot, updatot , jā, šos te vingrojumus. To pielāgot, pārrunājot, ka tas bija labi, kas bija mazāk labi. Kas patika, kas nepatika. Un tikai tādā veidā, mijiedarbojoties šiem speciālistiem ar šo programmu, tas, manuprāt, varētu strādāt.

134 [0:52:14.0] Dal5: Es piekrītu. Man liekas, ka tas var būt labs atbalsts. Tā kā, vienuviet ir pieejama visa tā informācija, viss tas saturs, ko mēs daram. Jo, kas ir patlabam tāds liels mīnuss - mēs nākam ar savām lapiņām, tur vingrinājumi rokām, vingrinājumi mēlei, vingrinājumi ar bumbām. Uz atsevišķas lapas rehabilitācijas barošanai, vēl kaut kādas rekomendācijas pozicionēšanai, vēl kaut kāds izglītojošs buklets. Bet man liekas, ka potenciāls varētu būt tāds, ka tas varētu būt vienuviet. Un ja tas viss ir tajā planšetē, tas ir liels atvieglojums. Nav jautājums, ka viņam tā lapa aplieta ar kafiju un tur pusi nevar izlasīt, jo arī notiek mājas vidē. Un man šķiet, ka tas ir ļoti labs un palīdzētu organizēt visas tās rekomendācijas. Tad papildus arī tas viss ko kolēģes teica. Ja tas ir veids kā strādāt tajā brīdī, kad speciālists nav pieejams, tas nozīmē, ka tā terapija var būt intensīvāka, jo kaut kādas dienas viņš dara pats. Jo mēs arī zinam, ka tas iet roku rokā ar to, ka rehabilitācijas speciālistu īstenībā pietrūkst. Viņu nav pietiekami daudz. Bet arī tad tas var būt tikai pārraudzībā, tad tas daļēji to problēmu risina. Nu tas, kas pie mīnusies, nu jā, mēs jau gana daudz viņus uzskaitījām. Bet nu labi, es atkārtošu no manas perspektīvas, tas lielākais mīnuss ir tas, ka viņu vajadzētu papildināt ar saturu.

135 [0:53:23.0] Dal4: Es no ārsta viedokļa arī domāju, ka šis būtu ļoti foršs veids kā papildināt ikdienas darbu, bet ārstam parasti tas lielākais mīnuss parasti tas, ka nav pietiekami laiks noinformēt pacientu par komplikācijām. Un, tātad, tas ko es jau teicu, praktiskās iespējas viņiem saņemt psihoemocionālu, sociālu atbalstu, pluss ģimenes ārsta informācijas. Es to redzu tā, ja jūs parādat pievienotu informāciju par diennakts ģimenes ārsta kontakttālruni, šie te atbalsta punkti un atsevišķi sadaļu. Kas vispār ir insulta komplikācijas, ar vizuālajaiem materiāliem. Kur veidojas klasiski izgulējumi. Būtībā izklaisās pēc manas kaut kādas prezentācijas, ko es rādu studentiem. Komplikācijas tādas, tādas un tādas. Viņas visas var atrast pakonsultējoties ar grāmatu, speciālistu, dakteri. Ja tas būtu tur iekšā, es to redzētu kā tik labu atbalstu komunikācijai ar pacientu. Jo es itkā izstāstu vienu lietu. Bāc, es aizmirsu. Viņš aiziet mājās, viņš ataisa vaļā un viņam tāds: "Oooo". Man tas nav tā, ka. Mums liekas saprotami, ka ir izgulējumi, bet tas pacienta radinieks ierauga sarkano pleķi un viņš domā kas tas vispār ir. Ja viņš īstenībā pamana. Bet, piemēram, ja būtu tā sadaļa, plus vēl tās funkcionālās lietas, tad pacienta radinieks nevarētu pateikt, ka es par to neko neesmu dzirdējis, ārsts mani nav informējis, programma neder. Bet, ja tas tur ir iekšā un viņš nav atvēris, tas ir cits stāsts. Un par interface, speciālisti IT to visu var saprogrammēt smuki. Lielas pogas, krāsas, interaktīvi. Ne tik daudz tas čats, kas tur lec ārā, bet vairāk interaktīvs bildīte. Būtu baigi forši.

136 [0:55:10.0] Dal2: Klausoties atkal ko Dal4 saka, atkal no akūtā posma, tad es drīzāk redzu, ka to planšeti iedod kā savu informāciju un tad viņam mājās piesaistās šie funkcionāļi, tad viņi to papildina. Atver to, kaut kādu informācijas logu un tad viņi saliek. Tādā ziņā Dal4 ir pilnīga taisnība. Jo tā laika ir katastrofāli maz. Pacientu apjomi ir nenormāli. Tad varētu ārsts iedot šo savu pamatinformāciju. Ar ko īstenībā šim pacientam ir arī jāsāk. Ar šīm komplikācijām, ar izpratni kas, kur kā. Piemēram, kur var funkcionālo gultu dabūt. Kādā veidā var iznomāt palīglīdzekļus. Mēs ļoti labi zinām, ka tā rinda ir ļoti gara. Un tas ir jautājums kas bieži vien atnāk pacientiem un viņu tuviniekiem jau mājas esot. Jo šeit viņus, nav iespējams nokārtot šo jautājumu. Nu lūk. Un ārsts iedod šo te planšeti, tādā veidā to savu informāciju un tad jau, atnākot mājās, pārējie papildina.

137 [0:56:12.0] Dal4: Jā, sakomplektējas par jēdzīgu instrumetnu ikdienā, viss kopā (Dal3 komentē, nav saprotams).

138 [0:56:19.0] Dal2: Informācija kaut vai tā planšete. Jā, mēs visiem dodam tos papīrīšus un viņam jāzin kur pieteikt, to pašu, mājas rehabilitācijas pakalpojumu, viņs var būt planšetē.

139 [0:56:31.0] Dal5: Jā, man arī liekas, ka es to redzu nevis kā atsevišķu, prom no visa stāvošu planšeti, kas aizvieto funkcionālo speciālstu, bet drīzāk uz ko viņa būs vērsta kā palīglīdzeklis. Vai viņa būs kā palīglīdzeklis radiniekiem vai viņa būs kā papildus instruments, ko lieto rehabilitācijas speciālisti. Bet kā vienkārši, prom no visa stāvošu, planšeti, kas atrisinās visas tās esošās problēmas, nu nē.

140 [0:56:53.0] Dal2: Jā, gan, gan. Pacients bez tuviniekiem. Insulta pacients. Vai nu viņš ir tik modificēti neatkarīgs, ka viņs pats visu atradīs. Gan internetā, gan ambulatorajā rehabilitācijā, gan rehab. centros. Vai nu tad, viņš tomēr ir nedaudz ar to asistēšanas līmeni, vairāk vajadzīgs. Tad šim ir jābūt kopā, roku rokā. To nevar atjaukt.

141 [0:57:16.0] Mod: Es gribētu precizēt. Runājot par tiem ieguvumiem un trūkumiem, jūs minējāt trūkumus pašā lietotnē un procesā kā tas izveidots. Kādi ir trūkumi varētu rasties pašam pacientam vai terapijai šīs lietotnes izmantošanas rezultātā?

142 [0:57:33.0] Dal4: Tas, ko minēja kolēģi. Ka apnīk, čarkst, viņš neļauj pabeigt. Kaut vai es šo vingrinājumu zinu, man ir garlaicīgi skatīties, viņu nevar skipot un darīt kaut ko citu. Tur ir tomēr liela barjera no lietām ko var darīt. Piemēram, fizioterapijas vingrinājumi. Ja to videoklipu var skipot, pārtīt, nomainīt. Mazāk tas čats, vairāk tās opcijas mainīt vingrinājumus un tādas lietās. Bet blakus nosacījumu, ka šo darīt 3x dienā. Visu nedēļu. Svētdien paņemiet pauzi, padzeriet kafiju, bla bla. Tad visticamāk, ka viņiem būs tas. Nu kā, kāpēc "*Fitbit"* ir tik veiksmīgas programmas? Viņas ir interaktīvas, dod to dopamīnu no paveiktā darba. Skaņas, krāsas, vēl kaut kas, baigi forši pabeigt videoklipu (nesaprotams). Bet, ja man ir jāpagaida un pēc dabas jau esmu nepacietīga, man būtu jādara šitais. Man apniktu, jo man nav nekādas apbalvojuma sistēmas par paveikto. Es nevaru neko nomainīt, man paliktu garlaicīgi.

143 [0:58:41.0] Mod: Tad es pareizi saprotu, ka pacients vienkārši nepildīs vingrojumus un atteiksies no izmantošanas un viņam terapija nenoritēs?

144 [0:58:50.0] Dal4: Jā, viņi vienkārši neizdarīs. Viņi pametīs.

145 [0:58:56.0] Dal6: Tā kunze, kas man pildīja. Bija divas vienlaicīgi. Viena ļoti organizēta, aktīva, vecāka nedaudz par mums un otra bija 90 gadīga. Un viņām bija... Piemēram, tai 90 gadīgajai tas čats gāja daudz par ātru. Viņa, pirmkārt, nesaprot vai tur ir īsts cilvēks, kas ar viņu runā vai nē. Kāmēr viņa man jautā vai viņš ir īsts vai nē, viņa ir nokavējusi kas viņai ir jādara, kas ir jālasa. Attiecīgi, tai otrai kundzei viss bija par lēnu. Viņa nesaprot kāpēc viņa nevar pati izvēlēties, pati sakārtot pēc savas paššajūtas šodien. Bet iespējams, ka pēc pacientu kritiskā līmeņa, kāds funcionālais speciālists vai ārsts, salāgo visus šos faktorus. Cik ātri viņš iet, nu jā.

146 [0:59:44.0] Dal4: Nu jā, šis arī patiesībā, ātrums ir mainīgs (Dal6 komentē, nav saprotams)

147 [0:59:45.0] Dal1: Vienvārd sakot, pielāgošana.

148 [0:59:52.0] Dal5: Viens ir visas šīs lietas, ko jūs jau minējat. Ka viņš dažādu iemeslu dēļ var nepildīt. Bet otrs ir tas, ka viņš pildīs. Un, varbūt, viņš var būt tik pacietīgs, ka viņš sagaidīt, kad viņš beidzās un būs ļoti apzinīgs. Un jautājums ir vai viņš izpildīs pareizi. Jo nav jau kas tajā mirklī pārraudzīs vai viņš izpildīja. Tas ir tas risks pacientam.

149 [1:00:14.0] Dal4: Un mēs atgriežamies pie tā, ka tas ir palīglīdzeklis. Atnāk vienreiz speciālists, vienreiz viņš savā nodabā, mums atkal jāstrādā kopā. Un, ja tas atkal atgriežas pie naudas jautājumiem, tad jāraksta projekts saistībā ar veselības ministriju un bla bla. Lai šis neapstātos, jo mēs gribam pārāk daudz.

150 [1:00:32.0] Mod: Paldies! Un pēc iepazīšanās ar šo terapijas palīglīdzekli, tehnoloģiju, kā jūs vērtējat tās atbilstību aprakstītajam mērķim?

151 [1:00:45.0] Dal2: Nu te man liekas, ka mēs jau ļoti daudz par to atbilstību runājām. Ja to visu video interviju salāgo un satranskriptēt, jūs atradīsiet daudz atbildes uz visiem jautājumiem.

152 [1:01:00.0] Dal5: Kopsummā tas mērķis ir labs un ja viņu vēl varētu iznest ar aplikāciju, tad mēs piekrītam. Bet tur vēl ir kur tiekties attiecībā uz pilnveidojumiem.

153 [1:01:09.0] Dal2: Tur vēl daudz darbs,

154 [1:01:12.0] Mod: Tad mēs runājam par šķēršļiem, ko varētu pārvarēt, lai varētu nonākt pie tā iecerētā mērķa?

155 [1:01:20.0] Dal5: Jā.

156 Dal4: Jā. Mēs ierosinam biežāk būt kontaktā, kas iet roku rokā nevis atraudi no insulta vienības.

Drīzāk kopā.

157 [1:01:30.0] Dal2: Latviešiem ir raksturīgs katrs savā stūrītī kaut ko dara un nesadarbojas un beigās sanāk kaut kas labs vienam, kaut kas labs otram. Bet kopā nav lietojams. Takā, jā, tur vajag.

158 [1:01:43.0] Dal4: Būtībā. šī nav pēdējā reize, kad tas tiek apspriests (nesaprotams).

159 [1:01:48.0] Dal2: Bet jūs jau gana ilgi. Es nezinu, cik ilgi, jūs, Aleksandr, esat šajā programmā, bet viņa jau diezgan ilgi tā palēnām, palēnām, palēnām kustās, bet virzība ļoti lēni notiek.

160 [1:01:58.0] Mod: Es nekomentēšu savu iesaisti, jo tas ir ārpus šīs sarunas (dalībnieki smejas)

161 [1:02:05.0] Dal2: Tas nav jautājums. Tas ir retoriskais tāds.

162 [1:02:07.0] Mod: Ā,skaidrs.

163 [1:02:10.0] Dal2: Tas nav jautājums cik ilgi. Es vienkārši pa vidu dzirdu, jau ļoti ilgi šo te. Ka tāda doma ir, tāds "o, yeah, doma riktīgi laba" un viņu var attīstīt. Un caur mērķa auditoriju, vienkārši mērķa auditorija ir ļoti liela, tāda ziņā, ka insulta pacientu ir šausmīgi daudz. Diemžēl dēļ statistikas, bet viņa ir arī ļoti sarežģīta. Ja šie te atlases kritēriji ir ļoti jūtigi pret visām šīm. No visādiem aspektiem. No sociālajiem aspektiem, viskaut kas tur ir iekšā apkšā. Tāpēc ir grūti šo programmu salāgot. Būtu tur rokas lūzums, būtu daudz vieglāk. Bet tas ir, viņiem ir daudz funkcionālu izrietošu seku, ka tas būtu ļoti grūti uzreiz iedot konkrētu planšeti. Konrkētiem vingrinājumiem. Vienalga, šī te funkcionālā speciālista piesaiste ir nepieciešama.

164 [1:03:02.0] Mod: Paldies par jūsu vērtīgiem viedokļiem. Patiešām. Vai ir kaut kas, ko mēs palaidām garām un jūs gribētu piebilst par digitālo asitentu "Vigo" mājas rehabilitācijas ietvaros?

165 [1:03:18.0] Dal1: Es vēl no savas puses laikam varu novēlēt, lai nepadodās. Kad, ideja ir laba. Tiešām priekšā vēl ir ļoti liels darbs vēl vajadzīgs.

166 [1:03:31.0] Dal5: Es piekritīšu. Man šķiet, ka tiešām, tākā mēs teicām. Ja tas mērķis tiks sasniegs ar šo programmu, kas ir iecerēts. Tad tā ieguve ir ļoti stabila. (nesaprotams) Ko tiešām varētu izpildīt ar *Vigo*, bet tur vēl ir gana garš process. Bet man šķiet, ka ir tā, ka arī speciālisti ir gatavi sadarboties, ja tiešām vajadzīgi praktiski padomi. Jo, ja viņa ieviesīsies praktiskā lietošanā, tad es to redzu tā, ka mēs noteikti arī, neizbēgami, būsim vieni no tiem, kas viņu arī pielietos kā intervenci. Ja vēl vairāk tas būs kā apmaksāts pakalpojums. Var novēlēt tikai veiksmi visā šajā.

167 [1:04:08.0] Dal2: Es noteikti gribu piebilst par to sadarbības iespēju. Jo ir tā, ka, ja jūs izveidojat šo programmu. Jums liekas, ka viss ir ļoti labi, bet, ja funkcionālajam speciālistam nepatiks, viņš arī neieteiks. Viņš apzināti, neapzināti, zinot, ka tur ir kaut kas nekorekts iekšā, viņš bremzēs arī šīs te planšetes attīstību uz priekšu. Ja tas ir ļoti korekti, cik nu tas ir iespējams tajās, sadarbība un tā, tad arī tā planšete aizies cilvēkos. Viņa aizies. Tas viss aizies ļoti ātri. Bet, ja tas saturs ir tāds, nu tāds, nu tāds. Tu trīs reizes padomāsi vai ieteikt,jo pēc tam jau atsauksies uz tevi. Latvija ir ļoti maza. Man iedeva tādu vingrinājumu un beigās man bija tas un tas. Ir labs saturs, mēs iesim. Ir labs saturs, mēs arī labprāt apmācīsimies kā viņu lietot. Lai šo saturu veidotu, ir jāveido, tiem cilvēkiem, kas reāli šajā visā vidē ir. Bet baigi forši. Jau sen.

168 [1:05:19.0] Mod: Milzīgs jums paldies.
