# Supplementary material for: Use of the Digital Assistant Vigo in the Home Environment for Stroke Recovery: Focus Group Discussion With Specialists Working in Neurorehabilitation
Source: JMIR Rehabil Assist Technol. 2023 Apr 14;10:e44285. doi: 10.2196/44285 (PMC10148207; doi:10.2196/44285)
Supplement: Multimedia Appendix 2 [file rehab_v10i1e44285_app2.pdf]

1 [0:00:00.0]
2 [0:00:38.0] Mod: Sāksim ar nelielu iepazīšanos. Īsi, lūdzu, pasakiet kādu rehabilitācijas profesiju jūs pārstāvat un kāda ir jūsu darba pieredze saistība ar insulta pacientu rehabilitāciju? Negaidiet aicinājumu. Kas pirmais sāks.

3 [0:00:55.0] Dal1: Nu, tad es varu sākt. Mani sauc Ilze. Es strādāju kā ergoterapeits. Iepriekš sāku kā medicīnas asistents ergoterapijā. Nu jau gadu strādāju un jau gadu saskaros ar pacientiem pēc insulta.

4 [0:01:16.0] Mod: Paldies!

5 [0:01:17.0] Dal2: Tad es turpināšu. Kaut gan esmu zem segvārda, mani sauc Anna. Esmu ergoterapeite. Jau kādu laiciņu strādāju profesijā. Nu 2 gadus strādāju neiroloģijas nodaļā un no šī gada augusta mēneša esmu iesaistījusies tādā kā Vigo projekta grupiņā un mēs, tātad, piedāvājam šo Vigo izmantošanu insulta pacientiem. Skatāmies kā strādā tālāk.

6 [0:01:53.0] Mod: Paldies!

7 [0:02:00.0] Dal3: Es esmu Zane, strādāju kā audiologpēde. Sāku kā prakses vietā un pēc tam kā medicīnas asistents audiologopēdijā un tagad, nu jau no jūnija, kā audiologopēds strādāju. Līdz ar to tā saskare ar insulta pacientiem sanāk, ieskaitot prakses laiku, aptuveni gads.

8 [0:02:24.0] Mod: Paldies!

9 [0:02:27.0] Dal4: Mani sauc Ieva, es esmu fizioterapeite. Strādāju rehabilitācijas centrā nu jau trīs gadus. Ar insulta pacientiem darbojos, esmu saskārusies kādus 4 gadus. Prakses laikā, skolā, arī nācās saskarties.

10 [0:02:48.0] Mod: Paldies

11 [0:02:55.0] Dal5: Mani sauc Anna. Esmu ergoterapeite. Strādāju rehabilitācijas centrā nu jau 6 gadu. Ir saskare ar insulta pacientiem visu laiku. Pirms tam gan strādāju pediatrijā un sociālajā veselības aprūpē personām ar garīgās attīstības traucējumiem, bet šobrīd, jā.

12 [0:03:18.0] Mod: Paldies visiem! Es gribētu sākt ar jautājumu - kā jūs komentētu plašsaziņas līdzekļos pieejamo informāciju par digitālo asistentu Vigo?

13 [0:03:38.0] Dal1: Es varu sākt. Nu, ja godīgi, tad tādos kā plašsaziņas pārlūkos, es īpaši daudz neesmu redzējusi informācijas. Arī nekad neesmu redzējusi tādas kā reklāmas, piemēram, klipiņus vai kaut ko tādu. Par to īstenībā, tā nē.

14 [0:04:05.0] Dal2: Jā, es arī varētu pievienoties šim viedoklim. Jo pirms es ar Vigo biju saskārusies kā tajā grupiņā, tad es neesmu vispār neko par to neesmu redzējusi. Nu, tagad, pēdējā laikā, sociālos portālos, facebook ir izlēcis ārā šis Vigo. Bet pirms tam.

15 [0:04:31.0] Dal4: Es nezinu, varbūt es kaut ko jaucu, bet pāris gadus atpakaļ, kad sāka veidot šo divi puiši, man liekas izdomāja, ja? Vai es kaut ko jaucu? Tad es kaut ko dzirdēju par šo aplikāciju, bet tagad es neko neesmu dzirdējusi. Izņemot, piedaloties šajā pētījumā.

16 [0:04:52.0] Dal5: Jā, nu man ir cita pieredze, jo tiklīdz es uzzināju, vispār, kaut kādu pirmo, es nezinu, vārdus "Vigo" un tamlīdzīgi. Tad es viņiem jau piesekoju facebook. Nu tas jau bija, es nezinu, cik sen atpakaļ. Un līdz ar to, tā kā es viņiem sekoju, man visu laiku met viņu reklāmas. Tā kā, es diezgan daudz spiežu like un, manuprāt arī share pogas. Līdz ar to, manuprāt, pie vainas ir kaut kādi algoritmi, kas ir gan facebook, gan instagram. Jo, piemēram, tu vairāk sakomunicē ar konkrētu, es nezinu, vienalga vai tas ir pakalpojums vai mājas lapa, man viņš visu laiku. Kā es atjaunoju facebook, visu laiku ir tikai Vigo. Vigo tur, Vigo šeit, Vigo storijos. Nu, pilnīgi viss, viss man ir saistībā ar Vigo. Manuprāt, katru dienu, nu vairākas reizes. Bet es domāju, ka tas ir atkarīgs no šiem te algoritmiem. Nu, ja mēs neiesaistāmies, nespiežam šo te like, tad viņš gan jau arī neizlec, nu tiem, kas vispār neseko viņu lapai. Man tā šķiet, vismaz arī pēc tā ko pārējie kolēģi arī minēja.

17 [0:06:02.0] Dal3: Es tagad precīzi neatceros kā, bet man bija izdevies piesekot šai facebook lapai. Kā es par to uzzināju, es tiešām neatceros. Visticamāk, ka meklēju informāciju bakalaura darbam vai prakses laikā kaut ko, meklējot kaut ko par insulta par insultu vai tamlīdzīgi. Tad attiecīgi, man interneta pārlūki piefiksēja to, par ko interesējos un tad man facebook gan parādījās kaut kāda reklāma un tad es piesekoju. Nu, jā man nav tā, ka es tur varbūt katru dienu kaut ko daudz pamanu, bet, ik pa laikam, kādu aktuālu informāciju redzu. Bet tieši facebook, citur gan nē.

18 [0:06:41.0] Mod: Labi, varbūt ķersimies pie pašas lietotnes satura. Kāds ir jūsu viedoklis par izglītojošās informācijas un vingrinājumu atbilstību insulta pacientu rehabilitācijas nolūkam?

19 [0:07:07.0] Dal2: Nu, es mēģināšu atbildēt uz šo jautājumu. Principā ir tā, ja tu esi cilvēku redzējis un izvērtējis, tad var salikt šos kompleksiņus. Piedāvātos kompleksiņus, takā izveidot šo programmu, bet pēc visiem, viņu iespējām, nu takā, taisīt no baltas lapas, takā pa vienam vingrojuma likt un ir iespēja likt tādus kā pa vairākiem vingrojumiem kopā, kompleksā. Bet rezultātā, ja tu ieliec šo jau sagatavoto vingrojumu klāstu, tad bieži vien ir situācija, kad no 3 vingrojumiem, piemēram, 1 nevar izpildīt cilvēks. Tad īsti, nu, tas bišķiņ takā neder, jo tu īsti nevari izlabot šo te piedāvāto kompleksiņu. Tas man tā liekas bišķiņ, bet principā, izvērtējot cilvēku, sataisīt šo vingrojumu programmu ir diezgan ātri un vienkārši.

20 [0:08:10.0] Dal1: Jā, es laikam piekritīšu tam, ka ir daudz dažādu opciju, dažādiem vingrinājumiem un ar tām pašām ikdienas aktivitātēm dažādām. Kas varētu cilvēkam palīdzēt. Tā kā, variācijas ziņā tur ir ļoti daudz viskautkas, ko varētu pielietot.

21 [0:08:37.0] Dal3: Es varbūt vairāk pakomentēšu no tāda kā audiolopēda skatījuma. Tie svarīgākie vingrinājumi, kas ir būtiski tieši artikulācijā un mīmikā iesaistīto struktūru stiprināšanai un aktivitātes uzlabošanai, ir iekļauti. Un principā tādi, arī kādus lietojam ikdienā darbā ar pacientiem. Vienīgi man, piemēram, tajos video likās tas temps par ātru. Un atkal, nu, saprotu, ka to tempu īstu nevar pielāgot un tad, ņemot vērā to pieredzi, cik parasti ātri to pacienti spēj izpildīt. Man tas tempiņš likās tiem vingrinājumiem nedaudz pa ātru. Protams, ka no audiologopēda skatījuma, tā ir konkrēta pacientu grupa, kuriem ir vājāka tā artikulācija, mīmikā iesaistītās struktūras, bet, piemēram, es tā īsti neredzēju, ka būtu materiāli, piemēram, afāzijas pacientiem vai kaut kam tādam. Tur noteikti vēl ir izaugsmes iespējas. Teiksim, audiologopētam tas ir tāds diezgan šaurs pacientu loks, kam to pagaidām var pielietot, no audiolopēda skata punkta.

22 [0:09:46.0] Dal4: Es arī gribētu papildināt to. Jo, piemēram, no mana skatījuma, šie vingrinājumi der tādiem diezgan spējīgiem pacientiem. Nebija neviena vingrinājuma, kas ir guļus pozīcijā. Pacientam, kam ir smagāks stāvoklis tiem, kas nevar vertikalizēties, līdz pat sēdus pozīcijai. Pat nebija vingrinājumi uz bilateriālām kustībām, kas man liekas insulta pacientiem ir ļoti vajadzīgs un ļoti bieži nepieciešams. Bija tādi vingrinājumi, tādiem diezgan spējīgiem jau pacientiem. No mana skatpunkta, ko es ievēroju. Kas vēl. Paskaidrojumi vingrinājumos it kā bija labi, saprotami, skaidri. Vienīgais, vēl iedomājos: pacients, kuram nav pieejams tāds telefons vai planšete. Kā viņš varēs takā izpildīt šos vingrinājumus. Vai arī slikti redz. Viņam tas mazais ekrāniņš. Nu grūti viņam būs, man liekas. Tad viņam noteikti vēl vajag ar kādu radinieku tos vingrinājumus veikt vai terapeita uzraudzībā. Jo man arī sākumā, paskatoties to aplikāciju, radās jautājums - kāds ir aplikācijas mērķis? Vai terapeita darbu atvieglot vai uzlabot pacientam mājās to vingrinājumu izpildi. Tas bija arī viens jautājums, ko es esmu sev pierakstījusi. Kāds bija tas mērķis, ko es arī gribētu uzzināt no jums. Kas man netapa skaidrs īsti. Tāds ir mans viedoklis.

23 [0:11:16.0] Mod: Mans uzdevums šodien neatbildēt uz jautājumiem. Mans uzdevums ir salikt informāciju, bet diskusijas laikā mēs noteikti daudzus viedokļus varēsim sadzirdēt.

24 [0:11:30.0] Dal5: Jā, es piekritīšu Dal4 un arī Dal3, noteikti. Jo, es jau arī tātad, fokusgrupas vadītājam minēju, ka pacientiem es nevienam vēl neesmu paspējusi piedāvāt Vigo. Bet es esmu pati mēģinājusi šos te vingrinājumus. Gan viņus salikt, gan arī, piemēram, mēs darbā mēģinājām izpildīt šo te novērtējumu pēc Kanādas nodarbes veikšanas mērījuma. Arī to saprast. Un es teikšu tā, labi, man nav kognitīvo funkciju traucējumi, bet dažas lietas, lai cik mēs rūpīgi pētījām šo Vigo programmu, ir tomēr diezgan apgrūtinošas. Itsevišķi man, piemēram, nepatīk tādus lielus, paskaidrojošus tekstus. Bet tad es iedomājos, ka tas ir pacients pēc insulta un viņam nāk tur viens

tas čata lodziņš ar to paskaidrojošo tekstu un tad vēlviens tev nāk kaut kāds lodzīņs atkal. "Vai Jūs vēl sāpratāt?" Tad vēl tev pajautā vai tu saprati to, ko tu vai saprati jau pirms tam. Un tad tev arī, ir tādad šis, labi vingrinājumi ir daudz, dažādi. Ir arī ikdienas aktivitātes. Bet arī, piemēram, es arī nospiedu vingrinājumus, manuprāt, līdzsvaram. Nu ko gan arī Dal4 ir skatījusies un pētījusi, vingrinājumus šim te līdzsvaram. Un ta viņi sākās un viņi, piemēram, iet šīs te 10 minūtes, manuprāt, ja. Bet takā apstājas un, ja tu nevis pat varbūt nogursti, bet, ja tev paliek garlaicīgi vai tu gribi kaut ko pamainīt, tad īsti tur tādas opcijas nav. Es saprotu, ka aplikācija saprot, ka tu viņu nevari izpildīt un, ka ar tevi kaut kas nav kārtībā. Nu tā, vismaz cik es skatījos, jo arī, piemēram, šie te artikulācijas vingrinājumi. Mēs mēģinājām arī vingirnājumus arī pašas pildīt, ar lūpām, ar mēli un 10 minūtes tu viņu pildi. Un tev nav nekāda uzmundrinājuma pa starpu. Nu takā, va tas vispār ir okay vai nav okay. Nu labi, es jau saprotu, ka tur nav otrs cilvēks klāt. Bet nu, diezgan gari. Gari tie vingrinājumu kompleksiņi un tad tu arī nevari saprast vai tu visu pildi pareizi, kad viņš beigsies. Tādā veidā. Un tad es arī iedomājos, ka tas ir vairāk tiešām bez kognitīvo funkciju traucējumiem. Cilvēkiem pēc insulta, tādiem spējīgiem. Kā jau arī kolēģe minēja. Jo es iedomājos tos pacientus kuri man ir šonedēļ bijuši darbā. Nu, ja es viņu apmācītu šo te aplikāciju un programmu lietot, un pēc tam, ja viņam būtu mājās. Nu viņš viens pats nevarētu. Visi tie, kas man ir bijuši pēc insulta, neviens no viņiem viens pats nevarētu to veikt. Neviens nevarētu viens pats veikt. Es domāju, ja mājas ir cilvēks - meita, vīrs, sieva. Vienalga. Tad tas būtu viegli, bet tādam pacientam, kuram vēl ir arī kognitīvo funkciju traucējumi, varbūt arī redzes traucējumi pēc insulta. Nu tad diezgan sarežģīti. Bet kā jau es minēju, es neesmu vēl ļoti daudziem piedāvājusi.

25 Mod: Paldies! Sniegti diezgan plaši komentāri. Ne tikai par saturu, bet arī citām lietām, bet atgriežoties pie satura. Jūs komentējāt vingrojumus, bet vai ir kaut kas sakāms par izglītojošo informāciju, kas ir pieejama tajā lietotnē? Vai pievērsāt tam uzmanību.

26 [0:15:08.0] Dal2: Manuprāt, tā sākuma informācija ir šausmīgi gara. Tas aizņemt diezgan daudz laiku. Vismaz tad, kad es esmu piedāvājusi šo programmu. Tas aizņemt diezgan ilgu laiku kāmēr cilvēks iepazīstās ar šo Vigo programmu. Un pirmās manas emocijas, pirmo reizi piedāvājot šo programmu, bija tādas, ka viss ir slikti. Viss ir, nu tas, emocionālais kā tas tiek pasniegts tam cilvēkam, man personīgi ļoti nepaitka. Un arī tiekoties ar Vigo pārstāvjiem, es šo izteicu, ka man liekas, vai tiešām cilvēks, kurš stāsta par šo aplikāciju nevar kaut vai nedaudz tādu pozitīvo. Nu nezinu. Man tas viss likās ļoti nomācoši, bet tas ir tikai mans.

27 [0:16:01.0] Mod: Es precizēšu. Vai tas ir ilgums informācijas vai tas ir veids kā tiek pasniegta informācija?

28 [0:16:06.0] Dal2: Ilgums un veids. Veids, pirmkārt, kas man personīgi nepatika ļoti. Bet tas ir katram savs. Bet arī ilgums. Diezgan gari tā informācija tiek pasniegta. Tas, es nezinu, man liekas, ka tas ir neveikli.

29 [0:16:26.0] Dal1: Ja mēs runājam par to izlītojošo informāciju, tad kad viņš. Es tur skatījos tās dažādas, par mājas vidi un tamlīdzīgi, tas tiešām bija ļoti gari. Nāca ziņa pēc ziņas un es jau sen aizmirsu ko viņš man tur iepriekš stāstīja. Tur prasītos pēc kaut kāda, tāda, konkrētāka saraksta, kas tieši ar kaut kādiem kontrolpunktiem. Vai arī, piemēram, ar jautājumiem, kas man tur nāks pretī un uz ko es atbildēšu, viņš man dos konrkētu risinājumu. Bet tiešām tur bija informācija daudz. Tādos blokos, kas nāca viena pēc otras un es jau sen aizmirsu kas tur sākumā bija.

30 [0:17:10.0] Mod: Paldies! Runājot par saturu, mēs pieskārāmies, man liekas, ka jūs vienbalsīgi izteicāt viedokli, par to, ka tam saturam jābūt pielāgotam pacientam. Kāds jūsu būtu veiksmīgākais veids kā pielāgot programmu atbilstoši pacienta vajadzībām?

31 [0:17:35.0] Dal1: Iespējams, ka tieši kādi sarežģītības līmeņi, ka tie ir dažādi. Ar, piemēram, kaut kādiem aprakstiem, kas tieši ir šajā sarežģītības līmenī. Kādam pacientam tas būtu domāts. Tad varbūt arī, kad speciālists varētu papildus pakoriģēt ko varbūt nē, ko varbūt jā.

32 [0:18:01.0] Dal4: Sākumā varēja norādīt to pacienta, kura puse viņam tur ir bojāta. Es domāju, ka varētu arī sadalīt pēc smaguma pakāpēm. Tur plēģija, vai viņam ir tikai rokas bojājums, vai kājas un rokas vai vispār funkcijas nav traucētas. Varētu sadalīt tādās smaguma pakāpēs cik smags ir pacients un attiecīgi tam tad būtu attiecīgi līmeņi. Attiecīgi vingrinājumi. Tādai smaguma pakāpei,

tādas, piemēram, intensitātes un grūtības pakāpes vingrinājumi. Tad attiecīgi varētu pielāgot pacientam. Lai gan, tur būtu jāpapildina tie vingrinājumi, jo tur ir tiešām tikai viegliem pacientiem uz doto momentu.

33 [0:18:57.0] Dal2: Man gribas teikt, ka īsti varbūt tur ir šī opcija, jo tad, kad pirms dod programmu pacientam tur ir tāds saraksts ar to vai pacients ir guļošs, pacients spējs veikt mobilitāti gultā, pacients spēj sēdēt neilgu laiku, pacients spēj sēdēt 15 minūtes bez pieturēšanas. Izejot cauri šim izvērtējumam, iedodas šie vingrojumi, ko tur var piemērot. Un atkarībā no tā ko mēs esam atzīmējuši, ko pacients spēj, tad ir šie bilateriālie vingrojumi vai nav šie bilateriālie vingrojumi. Vai viņam ir līdzsvara vingrojumi stāvus, vai viņam ir līdzsvara vingrojumi tikai sēžot. Tas ir tas, ko es esmu ievērojusi, dodot pacientiem šīs programmas. Izvērtējot to sākumu, tad iedodās tas vingrojumu klāsts. Ir tur arī vingrojumi cilvēkiem, kur ir diezgan smagā sākotnējā stāvoklī.

34 [0:20:09.0] Dal3: Es vairāk tieši domāju, ka būtu iespēja vai nu speciālistam pielāgot to video tempu, kā no audiologopēda. Tie vingrinājumi ir tiešām labi un viņi principā der visiem, bet atšķirās tas atkārtojuma reižu skaits, cik būtu vajadzīgs katram pacientam. Un arī tas temps. Būtu labi, ja speciālists var pielāgot. Piemēram, tas video kaut kā bija pēc tam sadalīts vai vairāki tie video opcijas - 5 atkārtojumi, 10 vai 15 katram vingrinājumam. Vai arī, tad nezinu, vai speciālists var kaut kādu tempu noregulēt tam video vai pats pacients var sev pielāgot tempu. Jo šobrīd, kādā tie vingrinājumi tika rādīti, bija diezgan ātri. Pat cilvēkam bez funkcionāliem traucējumiem. Teiksim, ir ko just, izpildot tos vingrinājumus. Var izpildīt, bet tas ir bez traucējuma. Zinot kādi parasti ir pacienti, tas varētu būt diezgan sarežģīti. Zinot pacientus, viņi jau grib turēt līdzi tam tempam, ko viņiem rāda priekšā nevis divreiz lēnāk pildīt. Takā vairāk par to atkārtojumu skaitu un par tempu, tie būtu tādi ieteikumi, kā varētu pielāgot.

35 [0:21:23.0] Dal1: Es gribētu vēl papildināt par iepriekš teikto. Par to, kur ir par vingrinājumiem, ka ir iespējams izvēlēties kāds tas pacients ir. Būtu labi, ja būtu iespējams arī izvēlēties kādā veidā viņam informācija tiks pasniegta. Kad tas teksts, piemēram, nebūs garš, ka tur būs vienkārši "Aiziet", "Stop", "Nē", "Jā". Bez gariem tekstiem. Ir pacienti, kas vienkārši nespēs to izlasīt, negribēs to izlasīt. Viņi ieraudzīs, ka tur būs daudz teksta un pateiks: "Ejiet jūs no kuries nācāt ar to lietu". Viņš vienkārši sabīsies no tā teksta daudzuma.

36 [0:22:10.0] Mod: Labi, piemēram, Jūs kā speciālists pielāgojat to programmu pacientam vai kāds cits, kurš nav speciālists iedava programmu vai programma pati saģenerēja to progrmmu pacientam. Kā jūs pārliecinātos par to, ka terapijas programma ir atbilstoša konkrētajam pacientam?

37 [0:22:36.0] Dal5: Varbūt, ka viņš pēc tam izpilda kādu, nezinu, novērtēšanas veidlapu. Vai, tā pati apmierinātība. Viņš veic tos vingrinājumus, viņš veic šo te programmu. Tad iespējams, ka, ja var redzēt automātiski, ka viņš ir izpildījis visus vingrinājumus atbilstoši. Pēc šiem vingrinājumiem parādās - cik jums ir svarīgi šie vingrinājumi, cik jums bija noderīgi šie vingrinājumi, cik jūs jūtaties apmierināts ar šiem vingrinājumiem. Kaut kas tāds jau arī ir, bet tādā īsākā formātā. Lai nav tā, ka tiešām programma saprot, ka, ja tu 10 minūtes nepildi šos te vingrinājumus. Lūpu vingrinājumus, 10 minūtes, pacients vienkārši nogurst vai viņam ir garlicīgi. Lai programma nesaprot, ka tas vingrinājums nav viņam piemērots. Jo vingrinājums varbūt ir piemērots, bet viņam kaut kas varbūt neuzrunā. Vai nu šis temps, ātrums un tā tālāk. Tādā veidā varbūt, jā. Tad var redzēt. Ja viņam apmierinātība ir, viņš ir veicis to savu programmiņu. Nezinu, tos 5 vingrinājumus vai tos 10 vingrinājumus Ja var redzēt terapeits, kas to ir izpildījis tajā dienā, kad viņam tas ir noteikts, tur pirmdiena, trešdiena, piektiena un viss ir kārtībā. Tādā veidā, jā. Uzlabojumus, protams, jau var novērtēt pirms Vigo ar kaut kādiem ergoterapeita novērtēšanas instrumentiem un, piemēram, pēc. Un tā arīefektivitāti, tādā veidā.

38 [0:24:11.0] Dal1: Tas laikam atkarīgs arī no tā, kādā kontekstā tas notiks. Ja, piemēram, uzsāk pacients jau klātienē,esot kādā stacionārā, tad kopā ar to ergoterapeitu viņš var sastādīt to programmu un apskatīt vai viņš tiešām to var izdarīt. Ja tas notiek attālināti, tad noteikti ar komunikāciju. Ja nevar ar pašu pacientu sakomunicēt, tad ar tuviniekiem - kā viņam izdevās, vai visu izpildija, vai visu saprata. Tā, tieši komunikācijas ziņā.

39 [0:24:54.0] Mod: Varbūt tāds papildinājums par to atbilstības novērtēšanu. Kā tas pakalpojums ir

dotajā brīdī, ko jūs ieteiktu varētu mainīt paša pakalpojuma sniegšanas veidā, lai varētu novērtēt atbilstību? Vai dotajā brīdī to vispār var izdarīt pie šīs lietotnes stāvokļa, vai tā kā tā lietotne tiek nodota cilvēkiem? Vai par šo ir kādas domas vai komentāri? Varbūt jums trūkst informācijas?

40 [0:25:39.0] Dal5: Vasaras konferences laikā jau Vigo izveidotāji minēja tās iespējas, kā var šo lietotni un pašu programmu saņemt. Gan šajā veselības centrā, viņi katrā ziņā minēja, ka var nopirkt šo lietotni, programmu un speciālists aizbrauc pie viņiem. Uzstāda, novērtē un tā tālāk. Tad es pieņemu, ka arī mūsu rehabilitācijas centrā, ja mēs redzam, ka ir šis pacients, kuram ir nepieciešama šī te programma, tad mēs varam parādīt. Mēs varam apmācīt viņu. Ieteikt. Jā, tādā veidā arī, sadarbojoties ar Vigo, viņam nodrošināt kaut kādā veidā šo te, arī planšeti un pašu programmu. Tad jau mājaš viņš varētu strādāt atbilstoši tam, ko mēs viņam esam parādījuši un apmācījuši. Tas vismaz ir par mūsu darba vietu. Tā es iedomājos. Bet, jā, varbūt citās darba vietās ir savādāk. Varbūt citās darba vietās jau citiem kolēģiem ir pieredze kā pacienti tiek pie šī Vigo.

41 [0:26:49.0] Dal1: Es domāju, ka noteikti būtu nepieciešams kontrolvizītes pa vidu. Ka ergoterapeits atbrauc vai jebkurš cits speciālists atbrauc un tieši apskata kā tas notiek. Vai viss notiek tā kā vajag, vai ir efektīvi.

42 [0:27:10.0] Dal2: Uz doto mirkli tā mēs arī daram. Ja nodaļā mēs redzam cilvēku kuram to Vigo programmu varētu iedot pamēģināt, tad pirmajā dienā tu aizej novērtē, parādi, apmāci. Tad jau tu pēc, var redēt vai viņš lieto vai viņš nelieto. Nākošajā dienā atkal ieej istabiņā ar viņu aprunājies - viņš saprata, nesaprata, viņam gāja viegli, viņam gāja grūti. Šajā situācijā, ja ir tā iespēja tikties ar klientu, pacientu un izrunāt šo te visu. Tad šo visu var pakontrolēt un izdarīt. Bet, ja šī lietotne ir mājās.. Es tad īsti neredzu to efektivitāti, jo ik pa laikam, lai gan tas nav iespējams bez speciālista, bet, speciālistam ir ik pa laikam jāierodas un tur kaut kas jāveic un jāmaina. Tad to domu es īsti nesaprotu. Bez speciālista, manā uztverē, šī lieta nestrādā. Tāpēc ir šis video zvans iespējams. Es esmu vairākas reizes saņēmusi, ka pacients vēlās video zvanu, bet, protams, ka tas nav iespējams. Es nesēšu visu laiku pie programmas un negaidu, kad man zvanīs kāds pacients. Tādas iespējas īsti nav, tāds resurss īsti nav. Tā ir mana doma - ka doma ir laba, bet kā īsti tas mājas var strādāt.. nezinu, jā.

43 [0:28:47.0] Dal5: Jā, iespējams, ka tad būtu, varbūt kādam vienam Vigo šim te cilvēkam, kurš tad arī tikai strādā. Viņš sēž, piemēram, katru darba dienu no 8:00 līdz 18:00 pie sava Vigo mājās, pie telefona un tad attiecīgi visā Latvijā, kur arī cilvēki izmanto šo te. Tad viņiem arī tajās darba dienās ir teikts, ka viņi var zvanīt. Ja kas, tad tajā laikā. Un tad tas cilvēks atbild tikai par to. Viņš skatās vai tur viens izmanto, vai otrs izmanto. Viņam varbūt datorekrānā parādās pa Latviju 40 programmas, piemēram. Viņš redz tur, piemēram, Juris pirmdien, trešdien izmantojis. Ceturtdien nav izmantojis. Bet tur tagad zvana viņam, piemērma, zvana viņam no rīta, zvana viņam pēcpusdienā un viņš var pakonsultēt. Līdzīgi kā ir šie te, piemēram, kā 1188 kāreiz bija. Tātad šādi uzziņu dienasti un tad tas tā var būt. Es tā saredzu. Mēs arī tad, kad mēs darbā mēģinājām, mēs mēģinājām uzspiest šo te "Zvans draugam" video vai kādu tur palīdzības pogu. Takā, jā. Tā tas varbūt var būt. Ja tas Vigo kārtīgi aizies Latvijā, tad iespējams tādā veidā var kontrolēt un arī palīdzēt cilvēkiem. Nebūs jau tā, ka es iedošu, piemēram, 10 pacientiem. Viņiem mājās tiešām būs un es sēdēšu pie sava telefona visu dienu beigās. Viens nesapratīs, otrs nesapratīs ko darīt. Jā, takā, tādā veidā. Tā kā man arī pētijuma tēma ir bijusi par šīm te tiešsaistes tehnoloģijām tieši rehabilitācijā, tad es redzu tīri praktiski. Tad kad man bija dalībnieces, kurām bija jāuzmana viedierīču lietošana, tieši saistībā ar fiziskām aktivitātēm, tad bija tā, ka viņas tiešām zvanīja ik pēc kaut kādām dienām. Kas tur varbūt nav pareizi, kas ir pareizi un tā tālāk.

44 [0:30:48.0] Mod: Labi, tagad es dzirdu no jūsu puses, ka ir kāds speciālists kas to programmu iedod pacientam vai nu sākumā, palaižot viņu mājās, vai stacionārā esot. Vai kāds speciālists, piemēram, kā tagad bija piedāvāts, attālināti pārrauga to pacienta darbību vai atbild uz tiem video zvaniem. Pieņemsim, ka kāds viņu algo. Vigo vai NVD vai kāds cits. Vai ar attālinātu konsultāciju pietiktu šajā gadījumā?

45 [0:31:24.0] Dal4: Es domāju, ka nē. Noteikti vajag arī vēl kādu klātienes konsultāciju, jo attālināti tas jau būs tas pats, kas skatīties tajā planšetē. Varbūt mēs varam pateikt tās kļūdas ko mēs redzam un palabot tos pacientus, bet tāpat.. Domāju, ka klātienē. Klātieni nekas īsti nevar aizstāt.

46 [0:31:47.0] Dal1: Es domāju, ka te noteikti no tās smaguma pakāpes būtu atkarīgs. Vieglākam pacientam, tādam bez kognitīviem traucējumiem, tiešām ar viegliem traucējumiem, varbūt, ka pat pietiktu ar video zvanu. Varbūt.

47 [0:32:02.0] Dal2: Jā, es piekritīšu kolēģiem, kad tas būs izejot no situācijas cik smaga tā situācija būs. Kaut kur pietiks ar to video zvanu, kaut kur tas noteikti nedos to vēlamo rezultātu.

48 [0:32:17.0] Dal5: Jā, jo ja tā vaina būs, piemēram, tikai tajā, ka pacients nesaprot kā varbūt iziet no programmas vai kaut ko nospiest, vai pamainīt. Tad es domāju, ka to var arī telefoniski paskaidrot vai tiešām video zvanā kaut kā sazināties. Bet nu, ja tā runa ir par vingrinājumu izpildi, vai viņš tur pareizi vai nepareizi veic. Vai viņš nogurst un viņam parādās diskomforts un tamlīdzīgi. Tad tur būtu klātienes vizīte noteikti nepieciešama un tas jau kā līdzīgi kā arī mājas vizītes. Vienkārši par kaut kādu noteiktu, pieņemsim, samaksu. Tad speciālists sarunā un, piemēram, aizbrauc. Vai arī šis te Vigo, nezinu, darbinieki. Un paskatās, kas tad ir par vainu un paskaidro, parāda un tad jau pacients pēc tam varbūt tiek pats galā tālāk,

49 [0:33:12.0] Mod: Labi, mēs jau runājam par to kādiem pacientiem tā terapija var būt piemērota. Kāds ir jūsu viedoklis - kāda funkcionāla līmeņa pacientiem būtu piemērota šī terapija un kāds būtu tas laika rāmis pēc pārciesta insulta, kad to varētu izmantot?

50 [0:33:36.0] Dal2: Nu es personīgi neredzu patreiz to iespēju izmantot to planšeti tad, kad cilvēks ir pilnīgi guļošā stāvoklī. Tas laika rāmis, man liekas, ka ir ļoti grūti nosakāms, jo katra situācija ir ļoti dažāda, bet, ja cilvēks ir tādā stāvoklī, ka viņš tik tikko sāk mainīt pozīcijas gultā , tad es neredzu šo programmas izmantošanas iespēju. Tātad, tas būs jau cilvēks, kas jau ir sācis vismaz sēdēt jau. Pirmkārt, bez kognitīviem traucējumiem izteiktiem. Redze, dzirde, jo ir jābūt šai izpratnei lietot vispār šo planšeti. Un, sarunas valoda - latviešu valoda. Tikko tas nav, tad..

51 [0:34:40.0] Dal5: Jā, es piekrītu kolēģei noteikti. Jo ir jau arī pacienti pēc pacienta ļoti dažādi. Vienam, piemēram, šajā te kaut vai akūtajā fāzē nav izteikti funkcionāli, tik izteikti traucējumi. Citam, piemēram, akūtajā fāzē, kā jau kolēģes minēja ar smagiem traucējumiem, guļošs pacients. Bet, piemēram, paiet šie trīs, četri mēneši, pieci, nu varbūt pēc tam tajā subakūtajā fāzē, viņš jau spēj. Tas takā ir labs veids kā papildināt terapiju. It sevišķi, ja, piemēram, pacients dzīvo kaut kur perifērijā, nedzīvo Rīgā. Viņš varbūt dzīvo 200 km vispār no kaut kādas apdzīvotas vietas. Mums tā arī ir bijis, kad mēs ar pacientu runājam vispār vai viņš varētu aizbraukt pie fizioterapeita vai ergoterapeita, tur vispār nevar būt domas. Un pacientam pat tādam, kurš varētu izmantot šo te planšeti, tad būtu ļoti labi visu programmiņu. Bet, ja pacientam ir smagāki funkcionāli traucējumi vai kā jau kolēģe minēja, par šiem kognitīvās funkcijas ierobežojumiem, tad nē. Tas laika rāmis, es nezinu, var būt ļoti dažāds. Bet nu, es teiktu vairāk subakūtiem. Piemēram, tiešām arī hroniskajā fāzē, kad viņiem jau ir nepieciešams šīs kustības, aktivitātes. Labi, viņi jau visu zinās par insultu, bet tik un tā ir nepieciešams un vingrinājumi. Tīri padomājot, jā, vismaz mūsu darba vietā, tur 99% ir krievvalodīgi pacienti. Ja, es padomāju cik man bijuši latviešu valodā pacienti, piemēram, vakar vai aizvakar, vai šonedēļ kopā, tad tie varbūt bija viens. Vispār latvietis. Tādā veidā. Varbūt kolēģēm audiologopēdēm un fizioterapeitei ir komentāri.

52 [0:36:37.0] Dal3: Es principā varu piekrist kolēģiem. Manā gadījumā varbūt šeit tās balss instrukcijas nav tik būtiskas, jo tie vingrinājumi ir diezgan saprotami, ja tikai rāda. Bet par tiem kognitīvajiem traucējumiem gan. Bet programma, jā, visa tikai, ja latviski runājošiem, mums jau tā statistika ir līdzīga. Pacienti pa lielam tie paši apgrozās. Vairāk ir to krievvalodīgo.

53 [0:37:07.0] Dal4: Jā, es gribēju piebilsts tam, ka piekrītu, ka šo programmu būtu droši varēti izmantot, kad pacients jau ir izgājis kaut kādu rehabilitācijas kursu. Kad viņš jau pa lielam ir izgājis cauri tiem vingrinājumiem klātienē ar fizioterapeitu. Tā ir kā labā alternatīva mājās turpināt šīs te nodarbības, ja nav iespēja vai nu mājas rehabilitācijai vai doties uz dienas stacionāru vai ambulatori saņemt šo pakalpojumu. Bet tas ir nepieciešams insulta pacientiem, lai uzturētu savu funkcionālo stāvokli. Lai uzlabotu, ja tas ir iespējams. Tā kā, es domāju, ka tas ir pēc kaut kāda rehabilitācijas kursa, tad kā metodi var pēc tam izmantot šo te aplikāciju mājās, kad viņš jau zin šos vingrinājumus.

54 [0:37:58.0] Mod: Man liekas, ka Dal2 bija savādāks viedoklis par to, ka tie vingrojum ir saprotami, ja

viņus rāda vienkārši... Nē? Tas bija pārpratums ?

55 [0:38:07.0] Dal5: Nē, manuprāt, viņai bija viedoklis, ka tieši nav saprotami arī bez verbālām instrukcijām.

56 [0:38:13.0] Mod: Nē nē, par to es saku. Tad, vai ir nepieciešamas verbālas vai no valodas atkarīgas instrukcijas, lai saprastu vingrojumus pareizi?

57 [0:38:29.0] Dal2: Bet, ja iet runa par valodu, tad cilvēks, kurš nesaprot latviešu valodu, vienalga tur netiks galā, jo tur nepārtraukti ir jāatbild. Piemēram, izmetās lodziņš kur Vigo ar tevi sarunājās, tad zilajā lodziņā tev ir jāspēj atbildēt. Tad, piemēram, ir divi atbilžu varianti. Ja viņš latviski nesaprot, tad viņš netiek uz priekšu programmai. Tāpēc šī valodas izvēle, nu tas ir tas ko es pati redzu, jo es apskatot : "o, būs foršs pacients, varēs iedot Vigo programmu". Sākam runāt par valodu un viss, atkrīt šī iespēja, jo diemžēl nevar programmu lietot dēļ, ka neizprot valodu. Bet es gribēju vēl bišķiņ par to, ka tādā akūtā periodā, man liekas, ka kā tādu vīziju jau.. Es gan nemācēšu pateikt vai tāda ir, būtu labi, ja būtu iespēja apmācīt šos tuviniekus lietot tieši šo programmu. Jo īsti es nestādos priekšā kā cilvēks tādā akūtā fāzē, kur viņam tiek mācīta ģērbšanās, mazgāšanās, tā pati sviestmaizes uzsmērēšana, un viss. Ja viņam kāds nepalīdz, ja viņš spēj lietot planšeti, tad viņš to spēs izdarīt pats bez šīs programmas pamācības. Tad man liekas, ka šī programma būtu tieši derīga, lai izglītotu tos tuviniekus, kas izmantojot šo aplikāciju, var to sākotnējo darbošanos veikt jau mājās. Lai tad, kad viņš beidzot nonāk rehabilitācijas iestādē, šis pacients, ka viņš jau ir nevis gulējis trīs mēnešus mājās un gaidījis, bet jau darbojies. Tā man liekas, ka tieši tas tajā akūtajā fāzē, šis Vigo, būtu noderīgs tuviniekiem, kuri tiktu apmācīti, lai viņi var lietot viņu. Saistībā ar pacientu pozicionēšanu sākotnēji un tā tālāk un tā tālāk, tad ģērbšanās, mazgāšanās tā tālāk. Tas ir, jā..

58 [0:40:36.0] Dal1: Es noteikti piekrītu šim viedoklim. Tās tā kā palīdzības rīks tuviniekiem, lai viņi varētu skatīties ko tieši darīt, kā darīt. Nevis tam pacientam, bet paši skatīties līdzi un tad palīdzēt arī tam pacientam to darīt. Un tas tāds labs rīks terapijas pēctecības nodrošināšanai, jo ne vienmēr tie radinieki mājās īsti sapratīs, īpaši tagad, kad Covid atgriežas un atkal nevar īsti satikties ar tiem speciālistiem. Vai viņi sapratīs kā pareizi bija to kreklu vilkt un tas pacients arī īsti varbūt neatcerēties to konkrēto veidu un tad viņš var vienkārši apskatīties programma un zināt tieši kā vajag.

59 [0:41:26.0] Mod: Paldies par viedokļiem. Vai jūs varētu teikt, kādus ieguvumus un trūkumus Jūs saskatāt digitālā asistenta izmantošanai mājas vidē insulta pacientiem?

60 [0:41:43.0] Dal2: Ieguvums noteikti ir, tad, kad tev nav iespēja tikt pie sava fizioterapeita, ergoterapeita. Tāpat programmā, ja tu jau brauc no rehabilitācijas iestādes, tātad tev jau ir sastādīta programmiņa un tev ir savs šis digitālais asistents, tu vari darboties. Bet ir jābūt motivācijai darboties, jo tikko kā tev nav šīs iekšējās motivācijas, tā programma nestrādās.

61 [0:42:17.0] Dal5: Jā, es varu piekrist kolēģes teiktajam, ka, ja pacients dodas no rehabilitācijas centra, kad viņam programma ir gatava, viņš zin visu kā pildīt. Viņam varbūt pat, es teiktu, ka pat tā programma ir kā motivācija. Nevis, ka viņam ir iedota A4 lapa, kur sarakstīti vingrinājumi un tagad pildi, bet viņam rāda priekšā tos vingrinājumus. Viņam katru dienu tas plāns ir, ka viņam jāiziet tam, tam un tam cauri. No tāda audiologopēda skata punkta pagaidām ir ļoti ļoti maz. Tikai tas būtiskākais - mēle un lūpu vingrinājumi principā, bet pa lielam audiologopēds strādā arī ar balss traucējumiem, tur varētu arī paplašināt vingrinājumu klāstu disfonijas pacientiem. Arī no ieteikumu viedokļa, piemēram, disfāgijas pacientiem, kas būtu.. Par to, ko kolēģes iepriekš jau minēja, par tuvinieku izglītošanu, kad kaut kādi ieteikumi, ko darīt disfāgijas gadījumā. Kaut kādas kompensatorās pozas un arī vingrinājumi, ko tad varētu darīt un kādu vispār uzturu lietot un kā. Un, un, balss vingrinājumi un disfāgija. Tas ir tāds, kas liekas, kur varētu paplašināt, ko pacients pats varētu saprast, piemēram, un arī pildīt.

62 [0:43:35.0] Dal5: Jā, es pilnībā piekrītu arī ko kolēģes jau minēja, ka tas ir labs palīgs, ja šis te speciālists nav pieejams klātienē un jau pēc tam, kad cilvēks jau ir apguvis. Bet, ja no trūkumiem, tad jā, ka ir jābūt pacietībai arī, jo arī visu laiku ir tās atbildes. Viņš arī gaida, kā kolēģi minēja. Tev izlec lodziņš un tev visu laiku ir jāatbild. Ja tev tās pacietības nebūs, tad ir nu tā, tālāk jau tu netiksi.

Nu man tā bija, ka man tās pacietības ir nedaudz mazāk, ja jo. Jo tu tikai spied, tu spied, tev atkal viņš kaut ko jautā un tev jau apnīk. Un tad domā kas tālāk būs. Bet vēl ir jābūt, jā šīm kognitīvajām funkcijām. Ļoti labām, es teiktu. Jo es neatceros, kuru uzdevumu es atzīmēju, bet es mēģināju laikam par atmiņas traucējumiem vai valodas. Es atceros, ka tur bija attēls ar suni, kurš guļ. Attēls ar suni, kurš guļ un tur bija četri atbilžu varianti. Un es domāju, nu suns, kas man tur ir jāatbild. Nu, jums tur ir jāraksturo, kas tur tajā attēlā ir minēts. Es domāju, tur ir kaut kāds atbilžu variants "dendijs", "dingo", "bingo", "diendusa". Nu es domāju, ka man jāmin tā suņa vārds, ja suns, kā tad viņu sauc. Un beigās bija diendusa pareizais atbilžu variants, pareizais atbilžu variants bija diendusa. Bet es neiedomājos. Man, bez kognitīvajiem funkciju traucējumiem cilvēkam, man diendusa asociējas, ka bērns čuc, mazs bērniņš. Man neasociējas, ka pat, piemēram, pieaugušais čuč diendusu, jo pieaugušais parasti neguļ diendusu. Tur bija tātad man šī diendusa jāasociē savā prātā, varbūt tur audiologopēts varēs pareizāk to soļus pateikt, ko tad es darīju. Vai tā ir vispārināšana, vai kas, bet man savā prātā bija jāizdomā, ka man ir jāizvēlas tā aktivitāte, ko tas suns dara un, ka tā ir tieši diendusa. Man tas šķita grūts uzdevums. Tātad, bet tas bija viens no. Es tur vairākus ņēmu, jā, tas bija viens no. Un, tad protams, pie tā es padevos, pie tā uzdevuma. Un tad es nevarēju atrast kā iziet no šī, kā iziet no tās programmas. Jo, viņš man neļāva iziet. Vienkārši, viņš man spieda tālāk iet. Droši vien viņš gaidīja, kad es visu pabeigšu, bet es padevos dēļ savas neveiksmes. Nu lūk. Bet tas ir vienkārši mans stāsts, bet es domāju, ka tāda pieredze var būt arī pacientam. Nebūs jau tā, ka mēs varēsim, piemēram, viņam pilnīgi visam iziet ar viņu cauri. Takā varbūt tas laiks varbūt nebūs visam iziet noteikti. Tad es negarantēju, ka viņš mājās varbūt arī veiks. Līdz ar to, tām kognitīvajām funkcijām, manuprāt, ir jābūt nu salīdzinoši labām. Nu, lai nerastos kādas aizķeršanās kā man.

63 [0:46:37.0] Dal4: Man radās arī varbūt ierosinājums par tām uztura lekcijām un par miega izglītošanu. Varbūt tur var uztaisīt kaut kādas mazas īsfilmiņas, jo tiešām lasīt pacientiem, kam ir slikta redze vai nevar salasīt mazus burtus. It kā tur varēja palielināt, viss kārtībā, tāda funkcija ir. Bet lasīt visu laiku un spiest tās atbildes tiešām ir apgrūtinoši. Man liekas, ka vieglāk ir vienkārši tādu vizuālu īsfilmiņu uztaisīt par svarīgāko to informāciju un man liekas, ka to ir arī vieglāk uztvert. Citiem pacientiem, ar šiem uztveres traucējumiem, varbūt tas vizuālais paliks vairāk atmiņā nekā tā čata formāta lasīšana, kas ir diezgan apgrūtinoši un arī nogurdinoši kā arī kolēģi minēja.

64 [0:47:26.0] Dal1: Es pilnībā piekrītu kolēģu viedoklim.

65 [0:47:37.0] Mod: Ja mēs domājam par ieguvumiem, gribētos domāt par to, ar ko šī lietotne var uzlabot to terapijas un iznākumu pacientiem. Bet, ja mēs domājam par trūkumiem, kāds būs traucējums terapijai vai pacientam, ja viņš sāks to lietot un ir kaut kāds trūkums, kas kaut ko izdara? Es domāju vai nevar būt tā, ka Vigo lietošana var būt sliktāka par Vigo nelietošanu? Vai otrādāk, vai jebkura lietošana ir labāka nekā neko nedarīšana?

66 [0:48:21.0] Dal2: Viennozīmīgi jau, ja cilvēks lietos šo programmu, tas būs tikai un vienīgi pozitīvi. Es nezinu kā citās rehabilitācijas iestādēs, bet, piemēram, cilvēks pēc svaiga insulta, nonākot iestādē, parasti pēc mēnešiem diviem, trijiem atgriežas, atkarībā no situācijas. Ja viņš pa šo laiku neko nav darījis, kas diemžēl tā šad, pat diezgan bieži ir. Tad protams tas ieguldītais sākotnējais ir jau pazudis un tad atkal sāc visu no sākuma. Ja viņš būtu lietojis šo Vigo programmu, kura jau būtu sastādīta un viņš būtu motivēts darboties. Tad atgriežoties atpakaļ rehabilitācijas iestādē, viņš jau ir tātad solīti uz priekšu nevis kritis atpakaļ un mēs atkal varam strādāt tālāk. Mēs atkal varētu iedot Vigo programmu ar citiem vingrojumiem. Protams, tas būtu ieguvums. Bet jautājums ir par to lietošanas motivāciju. Ja cilvēks ir motivēts, tas strādās. Ja viņš izpratīs. Ja viņš nav motivēts, tad tas nestrādās.

67 [0:49:27.0] Dal1: Vienīgais, kas man likās, kad aplikācija uz doto brīdi ir sarežģīta un tas varētu to motivāciju arī nedaudz nosist. Varbūt tam pacientam tā motivācija ir ļoti daudz, bet viņš saprot, ka netiek galā. Viņam kaitina visi tie teksti. Tur var tādas dažādas garīgas problēmas rasties, pašpārliecinātība zust. Viņš var sākt sliktāk par sevi justies, ka viņš nevar izdarīt to, kas viņam varbūt būtu jāvar izdarīt.

68 [0:50:12.0] Mod: Labi, paldies. Tālāk gribētos jautāt, kāda veida apmācības būtu piemērotas, lai izglītotu speciālistus par programmas lietošanu un saturu?

69 [0:50:27.0] Dal5: Es domāju, ka noteikti praktiskas. Vismaz visas dienas, vai, piemēram, divu dienu

garumā. Kur tad arī tie, kuri ir izveidojuši programmiņu. Tīri, pirmkārt, visu tehnoloģiski pilnīgi pastāstu. Precīzi ko tad darīt, kādi ir risinājumi, ka kaut kas nestrādā un tamlīdzīgi. Jā, dienas garumā vai divu dienu garumā. Gan izglītojoši par teorētisko visu, par tehnoloģisko. Par vingrinājumiem. Tā, kā praktiski.

70 [0:51:13.0] Dal2: Es arī domāju, ka tikai un vienīgi praktiski to var apgūt, jo tikai paskatoties, cauri izejot šai Vigo programmiņai. Tas nav, tu neredzi īsi kā tas strādā. Vislabāk ir tad, kad kaut vai, ja ne kā savādāk nav iespējams, tad klīniskais gadījums. Tātad gadījums, cilvēks ir tāds un tāds un tāds. Tad tev pašam ir jāpaņem un praktiski jāsaliek tie vingrojumi un tad arī jāpaņem un visam tad dienas plānam jāiziet cauri. Tad tu vari redzēt, var izdarīt, nevar izdarīt, saprot, nesaprot. Bez tās praktiskās darbošanās, jā, tad to programmu tā diezgan grūti izprast un saprast.

71 [0:51:55.0] Dal1: Tā kā skolā - praktiski vienam uz otra (smejas).

72 [0:52:05.0] Dal5: Vai pat ideālā gadījumā ar kādu pacientu to organizēt. Piemēram, rehabilitācijas iestādē, ja pacients ļauj. Iespējams, tad piesakās arī pamēģināt tad tīri ar viņu. Vai vispirms izmēģināt dienas laikā viens ar otru, vienā dienā un pēc tam ir, piemēram, pēc divām nedēļām vai trīs ir kaut kāda atkārtota nodarbība un pa to laiku mums ir jāiedod vienam pacientam. Varbūt, kuru mēs uzfilmējam, es nezinu, kā viņš tur dara vai viņš saprot un tā tālāk. Tādā veidā. Tad mēs atkal tajā otrajā dienā tiekamies un analizējam. Tad jau katram ir bijis kāds pacients un var padalīties ar pieredzi.

73 [0:52:56.0] Mod: Paldies! Ja par šo vairs nav komentāri, tad es gribētu jautāt - Pēc iepazīšanās ar pieejamo informāciju un šīs lietotnes izmēģināšanu, kā jūs vērtējat, lietotnes atbilstību aprakstītajam mērķim? Vai arī kā jūs sapratāt to mērķi?

74 [0:53:26.0] Dal4: Es pašā sākumā minēju, es to mērķi laikam nesapratu sākumā.

75 [0:53:34.0] Mod: Šodien mēs runājam par Vigo Health tehnoloģijas izmantošanas iespējām mājas rehabilitācijas programmā. Par šo mēs šodien runājam.

76 [0:53:51.0] Dal2: Man liekas, ka tas mērķis tātad ir, lai cilvēks mājās esot varētu strādāt. Tas mērķis ir labs, lai cilvēks varētu strādāt bez speciālista. Vai tas vienmēr strādā? Manā uztverē tikai tehnoloģija bez speciālista.. īsti, man liekas, ka līdz galam tas nestrādā. Ir jābūt kaut kādai tik un tā, speciālista darbošanās. Jebkurā gadījumā tīri tikai šis Vigo. Nu nebūs, tur tik un tā būs vajadzīga speciālista iesaiste.

77 [0:54:34.0] Dal3: Es piekritīšu kolēģes teiktajam, ka, jā, ideja ir laba. Pacients turpina, bet ar noteiktu, ka viņš turpina pildīt to, ko viņš jau ir apguvis. Tā, ka viņš tagad iegādāsies programmu un viņu vienu reizi kaut kas novērtēs, viņam saliks programmu un teiks: "brauc mājās, pildi". Nezinu vai tas strādās. Bet, ja viņš ir, piemēram, pabijis rehabilitācijas centrā tās 10 dienas gājis visam tam cauri un apguvis, tad jā. Es domāju, ka tas ir ļoti labi, ka viņš var turpināt un viņam varbūt nav jāiet uz konsultācijām vai nodarbībām pie speciālistiem tik bieži. Jā, es piekritīšu, ka varbūt iekārta neaizstās cilvēku, bet tas ir kaut kāds variants kā viņš var uzturēt kaut kādu motivāciju. Viņš zin, ka viņam aplikācijā jāatver tā diena, viņam arī jāizpilda. Jā, labi, tie teksti tagad ir par garu, lai viņu tur motivētu varbūt, bet tā būtība, ka viņam tas var būt kā rituāls. Jāpaņem tā planšete un jāizpilda tie vingrinājumi. Man liekas, ka tas varētu viņu varbūt vairāk motivēt nekā paņemt to lapu un izpildīt tos vingrinājumus, bet tas atkal varētu būt katram individuāli.

78 [0:55:44.0] Dal4: Man liekas, ka tā ir laba ierīce kā terapeits pēc tam var paskatīties kā pacientam ir gājis no tās vienas nodarbības līdz nākamai nodarbībai. Pa to laiku viņš izmanto šo aplikāciju, darbojas pats un pēc tam terapeits paskatās cik daudz viņš ir vienā dienā izdarījis. Varbūt viņš to savu programmiņu pabeidz ātrāk kaut kādā veidā. Pauzīte, ko uzspieda, tad varēja arī ārā kaut kā tikt ārā no tiem uzdevumiem. Tas varētu būt kā tāds terapeitam, paskatīties kā pacientam pašam ir gājis. Viens pats pacients, tikko pēc insulta, bez rehabilitācijas, domāju, ka viņš to aplikāciju nespētu izmantot nekādā veidā.

79 [0:56:43.0] Mod: Paldies par viedokļiem. Mēs jau tuvojamies šīs diskusijas noslēgumam. Tagad beigās, vai ir kaut kas, ko mēs palaidām garām un jūs gribat piebilst par šī digitālā asistenta Vigo

pielietošanas mājas rehabilitācijas programmas ietvaros? Varbūt jūs gribat pie kaut kā atgriezties? Vai kaut kāda doma uzpeldēja?

80 [0:57:12.0] Dal1: Es varbūt atļaušos pieminēt. Ar kolēģiem darbā, veicot to novērtējumu, mēs ieraudzījām tādu sadaļu par seksualitāti tur. Bet tālāk, ejot cauri dažādiem izglītojamiem materiāliem un tam līdzīgi. Es neredzēju tur nekādu turpinājumu tam, jo man ļoti interesēja. Es gribēju paskatīties, ko viņš piedāvās par to. Bet nekādas informācijas nebija. Tad man ir interesenti, kāpēc tas tika pievienots tajā novērtējumā un mērķos, ja pēc tam tāda turpinājuma tam nebija? Varbūt es vienkārši neatradu to turpinājumu tam.

81 [0:57:57.0] Dal5: Jā, mēs mēģinājām arī salikt šīs nodarbes pēc svarīguma, manuprāt, tas bija Kanādas nodarbes veikšanas mērījums. Tad mēs arī salikām to pirmo kā seksualitāti un pēc tam mēs pamainījām visu. Tik un tā, tā programmiņa izlēca vienāda. Vai tā aktivitāte vai kas tas bija tālāk. Tad mēs īsti neredzējām. Bet varbūt mēs vienkārši neatradām. Iespējams tur ir kāds izglītojošs materiāls par seksualitāti pēc insulta un šādām aktivitātēm. Bet, jā, mums rādās tāds jautājums, kāpēc tad tā seksualitāte tur vispār ir minēta. Ja tur tālāk nav nekas izglītojošs vai pamācošs un tam līdzīgi. Mēs izmainījām krustu šķērsu šo aplikāciju tajā dienā.

82 [0:58:52.0] Mod: Vai ir vēl kādi komentāri ar ko jūs vēlaties padalīties?

83 [0:59:07.0] Dal2: Es tikai gribēju piebilst to, ka tas, ko es redzu lietojot šo lietotni, ka ir nepieciešams apmācīt tuviniekus. Tas būtu ļoti svarīgi sākotnējā posmā un pēc tam tad arī. Jo vairāk vai mazāk šis ir domāts tikai pacientiem, ne tuviniekiem, cik es tā izprotu. Bet man liekas, ka tās sākotnējais lietojums, tur būtu vajadzīgs apmācīt tos tuvinieku nevis pacientus.

84 [0:59:35.0] Dal5: Jā, es par to arī nebiju iedomājusies un tagad, kad kolēģe minēja, ka man arī šķiet tas ļoti ļoti labi. Arī to, ko audiologopēde minēja, tieši, tā kā mēs citreiz dodam rokasgrāmatu par aprūpi pēc insulta. Tas šis arī varētu būt kā tāds labs rīks. Nevis lapas, kā audiologopēde minēja, šīs te A4 lapas ar simts tur vingrinājumiem dažādiem, bet, ka tu vari tuviniekam parādīt šos te video materiālus un tuvinieks var video materiālā redzēt nevis skatīties uz lapas, kur vienkārši ir uzzīmēts kā pareizi veikt ģērbšanos, pozicionēšanu un tamlīdzīgi. Tas ir ļoti labs rīks tuviniekiem. Par to es iepriekš nebiju iedomājusies. Tad tuvinieks arī redz pareizo vingrinājumu izpildi un arī pacients pēc tam var veikt to atbilstoši. Pēc tam varbūt arī viens pats, ja tas tuvinieks nav klāt. Sākumā ar tuvinieku. Noteikti kā jau kolēģi minēja, nepieciešams sākumā apmācīt pacientam un pēc tam arī kaut kādu sekošanu līdzi, jo viņš varbūt aizbrauks ar to programmiņu un viss. Mums takā nav atgriezeniskās saites vai viņš to pilda. Varbūt ne mums kā terapeitiem, bet varbūt tiem, kas atbild par Vigo, viņiem ir jābūt kaut kādai tai atgriezeniskais saitei. Nezinu, ieplānotiem zvaniem pēc divām nedēļām vai pēc trijām. Arī pētījumos, ko es esmu redzējusi par viedierīču lietošanu, viņiem ir tie followup calls. Ir tie zvani, kad viņi, ja iedod jebkādu viedierīci, tad speciālists, kaut vai paredzēts vienu reizi mēnesī, viņš piezvana šim pacientam un pajautā kā jums ir gājis. Un tas viņu arī motivē. Nevis, ka tikai pacientam ir jāmeklē palīdzība, ja viņam nesanāk, bet arī, ka mēs piezvanām un pajautājam. Bet tā kā es minēju, ka nevar viens ergoterapeits izdalīt šo … (daļa no teikta netika ierakstīta). To tad ir jāda kādam, kurš tikai to dara. Tādas ir manas domas. Bet tas varētu būt tas, kas ir nākotnē un to, ko es lasu literatūrā, kas jau ir pasaulē.
