# Supplementary material for: Use of the Digital Assistant Vigo in the Home Environment for Stroke Recovery: Focus Group Discussion With Specialists Working in Neurorehabilitation
Source: JMIR Rehabil Assist Technol. 2023 Apr 14;10:e44285. doi: 10.2196/44285 (PMC10148207; doi:10.2196/44285)
Supplement: Multimedia Appendix 3 [file rehab_v10i1e44285_app3.pdf]

1 Mod: Ķerties pie pašas diskusijas. Sāksim sarunu ar nelielu iepazīšanos. Lūdzu, ļoti īsi pasakiet kādu rehabilitācijas profesiju jūs pārstāvat un kāda ir jūsu darba pieredze saistībā ar insulta pacientu rehabilitāciju? Kāds var sākt un vienkārši brīvā kārtībā turpināt.

2 Dal1: Labvakar visiem. Vai mani var dzirdēt? Mani sauc Anna, es esmu ergoterapeite un ar insulta pacientiem strādāju jau no 2009. gada. Tā ir mana pamatdarba vieta un vislielākā mana interese, jo uzreiz pēc studijām esmu uzsākusi darbu tieši neiroloģijas nodaļā. Paldies!

3 Mod: Paldies, Dal1. Kāds nākamais droši var ieslēgt mikrofonu.

4 Dal2: Labvakar! Mani sauc Ilze, esmu fizioterapeite. Ar insulta pacientiem strādāju jau divus gadus, bet tie nav pamata pacienti. Es strādāju gan ar insulta, gan ar cita veida pacientiem. Paldies!

5 Mod: Paldies Dal2.

6 Dal3: Labvakar visiem! Mani sauc Aija. Es ar insulta pacientiem arī tāpat kā Dal2 neesmu pamata kā pamata diagnoze, bet arī dažādām citām papildus diagnozēm, kā muguras sāpes, cita veida diagnozes. Bet strādāju kopš pagājušā gada. Pusotru gadu, varētu teikt, kopš es strādāju ar insulta pacientiem kopumā.

7 Mod: Un kādu profesijas pārstāve tu esi, Marta? Es ne nesadzirdēju.

8 Dal3: Fizioterapeite.

9 Mod: Fizioterapeite. Paldies.

10 Dal4: Labvakar visiem. Mani sauc Agnese. Es esmu ergoterapeits. Strādāju ar pacientiem pēc insulta astoņus gadus un tas ir bijis dažādos rehabilitācijas posmos gan akūtā posmā insulta vienībā, gan, šobrīd, subakūtā posmā, kur lielākā daļa pacientu ir pacienti pēc insulta. Tāpatās arī mājas rehabilitācijā, kur ir gan subakūti pacienti, gan arī ilgtermiņa posmā.

11 Mod: Paldies.

12 Dal5: Labvakar! Mani sauc Klēra. Es esmu fizioterapeits. Laikam līdzīgi kā iepriekšējiem diviem fizioterapeitiem, tie nav pamat pacienti, bet ikdienā tie ir un tie laikam būtu seši gadi, kuriem sastopies. Līdzīgi kā Agnesei tie ir gan ambulatori, gan tie ir mājas pacienti, gan esmu strādājusi rehabilitācijas centrā, kur tie uzturas stacionāri.

13 Mod: Paldies!

14 Mod: Palika vēl viens dalībnieks.

15 Dal6: Sorī, sorī, sorī. Es esmu te. Esmu ergoterapeits. Strādāju. Jāsaka kur? Strādāju veselības centrā un rehabilitācijas centrā. Rehabilitācijas centrā bija lielāka pieredze ar insulta pacientiem, šobrīd viņa ir diezgan mazāka, bet pieredze būtībā ir diezgan liela. Varētu teikt, ar dažāda neatkarības līmeņa pacientiem.

16 Mod: Paldies visiem. Pirmais tāds jautājums visiem ir - kā jūs komentētu plašsaziņas līdzekļos pieejamo informāciju par šo digitālo asistentu "Vigo"?

17 Dal5: Es varu nokomentēt šo, jo, manuprāt, ja jūs tiešām viņu gribat reklamēt. Tas vismaz ir tajā manā burbulī, šis te mārketings ļoti pietrūkst un cik es esmu jautājusi rehabilitācijas speciālistiem sev apkārt, viņi par šādu lietu nezin. Principā, es to esmu arī uzzinājusi tikai (personvārds) dēļ, kāpēc kaut kas tāds vispār ir. Iespējams, ja vairāk tā ikdienas dzīve ir rehabilitācijas centrā un, strādājot šajā rehabilitācijas centrā, visticamāk tur šie rehabilitācijas speciālisti to zina. Bet es teiktu, ka ārpus rehabilitācijas centra, vismaz manā informācijas burbulī par to nezina.

18 Dal2: Es varu tikai piekrist Dal5, ka es arī nezināju pirms tam par šādu aplikāciju, bet apskatot viņu, izlasot par viņu, es atcerējos, ka kaut kur tomēr biju padzirdējusi. Bet tā vairāk īsti nē.

19 Mod: Olga kaut ko gribēja komentēt.

20 Dal1: Jā, ta kā bija minēta darba vieta, tad mana darba vietā Vigo ir aktīvi pielietojama tehnoloģija. Bet tā, lai dzirdētu kaut ko ārpus, tad nebija nekad. Ja mēs, man tas priekšstats par to mājas lapu, ja es gribētu papētīt to mājaslapu, tad man liekas, ka tā informācija ir ļoti skopa. Nedod nekādu priekšstatu. Viņa ir ļoti tāda.. tā mājaslapa nav informatīva. Lai kaut kādā. ..

21 Mod: Priekš speciālistiem vai priekš pacientiem?

22 Dal1: Jā, arī priekš speciālistiem. Viņa nav tāda, ka tu paskatītos, izlasītu, uztvertu to visu un padomātu: "Jā, tas būtu nepieciešams tieši man vai manam radiniekam, vai pacientam."

23 Dal4: Es varētu pievienoties iepriekšējiem diviem dalībniekiem un teikt to, ka neesmu samērā daudz lasījusi tieši plašsaziņas līdzekļos. Vairāk tāpēc, ka mums uz darbā bija iespēja uz aprobācijas laiku to izmantot. Varbūt vienu ziņu es biju lasījusi, iespējams facebook lapā, Stradiņu kontā saistījumā ar pētījumu, kas bija pagājušo gadu izplatīts. Bet otra lieta, kas varētu būt kā indikators, kāpēc cilvēki neredz. Man bija pagājušo nedēļ 3. kursa studenti, ergoterapeiti, kur mums arī ir par insulta tēmu jāmācās. Par akūtu, par subakūtu insultu. Par novērtēšanu. Gribēju, lai studenti izlasa to publicēto rakstu, bet viņi arī neviens no 24 studentiem, kuri tagad aktīvi lasa daudz ko rehabilitācijā, nezināja par Vigo. Nebija neko redzējuši.

24 Dal3 : Tad es paturpināšu. Es arī iepriekš, līdz. Es neteiktu, ka es varbūt nezināju pilnībā, bet laikam tā informācija nebija tāda, lai mani viņš uzrunātu un es arī pirms tam būtu vairāk interesējusies par šo Vigo aplikāciju. Bet vairāk arī uzzināju tieši, kad uzrunāja par šo te pētījumu. Apskatot arī šo te gan facebook, gan arī.. Neskatījos vai jums ir instagram aplikācija, bet laikam arī tad nebija, jo neatradu īsti, man liekas. Piekritīšu, ka tā informācija, neatceros, kurš no dalībniekiem teica, ka tā informācija ir diezgan skopa. Nesasniedz tomēr tos cilvēkus, kurus varbūt vajadzētu sasniegt.

25 Mod: Paldies. Tālāk es gribētu jautāt par pašu lietotni. Kāds ir jūsu viedoklis par izglītojošās informācijas un vingrinājumu atbilstību insulta pacientu rehabilitācijas nolūkam?

26 Dal3: Es tad sākšu. Apskatot vingrinājumus, gan arī video un visus pārējos, viņi ir atbilstoši pacientiem pēc insulta. Galvenais ir tas atkarībā no kā, teiksim tā, kādi ir šo te pacientu funkcionālais stāvoklis. Tad mēs arī iepriekš. Es arī šobrīd apskatot, takā izmēģinot Vigo, es īsti nesaprotu kāds īsti ir šis pacientu funkcionālais stāvoklis, cik daudz viņš spēj. Attiecīgi, tad es saprotu, ka tas pēc tam tiek pielāgots. Tik cik es apskatījos tos dažādos vingrinājumus, man likās piemēroti insulta pacientiem.

27 Mod: Vai citiem ir viedoklis par vingrojumiem vai informatīviem materiāliem, kas ir lietotnē?

28 Dal4: Man varbūt iekrita acīs viens tāds aspekts saistībā ar roku vingrojumiem. Tur bija nepieciešams sākotnēji izmantot pudelīti, lai veiktu plaukstas deviāciju, radiāli un ulnāri, tad pēc bija nākamā sarežģītības pakāpe kaut kas smagāks. Ja es nemaldos, tad konservu bundža, katrā ziņā, es kā speciālists droši vien tāda veida aprīkojumu, neieteiktu pacientam. Varbūt ieteiktu to pudelīti iepildīt ar ūdeni, lai būtu kaut kāds apsmagojums, vai hanetlīti paņemtu. Tas bija tas, kas man, ja mēs tieši par vingrojumiem runājam, iekrita acīs.

29 Mod: Viens no jautājuma, mana uzdotā jautājuma, tādiem atslēgvārdiem, bija atbilstība insulta pacientiem. Ko jūs pratāt noteikt šo vingrojumu vai informācijas atbilstību tiem pacientiem?

30 Dal4: Tas jautājums ir, kurš to noteiks?

31 Mod: Mēs runājam tagad par to saturu, par vingrojumiem, kas ir lietotnē un par to informatīvo saturu. Jā. Jautājums par to, kas noteiks to, ka šie vingrojumi vai informācija būs vai ir atbilstoša? Jūs kā speciālisti no savas kompetences, strādājat ar šiem pacientiem, varat izteikt savu viedokli. Kas, jūsuprāt, būs atbilstoši un vai tie ir atbilstošie ? Pēc kā var spriest?

32 Dal4: Tas ir takā arī iepriekš jau dalībnieks teica. Tas ir atkarībā no tām problēmām, kas cilvēkam

būs un no tā problēmu apjoma. Tāpēc es saredzu, ka ir īstenībā svarīga, tāda pilnīga pacienta novērtēšana sākotnēji un tad būs, no speciālista viedokļa, būs saprotams vai tie vingrojumi līdzsvaram, rokām vai kājām būs viņam atbilstoši. Un tad pēc kāda atkārtota speciālista novērtēšana. Jo radinieki un pats pacients jau nespēs takā pareizi un objektīvi novērtēt vai viņam tas der. Viņam patiks, viņš vingros, viņš teiks: "Jā, tas man ir atbilstoši".

33 Mod: Principā par to saturu novērtējumu mēs arī runāsim tālāk. Es ierosinu arī turpināt šo tēmu, ko Dal4 ir atskaņojusi. Par to, kāds būtu, jūsuprāt, kāds būtu tas veiksmīgākais veids kā var pielāgot programmu atbilstoši pacienta vajadzībām? Vai ir citi varianti kā veikt pilnu novērtēšanu vai kas būtu tā pilnā novērtēšana, lai sastādītu to programmu?

34 Dal3: Manuprāt, tā būtu ar pacientu, vai nu tas ir, man liekas, kam būtu jābūt gan ergoterapeitam, gan fizioterapeitam, kurš ar pacientu tiekas klātienē nevis caur tiešsaisti sazinoties un izrunājot visas tēmas, kas ir nepieciešamas. Bet tiešām tiekoties klātienē. Manuprāt, es piekritīšu Dal4, ir jāveic šī te, pilna funkcionālā stāvokļa novērtēšana. Kas būtu svarīgākais, man liekas, ka būtu šim te gan ergoterapeitam, gan fizioterapeitam vai citiem nepieciešamiem funkcionāliem speciālistiem, uzturēt šo te kontaktu ar pacientu visas šī te. Arī, ja viņš izmanto šo te Vigo Health aplikāciju, tad arī uzturēt ar pacientu šo te kontaktu un tikties ar viņu atkārtoti. Lai arī nu takā, varētu šo te progresu, pielāgot šo te vingrinājumus tieši atbilstoši viņam nevis ļaut pacientam, tikai tad, kad mēs viņu pirmo reizi novērtējam, tad viņš sāk pats šo Vigo Health lietotni un mēs, viņš nezin vai viņam ir tas atbilstoši vairs vai nav. Bet varbūt pēc mēneša viņam šie te vingrojumi vairs neder.

35 Dal5: Es arī gribēju teikt, ka es piekrītu par to iepriekšējo, jo insulta pacientiem kā tādiem, vispār jebkuram pacientam, bet it sevišķi insulta pacientam. Atkarīgs, protams, no funkcionāliem traucējumiem, bet jo lielāki funkcionālie traucējumi, jo svarīgāks viņam ir atbalsts. Un es nerunāju pat par psihologa vai psihoterapeita atbalstu, bet arī šī te funkcionālā speciālista atbalstu. Ja tas ir vienkārši: "hey, rekur pildi vingrinājumu, bet es ar tevi nerunāju un tu vari ar mani sazināties čatā", tā nav pārāk personiska pieeja šim cilvēkam, tāpēc es godīgi varu teikt, man liekās, vispār šī aplikācijas lieta liekas ļoti interesanta. Vai tas vispār tiešām dzīvē, reāli, insulta pacientam ar lieliem funkcionāliem traucējumiem var strādāt? Jo viņš viens pats viņu pielietot noteikti nevarēs. Loģiski, tur būs jāiesaistās vai nu ģimenei vai nu tādā gadījumā aprūpes personai, fizioterapeitam, ergoterapeitam, kurš nāk uz mājām. Bet tas ir jautājums, vai ir jēga no šīs aplikācijas, jo tev jau re kur funkcionālais speciālists nāk uz mājām. Tāpēc es piekrītu arī par to, ka tā novērtēšana virtuāli, nu diemžēl viņa nebūs tik kvalitatīva kā novērtēšana dzīvē, noteikti. Un to, ka tas būtu nepieciešams to izdarīt vairākas reizes, tieši tā, lai šie te mērījumi un šis te būtu objektīvi, ne tikai subjektīvi no tā kādā veidā tas pacients jūtās.

36 Dal4: Es īstenībā gribu paturpināt Dal5 domu ar to, ko es jau domāju, ka īstenībā.. nu, redzot tiešām ļoti daudz insulta pacientus pa to manu praktizēšanas laiku, būs maza daļa pacientu, kam tas būs noderīgi un tiem, kuriem būs, tas tiešām būs ļoti jauki. Cilvēkam, kuram nav varbūt izteiktu kognitīvu traucējumi, nav afāzijas, nav jušanas traucējumi, nav kritienu risks, nav varbūt motivācijas trūkums un tā tālāk. Būs tiešām tādi līdzestīgi pacienti ar tādu varbūt vidēji vieglu parēzi un viņi varēs arī labi šo izmantot un labi arī sevi izglītot. Bet tiešām, es domāju, ka ir daudz pacientu, kuri vienkārši neiekļausies tajos iekļaušanas kritērijos, jo nespēs to lietot.

37 Mod: Paldies. Principā, man te sarakstīti jautājumi, kā jūs saprotat un mēs jau vienlaicīgi sākām runāt par gandrīz visiem no tiem. Es gribētu atgriezties pie tā piedāvājuma, ko atbalstīja daļa no grupas, ka veikt tādu kārtīgāku funkcionēšanas novērtēšanu, lai pārliecinātos, ka tie vigo piedāvātie vingrojumu būtu atbilstoši. Es gribētu attīstīt šo domu. Kā man to saprast, ka bez šīs novērtēšanas, Vigo lietošana, jūsuprāt, nebūtu iespējama? Vai arī kā Dal5 minēja, kāda jēga tad no tā Vigo, ja tas speciālists tāpat nāks mājās? Kur ir tas slieksnis, vai ir kaut kādi pacienti, kuriem varētu izmantot to tehnoloģiju bez šīs kārtīgākas novērtēšanas vai cik lielu, vai maza novērtēšana nepieciešama, lai iesāktu to lietot? Varbūt virzāmies šajā virzienā, lai saprastu. Citādi, es tagad saprotu, ka bez pilnīgas novērtēšanas, to nesanāks lietot.

38 Dal5: Tad turpinot to, ko Dal4 teica. Tas būtu iespējams cilvēkiem ar nelieliem funkcionāliem traucējumiem, nelielu parēzi un kuriem nav šie te, kaut kādi, tieši arī par šo, uztveres traucējumi un tā tālāk, lai tādā gadījumā viņš pats saprot arī ko viņš šajā aplikācijā dara un, ka šie traucējumi nav tik lieli, ka viņš var šo aplikāciju pats izmantot. Viņš izvēlās šo, jo viņam tas tiešām ir ērtāk nevis iet

pie kāda rehabilitācijas speciālista vai to, kas šis speciālists nāk uz mājām. Tas takā, manuprāt.

39 Dal3: Es priekritīšu Dal5 un arī Dal4, kura jau iesāka šo te teikt, ka takā tiešām tie būs ļoti, ļoti maza daļa. Lai gan mana pieredze nav tik liela, kā jums dažām citām dāmām, bet arī tā pacientu daļa būs diezgan maza, kuriem tiešām varētu tiešām takā izmantot. Un kurš varētu pieteikties šai te konsultācijai arī caur šo te mājaslapu un to varētu novērtēt arī caur internetu. Bet, manuprāt, tur vajadzētu diezgan lielu tādu apmācību kā vispār lietot šo te lietotni nevis tikai to novērtēšanu. Tam būtu jābūt takā tādam kopīgam darbam, man liekas, vai nu ar tuviniekiem vai tiešām ar kādu speciālistu.

40 Mod: Vai es varētu precizēt, kam vajadzētu apmācības? Pacientam, gala lietotājam, tuviniekam vai ?

41 Dal3: Pacientam, lai viņu apmācītu tiešām lietot. Jo ne visi pacienti, arī vecāka gada gājuma cilvēki, kuriem ir šie te insulti, viņi saprot vispār planšetdatorus, lietotnes un tā tālāk. Tas jau nav tā, ka mēs visiem varam iedot planšeti - ņem un rīkojies. Un viņš mācēs visu izdarīt. Tas, man liekas, ka arī diezgan liels tāds jautājums.

42 Mod: Tātad tā apmācība būs vajadzīga nevis saistībā ar insulta izraisītiem funkcionēšanas traucējumiem, bet ar to, ka cilvēks varbūt nespēs lietot šīs tehnoloģijas kā tādas?

43 Dal3: Jā.

44 Mod: Tātad, tagad izskanēja dažas tādas versijas, ka varbūt bez tādas kārtīgas novērtēšanas, varbūt nesanāks izmantot veiksmīgi. Varbūt sanāks izmantot veiksmīgi tiem, kuri ir diezgan funkcionāli neatkarīgi. Viegli pacienti, var nosaukt. Bet gribētu saprast, kā jūs saprotat šo pakalpojumu vai kā jūs redzat kā šis pakalpojums varētu tikt veikts? Vai tas pacients caur mājaslapu sazinās ar Vigo kompāniju, viņam tiek dota planšete. Vai iedota speciālista rehabilitācijas pakalpojuma rekomendē, apmāca? Kas ir šis brīdis un kā jūs redzat kā tas varētu būt? Jūsu sapratnē.

45 Dal4: Manurpāt, ka mēs jau te dalībnieki, kas šeit esam. Vismaz, es teikšu par sevi, es domāju, ka arī kā Dal5 teica, ka šis varētu būt tāds lielisks rīks un tāds jau nākamais solis tehnoloģiju izmantošana un tā. Tie varētu būt arī speciālisti, kas strādā. Pieņemsim, ja zin, ka akūtā posmā, ka pacients brauks mājās un var redzēt, ka tas pacients jau līdzestīgi sadarbojas terapijā. Un bez šiem, kā jau es pieminēju, jušanas vai viņš ir arī kritisks nevis nekritisks pret saviem traucējumiem. Un nav viņam neglekts vai viņš jau ir nākamajā posmā rehabilitācijas centrā. Var saprast, ka viņam tas varētu būt noderīgs. Arī tāds ceļš varētu būt labs. Kā rekomendācija, tikai tad būtu jābūt tādām skaidrām vadlīnijām atlasīt, kurus tad izslēgt no tiem un kurus iekļaut. Jo, es saku, tos, kurus es tikko pieminēju. Piemēram, pacients, kurš nav kritisks pret saviem traucējumiem vai ir nevērīgs pret savu parētisko pusi. Viņš tos vingrojumus darīs ar savu veselo pusi. Un arī, mums, piemēram, nodarbībās viņam ir nemitīgi jāuzsver, piemēram, paceļam kreiso roku, skatāmies uz kreiso roku. Un visu pusstundu, četrdesmit minūtes un, ja viņš sēdēs viens ar planšeti, tad diemžēl, viņš izdarīs pāris reizes un sēdēs priecīgs, ka viņam tas ir izdevies. Tāpēc es tur tādus riskus saredzu, ka lielai daļai tas diemžēl nederēs.

46 Mod: Es piedāvāju arī pārējiem pieslēgties turpināt šo tēmu par funkcionēšanas kā ieslēgšanas un izslēgšanas, iekļaušanas kritēriju tam pacientam vai viņa kaut kādiem citiem faktoriem. Kam tas derēs, kam tas nederēs un kam būs grūtības lietot šo ierīci?

47 Dal1: Mūsu darba vietā notiek aktīva Vigo ierīces piedāvājums, aktīvs piedāvājums mūsu pacientiem, teiksim tā. Un es neesmu viens no tiem speciālistiem, kas ar to nodarbojas, bet pārrunājot ar kolēģiem, kā tas notiek un kam tas ir noderīgs, noderīga ierīce un programmatūra. Kolēģi saka, ka tā atlase ir ļoti, ļoti sarežģīta. Ne visiem var to piedāvāt, jo bieži vien ir kognitīvie traucējumi. Un tas uzreiz ierobežo Vigo lietošanu. Tāpēc, ka jābūt kā jau meitenes, es atvainojos, kolēģe, pirms tam jau teica, ka ļoti svarīga ir kvalitatīva novērtēšana un arī tāpat ir ļoti svarīgs tieši starpvērtējums. Vai novērtētu, vai tas sanāk. Izvērtēt to, kas jau ir apgūts un vai tas iet uz priekšu. Un vai cilvēks to dara pareizi un vai varbūt ir kādi citi deficīti ietekmē Vigo lietošanu.

48 Dal4: Varbūt es tikko kamēr klausījos, ko Dal1 saka. Iedomājos tādu domu, piemēram, ka tajā brīdī, kad cilvēks ir rehabilitācijas iestādē, vienalga kurā. Un tur viņam uz vietas ir iespējams izmēģināt to savu, tās divas nedēļas, trīs nedēļas un tas var būt tas brīdis, kad saprotams vai viņam to var rekomendēt tālāk, jo tad jau var redzēt. Mums ir bijuši arī pacienti, kuram tas der un tad mēs viņam iedodam. Prasām: "Vai jūs vakardien darījāt?", "ai, nu es aizmigu, es aizgāju pastaigāties". Tad pēc tam "es ieslēdzu, nevarēju sagaidīt, kad tie vingrojumi beigsies". Takā varbūt pat pacients der, bet viņš tomēr tāpat nelieto kaut kādu citu iemeslu dēļ. Varbūt tāda veida atlase varētu būt noderīga. Takā izmēģināt jau, kā viņam iet ar planšeti, aplikāciju.

49 Dal5: Jā, es piekrītu ļoti Dal4 par to. Jo, man liekas, ka svarīgi ļoti ir vispār cilvēku motivācija. Šo te pacientu motivācija pašiem, vieniem pašiem to darīt. Jo, nu godīgi, arī es domāju, ka piekritīs kolēģes, cik praksē, strādājot ar cilvēkiem. Cilvēki, kas atnāk un saka, ka grib mājas programmas, nu 90- 95 % no viņiem tās mājas programmas pēc tam nepilda. Viņi vienkārši. Tāpēc, ka ir nepieciešama tā motivācija. Ir nepieciešama motivācija kaut ko darīt. Tāpēc arī lielākā daļa cilvēku iet kaut kur darīt vai viņi iet, ja mēs runājam arī par ne insulta pacientu, vai viņi iet uz treniņiem vai viņi iet pie kāda trenera, vai fizioterapeita. Līdzīgi jau būs arī insulta pacientiem. Viņiem jau arī ir jābūt tai motivācijai. Okay, mēs varam teikt, ka varbūt viņa motivācija ir atgriezt viņa kustības, samazināt viņu parēzi, bet tas ir tāds.. Tev ir jāmaina ļoti sava domāšana, lai tu vispārībā to gribētu. Tāpēc es piekrītu Dal4 to, kas ir, viens, ka, jā, varētu šiem te cilvēkiem, šo izmēģinājuma laiku dot. Vai viņam tas vispār der.

50 Dal4: Es klausījos Dal4 un atcerējos vienu savu tādu domu, kas man bija nosēdusies, ka cilvēkiem pārsvarā ir tāda sajūta, ka rehabilitācija ir pasīva. Ne tikai saistībā ar insultu, bet arī citām saslimšanām. Ka viņš saņems, piemēram, masāžu vai procedūras vai kaut ko un tas viņam atvieglos. Tiešām tā aktīvā daļa ir mazāk un varbūt tas tiešām nāk tieši no pagājušā gadsimta, kad tiešām tās visas metodes arī bija pasīvas. Kad cilvēki brauca uz kūrorta rehabilitācijas vai sanatorijas centru un visi šādas pasīvas metodes saņēma. Un tie arī ir cilvēki gados. Protams, es neapgalvoju, par visiem. Ir arī cilvēki, kas aktīvi iesaistās, bet šī arī varētu būt problēma, vispār takā cilvēkiem domāšanā.

51 Dal3: Jā, es laikam apstiprināšu to Dal4 un Dal5 domu, ka takā būtu ļoti labi, ja patiešām pacienti varētu izmēģināt. Un arī kā funkcionālais speciālists varētu novērtēt kā šis pacients, vai viņš tiešām var viņu izmantot. Vai viņam viņš ir ieteicams mājās turpināt. Tos pašos rehabilitācijas centros, kur pacienti atrodas uz vietas. Nezinu, piemēram, piedāvāt viņam izsniegt, nezinu, un uz vietas izmēģināt vairākas dienas pēc kārtas un arī novērtēt to.

52 Mod: Runājot par tiem iekļaušanas, izslēgšanas kritērijiem, pacienta funkcionēšanu. Vēl papildus jautājumus. Kāds būtu, vai var spriest pēc laika rāmja, kad tas pacients būtu gatavs lietot šo Vigo terapiju? Uzreiz pēc slimnīcas, akūtas rehabilitācijas vai tajā brīdī, vai subakūtajā posmā vai starp rehabilitācijām?

53 Dal3: Man liekas, ka tas ir ļoti individuāli ar katru pacientu skatāms. Mēs nevaram pateikt, tāpēc, ka tagad beidzās akūtais posms un viņš jau var sākt, bet cits, kuram beidzas akūtais posms, viņš galīgi nebūs gatavs uzsākt šādu aplikācijas veikšanu. Tāpēc tur atkal ir jānovērtē, individuāli, vai tas pacients būs spējīgs vai viņš nebūs spējīgs.

54 Dal4: Viņš varbūt arī savā, šajā ilgtermiņa, hroniskajā posmā nebūs vispār nekad gatavs un spējīgs to lietot. Cits, savukārt, jau akūtajā posmā jau var uzsākt un lietot un turpināt. Takā es domāju, ka tieši tas laika konteksts šeit nav noteicošais.

55 Mod: Tad mēs sākām runāt par saturu, par vingrojumiem un to individuālo programmu un nonācām pie tā, ka vajadzētu būt novērtēšanai, lai programmu izveidotu atbilstošu pacienta vajadzībām. Kā jūs saskatāt, kā varētu tikt veikta šī novērtēšana? Vai tas obligāti speciālistam, vai pacients pats par sevi kaut ko atzīmēt? Vai tas jābūt tuvinieks? Vai tas jābūt rehabilitācijas pakalpojuma obligāti iekļauts vai tas var eksistēt ārpus rehabilitācijas, kā atsevišķa ierīce?

56 Dal1: Mans uzskats ir, ka tiešām jābūt rehabilitācijas speciālistam, kas strādā šajā jomā. Jo mēs zinām, ka ir tādi gadījumi, kad radinieki spēj novērtēt, saka, ka mans vīrs ir pilnīgā guļošs, viņš neēd, viņš neko nedara. Bet reāli tie funkcionēšanas traucējumi nav tik smagi šiem pacientiem un viņš spēj

sevi aprūpēt. Tāpēc es uzskatu, ka tomēr jābūt speciālistam, kas spēj kvalitatīvi veikt pacienta novērtēšanu.

57 Dal3: Jā, arī pats pacients reizēm nenovērtē sevi takā, tieši tā kā viņa stāvoklis ir. Viņš varbūt vērtē pārāk labi vai varbūt vērtē pārāk slikti. Takā tas arī nebūtu tas uz ko varētu pamatoties un es piekritīšu Dal1, ka būtu jānovērtē tieši rehabilitācijas speciālistam un funkcionālajam speciālistam.

58 Dal4: Es arī piekrītu, tam, ka ne pacients, ne pacienta tuvinieki nav spējīgi objektīvi novērtēt. Arī radinieki var būt tādi, kas pārāk labi novērtē pacienta stāvokli. Piemēram, viņam ir afāzija un tiešām, globāla afāzija un viņš nesaprot. Nē, viņš mums saka "Jā, jā, jā, jā". Viņš paēda un vai - jā, jā, jā, jā - viņš nemaz nepaēda. Tur nav svarīgi vai tie ir speciālisti rehabilitācijas centrā, kuriem ir pieeja Vigo. Vai tie ir speciālisti, kas ir tieši no Vigo kompānijas, respektīvi, ka tiem ir jābūt speciālistiem neirorehabilitācijas jomā.

59 Mod: Labi, es gribētu tālāk turpināt runāt par šo tēmu, bet es to nedaudz paplašināšu. Runāsim par iespējamiem ieguvumumiem vai iespējamiem trūkumiem, izmantojot Vigo mājas rehabilitācijā. Es gribētu paturpināt iepriekš teikto, ka pacients vai tuvinieks var sevi pārvērtēt vai neatbilstoši zemu novērtēt savas spējas. Kādi būtu, piemēram, trūkumi no šī scenārija, ka viņam iedod planšeti un viņš nepareizi izvērtē savas spējas. Kādas būs sekas iespējamas?

60 Dal3: Manuprāt, viens no tiem tiem trūkumiem tas, ka viņš izmanto šo te planšeti, bet viņam nav, piemēram, varbūt cilvēks nav blakus. Pirmais varētu būt kritienu risks.Pacients neizvērtē tomēr vai viņš var to vingrinājumu veikt vai nē. Man liekas, ka tas ir primārais. Tad arī tas bīstamākais būtu šis te kritienu risks. Varbūt pārējiem kolēģiem ir ko pieminēt vēl

61 Dal4: Kritienu risks kā viens no faktoriem. Cits var būt arī tas, ka arī nekāds ieguvums nav. Vēl kāds ieguvums, varbūt, kā es esmu novērojusi pacienti veic vingrojumus, piemēram, ja viņam praktiski aktīvu kustību rokā nav. Tad viņš ar savu veselo roku varbūt tiešām ļoti spēcīgi veic pasīvas kustības un es nezinu, var teikt, lauž sev pirkstu ārā vai ļoti strauji veic šīs kustības un veicina kaut kādu plecu locītavas subluksāciju. Kas arī takā planšetē skatoties, arī varbūt tādu aspektus nepamanīju. Saistībā tur ar roksas pozicionēšanu vai plecu somas lietošanu. Tādus riskus es saskatu.

62 Mod: Es precizēšu. Pareizi saprati, ka viņš veic šīs kustības, tāpēc, ka viņam traucēta jušana un viņš nav kritisks pret to, ka viņš nejūt, piemēram, otru roku un tāds iznākums.

63 Dal4: Nē, tas var būt arī pacientiem bez jušanas traucējumiem un arī kritiskiem pacientiem. Vienkārši viņš tādā veidā veic sev pasīvas kustības. Ļoti lielu amplitūdu, ļoti lielu spēku. Tā es to domāju.

64 Dal5: Es piekritīšu arī, jo, manurpāt, galvenais, kas ir par to, ka pacients var traumēt sevi. Un dažādos veidos. Tāpēc, jā, tad tas būtu tas galvenais.

65 Mod: Tātad tika identificēti trūkumi pie nepareizas novērtēšanas un programmas sastādīšanas, bet kādi būtu potenciālie ieguvumi, ja pacients lietotu mājas vidē šo rehabilitācijas metodi, šo terapiju? Jūs varat domāt par dažādiem scenārijiem. Vai viņš lieto patstāvīgi un viss kārtībā. Vai viņš lieto ar speciālista asistēšanu, kurš viņu nokonfigurētu to mājas vidē vai vēl kaut kā. Tātad, kādi scenāriji jums nāk galvā, kādi var būt ieguvumi?

66 Dal2: Man liekas, ka galvenokārt jau tas, ka viņš kaut ko dara mājās. Jo tas, ka viņam tā motivācija ir. Tas, ka viņš vismaz kustās un vinš vismaz kaut ko dara.Tas ir tiem pacientiem, kas tiešām to izmanto un tiešām to veic, tad viņiem ir dažādi vingrinājumi, kurus viņi var veikt. Ja viņi tiešām viņus pilda, tad viņiem ir arī, ja ir atbilstoši novērtēts un atbilstoši vingrinājumi, tad viņi var arī panākt to uzlabojumu sev.

67 Dal4: Es piekrītu arī, jā. Ka tas var palielināt pacientu līdzestību rehabilitācijas procesā. Uzlabot arī viņa funckionālo stāvokli, neatkarību ikdienā. Attiecīgi, ja viņš paliek fiziski kustīgāks un spēcīgāks, mazinās iespējams arī aprūpes līmenis, kas radiniekiem ir jāsniedz vai jāasistē. Tas mazina arī izmaksas varbūt par laiku, par kaut kādiem ceļa izdevumiem, ko viņš pavadītu braucot. Tas iespējams arī mazina to, ka pacienti perifērijā, kuriem nemaz tuvumā nav šādas iespējas uz

nodarbībām braukt, arī tas ir kā risinājums.

68 Dal3: Jā, es laikam piekritīšu tam šim te pēdējam par pacienta šim te izmaksām. Ka iespējams, es protams nezinu, arī mājaslapā nebija takā noteikts kādas ir aptuvenās izmaksas šim te pakalpojumam, ka tas arī bija ierakstīts, ka tas ir individuāli. Bet, ka tieši tās papildus izmaksas un arī laiks, ko pacients pavada varbūt ceļā un varbūt kādam citam tuviniekam viņu kaut kur ir jāved. Vai arī, jā, tas būtu tāds viens labs iegums, ļoti labs. Protams, attiecībā vai viņš viņu pilda, ko es piekritīšu Dal2. Tur atkal ir jāskatās.

69 Dal6: Zin kā, tīri no manas pieredzes. Jūs mani dzirdat? Tīri no manas pieredzes, vairāk tas, ka cilvēki prasa ko darīt mājās un šitas varētu būt veids kā viņiem demonstratīvi parādīt to, ko darīt. Jo parasti ir tā pārnese grūtāka no tās terapijas uz to mājas vidi, bez kaut kādiem papildus šāda veida. Nu, tādā ziņā tā ir laba ideja, bet nu jā. Man liekas, ka tīri tas, ka cilvēkiem vajag zināt ko darīt mājās.

70 Mod: Sakiet, lūdzu, kā jūs vērtētu šīs ierīces un programmatūras saprotamību, lietošanas vieglumu pacientam?

71 Dal4: Grūti jau pateikt, izejot tikai no savas perspektīvas, ar savām smadzenēm un uztveri. Katrs pacients jau ir citādāks, tur jau jums ir pētījums par viņu pieredzi lietojot. Bet varbūt es paturpināšu, tā kā es mēģināju lietot, man varbūt traucē tas, ka es nevaru brīvi pārvietoties pa tām tēmām un mani vadā tikai tas algoritms, kur man ir obligāti jānoskatās, obligāti uz visu jāatbild. Es, piemēram, gribēju atrast informāciju par tehniskiem palīglīdzekļiem un mājas vides pielāgošanu. Man varbūt prasītos tāds meklētājs, kur es varētu sameklēt. To es tur neatradu un tāpēc, man, var teikt, ka tas manu būtību bremzē tieši tas algoritms.

72 Dal3: Jā, es varbūt paturpināšu arī šo te. Ka man bija interesanti tas, ka viņa ir takā sarunas veidā, bet, piemēram, ieejot. Nezinu vai tas ir takā no pacienta skatpunkta, vai viņš tieši tāpat kā mēs redzam, kad izmēģinām. Tad pazūd šī te iepriekšējā informācija, ko takā šis te, ko lietotne sniedz. Piemēram, es vakardien skatījos un man stāstīja tas Vigo par miega kvalitāti, par miegu, par visu to. Bet šobrīd, es piemēram atvēru atkal no jauna, viņš lika ielogoties no jauna un es vairs neredzu šo te informāciju, kuru es, piemēram, varētu izlasīt. Varbūt es vakar lasīju un vairs neatceros un šodien gribētu paskatīties. Vai arī, tāpat kā Dal4 teica, kaut ko pameklēt par tehniskajiem palīglīdzekļiem, ko es varētu uzzināt jaunu. Tad nav šī te vieta, kur es varētu paskatīties. Vai arī es vienkārši varbūt neatradu. Tas takā no mana viedokļa, ko es par pašu lietotni. Un es piekritīšu arī par to video, ka, tad, kad skatās, nevar īsti viņu patīt uz priekšu ne atpakaļ, lai kaut ko atkārtotu vai kā. Tad tev jānoskatās pilnīgi viss līdz galam, lai tu tiktu arī tālāk.

73 Mod: Vēl varētu salīdzināt šo Vigo programmu, kur ir vingrojumi, informatīvie materiāli, ar to individuālo programmu, ko rehabilitācijas speciālists bieži papīrā izdod saviem pacientiem. Vai Vigo ir priekšrocības un trūkumi, salīdzinot vienkārši ar papīra materiāliem, ko, piemēram, speciālists dod pacientam?

74 Dal4: Priekšrocība varētu būt tā, ka droši vien planšeti pasaudzēs un nepazaudēs kā tas varbūt citreiz ir ar izprintētiem materiāliem, kas visā dokumentu jūrā kaut kur pazūd. Arī tas, ka uzzīmēt tieši to kustību, kas ir jāveic, nereti arī ir grūtāk, izaicinošāk, vismaz man. Ja planšetē, tas ir video veidā, tad tas uzreiz ir saprotami parādīts. Un man īstenībā kopumā saistībā ar dažādiem materiāliem insulta rehabilitācijas jomā, mulsina, kāpēc katra iestāde vai nezinu firma, vai vēl kas, taisa savus materiālus. Es, piemēram, būtu par tādu reporizotoriju, kur viss ir apkopots. Nu, visiem vienāds. Nevis, piemēram, viena slimnīca taisa vienu bukletu un otra slimnīca taisa citu bukletu un citi vēl iztulko, farmācijas firma, citu. Tad šajā var vienkārši apmienot šo visus, gan vingrojumus, gan. Es vispār saredzētu arī, lai tas derētu tādiem funkcionāli smagākiem pacientiem. Varbūt, ne tikai priekš pacientiem tas rīks, bet arī radiniekiem. Kā aprūpes aspekti varētu būt gan, nezinu, pozicionēšanas vai pasīvu kustību veikšanas vai vairāk tādas asistēšanas ikdienā. Es varbūt arī tādu lietu saredzētu. Takā tāds digitālais buklets.

75 Dal3: Es piekritīšu Dal4 par šo te, ka papīrs tomēr ir papīrs. Mēs viņu varam vainu pazaudēt vai arī nejauši izdarīt, nezinu, apliet vai saplēst vai kas notiek. Bet tomēr šis digitālais rīks, tur diez vai mēs kaut ko tādu varētu izdarīt. Tas būs pieejams visu, visu laiku. Tāda takā ļoti laba priekšrocība,

manuprāt. Un arī, piekritīšu par to aprūpes, par to kā aprūpēt šo te pacientu. Tuviniekiem būtu ļoti labs, ko iekļaut vēl arī šajā te Vigo, varbūt arī turpmāk.

76 Dal2: Es piekritīšu visam, kas iepriekš teikts, bet vēl piebildīšu to, ka pozitīvais faktors Vigo ir tas, ka tu tur vari vingrot kopā ar to speciālistu, kas vingro video. Tu to vari darīt kopā ar to video un tur takā ir tā līdzestība.

77 Mod: Labi, paldies. Es gribētu ierosināt runāt nedaudz par mērķiem un nolūku. No vienas puses varētu jautāt par to kā jūs vērtējat šī rīka atbilstību pasludinātajam mērķim palīdzēt insultu rehabilitācijā, bet no otra puses mēs varam vienkārši diskutēt kāds būtu tas mērķis. Vai šī instrumenta izmantošanas nolūks? Kā jūs redzat, kā to var izmantot, kādos scenārijos?

78 Dal1: Varbūt tāds viens no mērķiem varētu būt arī aktīvam savā mājas vidē. Tas Vigo varētu nodrošināt pakalpojuma pēctecību. Bet atkal, man visu laiku griežas galvā tas jautājums par to, cik tie vingrinājumi, tie ieteikumi, būs izpildīti kvalitatīvi. Paldies.

79 Mod: Varbūt uzreiz es jautāšu pretī - vai ir kaut kādi apstākļi, kas ļautu pārliecināties par to vai viņi tiks izpildīti kvalitatīvi?

80 Dal5: Manuprāt, nē. Bet, vienīgais ko, ja pievieno klāt, piemēram, iespēju, ka viņš pats var sevi filmēt, tādā veidā viņš izpilda un tas rehabilitācija speciālists var novērtēt, cik kvalitatīvi, nekvalitatīvi tas ir izpildīts. Tā, man liekas, ka tā ir vienīgā iespēja kādā veidā to var, nu, kā to var redzēt un saprast.

81 Dal4: Vēl jau varētu būt tāda sarežģītāka versija, tikai es nezinu vai tas ir reāli iespējams. Ja viņam kaut kādās konkrētās ķermeņa vietās, kas sākumā tiek nokalibrēts, tiek pielikti marķieri. Piemēram, tur pie pleca, elkoņa, plaukstas un tad viņš tiek antropometriski nomērīts un tad izejot no tā var skatīties to kustības amplitūdu, ātrumu vai kvalitāti. Bet nu, es nezinu vai tas tehniski iespējams.

82 Mod: Der visas idejas. Jūs varat piedāvāt principā jebko. Piemēram, atkal salīdzinām ar konvenciālu veidu, ka iedod norādījumus terapijai mājās. Speciālists pastrādāja ar pacientu, iedeva viņam, piemēram, papīra materiālus, vingrojumu kompleksu. Kas šajā gadījumā varētu palīdzēt šim pacientam pareizāk izpildīt šos vingrojumus? Varbūt ko jūs iedomāsieties, būs tas pats priekš Vigo.

83 Dal4: Tas ir, ja es to pacientu redzu divas nedēļas un mēs veicam šādus vingrojumus, tad tos pašus, ko viņš jau ir iemācījies, tas par ko es esmu pārliecinājusies, to es viņam iedodu. Un, nu es ticu viņam vai uzlieku to atbildību viņam, ka viņš to arī turpinās darīt.

84 Mod: Labi, vai ir kaut kādas domas par šīs lietotnes mērķiem vai atbilstību tiem mērķiem?

85 Dal4: Vai var vēlreiz atkārtot mērķi?

86 Mod: Principā, es nezinu kā mērķis tiek definēts, bet šodien mēs runājam par jūsu viedokli, par to kā var izmantot šo rīku mājas rehabilitācijas kontekstā.

87 Dal3: Takā mums būtu jānosaka tas mērķis? Vai viņš jau ir noteikts un mums kaut kāda atbilstība? Es laikam arī neizpratu.

88 Mod: Tāpēc es saku, ka nav tāda mērķa, ko es varu jums definēt. Bet es varu definēt to izmantošanas nolūku. Šī instrumenta izmantošana insulta pacienta rehabilitācijai mājās. Tātad, mēs mēģinām saprast kā var izmantot un vai vispār var izmantot, vai šis rīks būtu atbilstošs, lai pacienti mājās izmantotu to terapijas nolūkiem.

89 Dal6: Es drīkstu ātri? Es arī rakstīju pirms tam, ka man kopumā par to aplikāciju ir tāds duāls sajūtas. Jo no vienas puses liekas, ka viņa ir taisīta tādiem praktiski neatkarīgiem cilvēkiem pēc insulta, bet tie vingrinājumi tajā pat laikā liekas tādi ļoti.. vienkāršoti, ļoti primitīvi. Kuras prasmes šie neatkarīgie cilvēki jau ir sen ir apguvuši. Tad man liekas, ka tā aplikācija īsti nespēlē to savu lomu tajā cilvēku aktivizēšanā. Cik es esmu sapratusi, tas Vigo galvenais mērķis ir aktivizēt šos cilvēkus pēc insulta mājās, lai tā nezinu, institūcija apmeklēšana, visādu nodarbību apmeklēšana, nebūtu tik

ļoti nepieciešama. Lai cilvēks varētu mierīgi mājās, savā vidē darīt to, kas viņam ir jādara, lai varētu uzlabotu savu veselību. Es vienkārši saku, ka man ir tādas duālas sajūtas par šo aplikāciju. Līdz ar to tas mērķis arī liekās. Es saprotu, ka mēs gribam neatkarīgu to cilvēku, bet tā aplikācija īsti neļauj cilvēkiem, kas ir ar lielāku atkarības līmeni, nu nav viņa īsti piemērota cilvēkiem, kuriem ir vēl kaut kādi kognitīvi vēl stāvokļi blakus tam pašam insultam, jo ļoti bieži ir, varbūt šie ļoti fiziski spējīgie cilvēki tieši kognitīvi ir vājāki. Ja var saprast ko es ar to domāju.

90 Dal5: Es piekrītu Dal6. Es vēl gribēju piemetināt vēl to, ka, ja mēs vienkārši nostādām kā mērķi, ka mēs šo dodam vienkārši kā instrumentu insulta pacientiem, lai viņiem ir ar ko strādāt, tas nav mērķis, kas kaut ko sasniedz. Mēs vienkārši tādā gadījumā izveidojam programmu, lai cilvēki ņemtu un kaut ko darītu. Mērķis drīzāk būtu tādā gadījumā - mēs izveidojam šo programmu, lai uzlabotu esošo stāvokli un kaut kādu nākamo funkcionālo attīstības brīdi. Bet jautājums, priekš manis, atklāts paliek, to vai šī aplikācija spēs to izdarīt šim cilvēkam.

91 Dal4: Es piekrītu Dal5 par to, ka rehabilitācija kopumā, kā varbūt citi uztver dažreiz. Ka rehabilitācija ir tāda padarbošanās, vienkārši, lai cilvēks ir nodarbināts. Tas ir par kaut kādu konkrētu mērķi, izmantot metodes, kuras strādā, kuras ir pierādījumos balstītās. Tā kā Vigo vēl pagaidām ir šajā procesā, pierādījumu iegūšanu, tad es saskatu pirmo lielo svarīgo uzdevumu - veikt šo algoritmu kā atlasīt tos pacientus, kuriem tas derēs. Un tad tiem pacientiem es domāju, ka to uzlabojumu gan varēs sagaidīt, bet tā būs maza pacientu daļa. Tā kā, man liekas, ka tas mērķis, kas izskanēja vienā brīdī - kā palīgs vai kā atbalsts mājās, tas ir tāds nekonkrēts un prasa varbūt nedaudz tādu papildinājumu tajā formulējumā. Lai tas neizklausās vienkārši kā tāda padarbošanās.

92 Mod: Paldies, es gribētu pieķerties tai domai, kura ir izteikta par to, ka vajag atlasīt pacientus, kuriem tas Vigo der. Vai jūs pieļaujat vai redzat citu iespēju, vai nu pielāgot šo procesu vai pašu programmu, to saturu un funkcijas, lai tā derētu plašākai auditorijai, plašākam pacientu klāstam? Kaut kas uzlabojams, maināms, pielāgojams, lai tas derētu lielākam pacientu skaitam?

93 Dal4: Man liekas, ka izskanēja, arī Dal3 laikam teica un es arī teicu par to, ka smagākiem pacientiem tie var būt aprūpes principi vai cilvēki, kuriem ir tikai kognitīvi traucējumi. Varbūt tas ir priekš radiniekiem ieteikumi, kā organizēt mājas vidi, kā palīdzēt, kā komunicēt un tā tālāk. Respektīvi, iekļaut vairāk informācijas aspektu šajā aplikācijā un vērst to tikai ne uz pašu pacientu, bet arī priekš ģimenes izmantojamu rīku un informācijas avotu.

94 Dal3: Jā, es piekritīšu šim, ko Dal4 teica un arī, ko mēs iepriekš teicām. Ka vairāk varbūt tiešām vairāk uztaisīt takā divās daļās, vai tas pacients pats var jau var rīkoties vai viņa tuvinieki spēj takā palīdzēt viņam. Tad mazāk citādāk veidot šo te aplikāciju. Varbūt arī, ka mēs šo testa daļu, neredzam, es nezinu vai tas ir iekļauts vai nē, bet takā man tā doma būtu, ka tad, kad viņu atver, jau pašā sākumā tev ir iespēja uzzināt kā lietot šo lietotni, kā tev jādara, ko spiest, ko nespiest, lai tu kaut ko atvērtu. Tā kā tādu informatīvu materiālu vairāk sākuma daļā, lai iepazītos ar to pašu lietotni. Kur tu vari atrast kādu informāciju vai kur meklēt, kur pievienot. Tādā veidā. Kaut kādus ietiekumus arī vairāk.

95 Mod: Paldies. Vēl tāds jautājums - vai jūs uzskatās, ka speciālistiem, kas darbotos ar šo Vigo tehnoloģiju un iespējams ieteiktu pacientiem izmantot, ar pacientiem, būtu nepieciešama apmācība? Kādai jābūt tai apmācībai priekš speciālistiem?

96 Dal3: Manuprāt, viņai ir noteikti jābūt, lai mēs, lai, piemēram, es varbūt runāšu no sevis, ka, lai es kā funkcionālais speciālists spētu tam pacientam nodot pašu noderīgāko informāciju un pašu svarīgāko. Visu, lai viņš spētu izmantot to pēc iespējas vairāk. Jo tikai apskatot no sava viedokļa, es nevaru, piemēram, uzzināt daudzas lietas, kuras es nemaz nezinu par šo te aplikāciju. Tāpēc, manuprāt, tās apmācības būtu ļoti svarīgas, bet par ko tieši, tas būtu vairāk jādomā šiem te izstrādātājiem un to kādu informāciju būtu mums svarīgi zināt.

97 Mod: Vai es pareizi sapratu, ka pārējiem neuzskata, ka būtu nepieciešama šī apmācība vai vienkārši nav domas par to kādai tai jābūt?

98 Dal4: Nē, tu nepareizi saprati. Mēs vienkārši piekrītam, ka droši vien, aplikācijas izveidotāji un visa darba grupa jau zin visu pilnīgāk, to visu saturu, kas tur ir iekšā. Un es ar savu ātro dabu nevaru

sagaidīt visus video un visu, kad es varēšu ātrāk visu apskatīties un es vienkārši metu mieru tai darbībai un droši vien man vajadzētu apmācību. Man arī kā daļai varbūt no speciālistu loka, derētu tieši apmācība par to visu ieguvumu, ko pacients no tā var iegūt.

99 Dal3: Varbūt arī par to pašu procesu kā pacients to var iegūt, kā pieteikties un visu to. Tas arī būtu ļoti būtiski. Kā mēs, kā funkcionālie speciālisti, varam novirzīt šo te pacientu, lai viņš saņemtu adekvātu šo te palīdzību arī no Vigo Health un pārējiem.

100 Mod: Paldies. Mēs jau tuvojamies mūsu diskusijas noslēgumam. Tātad mēs runājām šodien par saturu, par programmas funkcionalitāti, par pacientu atlasi, par to kādiem pacientiem tā programma būtu derīga. Par to, kādi varbūt apstākļi tam pašam pacientam, par speciālistu iesaisti. Dažādas tēmas. Tagad beigās, varbūt jums ir domas, ka mēs kaut ko palaidām garām, vai gribas piebilst kaut ko saistībā par Vigo? Varbūt mēs kaut ko palaidām garām?

101 Dal4: Es nezinu, vai īsti neapguvu līdz galam vai tur ir tāda funkcija kā uzstādīt atgādinājumus, piemēram, ievadīt visus medikamentus, kas pacientam ir jālieto pa laikiem. Un, ja nu pacientam ir grūtības noorientēties tajos daudzajos medikamentos, vai viņam tomēr ir atmiņas grūtības. Vai planšete pati viņam, nevis sagaida, kad viņš sāks komunicēt, bet pati dod, nezinu kaut kādu atgādinājumu ar skaņas signālu vai tur izlec medikamenta nosaukums un tā tālāka. Vai atgādinājums, ka ir nepieciešams vingrot. Takā, ka tā planšete, varbūt tā aplikācija pati proaktīvi darbojās uz pacienta darbību. Nu jā, uz tiem vingrojumiem, uz medikamentiem, varbūt arī uz asinsspiediena mērījumu. Tādu funkciju es laikam nepamanīju.

102 Dal3: Jā, es arī riktīgi teiktu, ka šī būtu laba ideja. Man liekas, ka vēl viena laba ideja būtu tāda, ka gan pacients redz no sava profila, gan arī viņa funkcionālais speciālists var redzēt ko viņš ir izdarījis. Labi, viņš protams neredzēs kā viņš to ir izdarījis, ja. Par to mēs jau diskutējām. Bet, ka, piemēram, es kā funkcionālais speciālists, varu pieslēgties pacienta profilam un varu redzēt, ka viņš šodien ir pildījis tos, tos vingrinājumus. Piemēram, arī to pašu progresu, vai viņš vispār ir bijis iekšā vai nav bijis iekšā. Tā arī varētu būt tāda noderīga lieta.

103 Dal4: Vēl varbūt būtu noderīgi, ja tas dienas plāns arī būtu, nu iespēja, pašam to izveidot un modificēt. Jo mēs nereti arī kādam pacientam, kuram motivācijas grūtības vai grūti plānot, organizēt darbu. Mēs priekš viņa arī papīra formātā sastādām, piemēram, tur, astoņos ir sakopšanās. Deviņos brokastis. Desmitos vingrošana. Ja tas dienas plāns veidotos ne tikai no tā, ko viņš tur ir noskatījies, bet būtu arī manuāli aizpildāms. Ka viņš katru dienu redz to dienas plānojumu, priekš viņa individuāli saplānoto.
