# Supplementary material for: Use of the Digital Assistant Vigo in the Home Environment for Stroke Recovery: Focus Group Discussion With Specialists Working in Neurorehabilitation
Source: JMIR Rehabil Assist Technol. 2023 Apr 14;10:e44285. doi: 10.2196/44285 (PMC10148207; doi:10.2196/44285)
Supplement: Multimedia Appendix 4 [file rehab_v10i1e44285_app4.pdf]

Focus group protocol

Hello! Thank you for taking the time to participate in this focus group!

My name is Alexander Grjadovoy, I am an occupational therapist. The purpose of this focus group is to gather information for a study on the use of the digital assistant *Vigo Health* for the rehabilitation of stroke patients in the home environment. The planned duration of the focus group is 70 to 80 minutes.

[Focus group objective]

The main purpose of focus groups is to learn about the participants' views and opinions on the topic of discussion, so you are the main experts and there are no right or wrong answers to the questions. My role as moderator is to keep the discussion calm and help you to express your views.

I would stress that we do not have to agree on anything or take decisions on the topic of discussion. So feel free to express your thoughts even when they do not agree with others.

[Focus group rules]

Another very important thing is that we will try to make sure that only one person speaks at a time. This is so that when transcribing the recording, what you say can be heard and is not lost.

As we are meeting remotely, please manage your own microphones so that any noise does not interfere with others speaking and everyone can hear what is being said. I would very much like to ask everyone to keep their cameras on during the whole conversation.

[Confidentiality and informed consent]

The confidentiality rules for the focus group were sent to you in advance. As a researcher, it is my job to ensure your anonymity and the confidentiality of your data, so I will briefly explain the considerations involved.

Firstly, a video and audio recording will be made of the conversation, which will only be used for the purposes of the study, in order to transcribe the conversation for further analysis.

In the transcription, your names, titles, places of work, etc., which might allow you to identify the participants, will be replaced. Direct quotations may be used in the results of the study, so the possibility of being able to recognise someone by what they say cannot be excluded.

By agreeing to participate in a focus group, you also agree not to disclose the content of the focus group discussion in order to protect the confidentiality of the other participants.

[Moment for technical questions]

!!! [Start recording session] !!!

By taking part in this discussion, you acknowledge that you have read and agree to the rules of the focus group, as previously sent and now being played. If you have any doubts about your participation in this focus group, I will ask you to leave the group before we move on.

Focus group questions

Introductory questions

Let's start by getting to know each other.

1. Please tell us about the rehabilitation profession you are in and your experience in stroke rehabilitation?
2. How would you comment on the media coverage of the Vigo digital assistant?
   area of interest: education, length of service, types of service related to the rehabilitation sector

Key questions

3. What is your opinion on the relevance of educational information and exercises for the rehabilitation of stroke patients?
   Domain of interest: assessment of content, relevance of content to purpose.

4. What do you think would be the most successful way to tailor the programme to the patient's needs?
   area of interest: relevance to the patient's needs, treatment planning process.

5. How do you make sure that the treatment programme is appropriate for the patient?
   area of interest: relevance to the patient's needs, monitoring of the treatment plan/process.

6. What functional level would be appropriate for this therapy? What would be the time frame, after a stroke, when it could be used?
   Domain of interest: characteristics of the target audience, availability of use.

7. What do you see as the advantages and disadvantages of using a digital assistant in the home environment for stroke patients?
   Domain of interest: advantages and disadvantages of use, facilitators and barriers to use.

8. ~~How do you rate the user-friendliness of a digital assistant?~~
   Area of interest: accessibility of use.

9. What kind of training would be appropriate to educate professionals on the use and content of the software?
   Domain of interest: characteristics of the training, competences of the professionals, needs of the professionals.

10. After reading the available information and trying out the app, how do you assess its suitability for the purpose described?
    Area of interest: relevance of the app to its purpose, quality of the material available.

Closing questions

11. Is there something we missed that you would like to add about the use of the digital assistant *Vigo in* the home rehabilitation programme?
    area of interest: opinions, views or reflections

To conclude this discussion, I would like to thank you again for your interest and time!

I would also like to invite you to take just 5 more minutes of your time to fill in the short questionnaire that we will send to each of you after this call.

Thank you!

Focus group data

Date:

Venue:

Duration:

Participants:

Focus **group notes:**
